# Structure and function of aerotolerant, multiple-turnover THI4 thiazole synthases

**DOI:** 10.1042/BCJ20210565

**Published:** 2021-09-07

**Authors:** Jaya Joshi, Qiang Li, Jorge D. García-García, Bryan J. Leong, You Hu, Steven D. Bruner, Andrew D. Hanson

**Affiliations:** 1 Department of Horticultural Sciences, University of Florida, Gainesville, FL, U.S.A.; 2 Department of Chemistry, University of Florida, Gainesville, FL, U.S.A.

**Keywords:** comparative genomics, suicide enzyme, thiamin, thiazole biosynthesis

## Abstract

Plant and fungal THI4 thiazole synthases produce the thiamin thiazole moiety in aerobic conditions via a single-turnover suicide reaction that uses an active-site Cys residue as sulfur donor. Multiple-turnover (i.e. catalytic) THI4s lacking an active-site Cys (non-Cys THI4s) that use sulfide as sulfur donor have been biochemically characterized —– but only from archaeal methanogens that are anaerobic, O_2_-sensitive hyperthermophiles from sulfide-rich habitats. These THI4s prefer iron as cofactor. A survey of prokaryote genomes uncovered non-Cys THI4s in aerobic mesophiles from sulfide-poor habitats, suggesting that multiple-turnover THI4 operation is possible in aerobic, mild, low-sulfide conditions. This was confirmed by testing 23 representative non-Cys THI4s for complementation of an *Escherichia coli* Δ*thiG* thiazole auxotroph in aerobic conditions. Sixteen were clearly active, and more so when intracellular sulfide level was raised by supplying Cys, demonstrating catalytic function in the presence of O_2_ at mild temperatures and indicating use of sulfide or a sulfide metabolite as sulfur donor. Comparative genomic evidence linked non-Cys THI4s with proteins from families that bind, transport, or metabolize cobalt or other heavy metals. The crystal structure of the aerotolerant bacterial *Thermovibrio ammonificans* THI4 was determined to probe the molecular basis of aerotolerance. The structure suggested no large deviations compared with the structures of THI4s from O_2_-sensitive methanogens, but is consistent with an alternative catalytic metal. Together with complementation data, use of cobalt rather than iron was supported. We conclude that catalytic THI4s can indeed operate aerobically and that the metal cofactor inserted is a likely natural determinant of aerotolerance.

## Introduction

**Figure 1.**
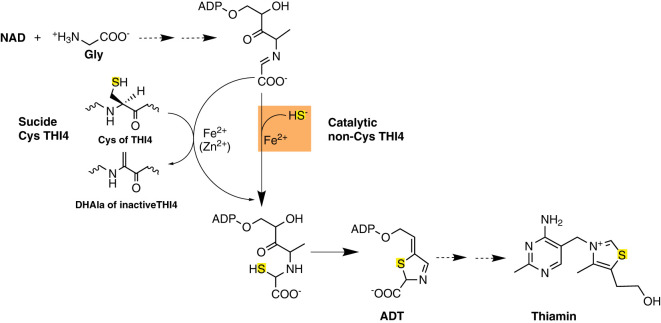
Biosynthesis of the thiazole precursor of thiamin by suicide and catalytic THI4s. THI4s form the adenylated carboxythiazole (ADT) precursor of thiamin from NAD, glycine, and a sulfur atom that in yeast and plant THI4s comes from an active-site cysteine residue and in methanococcal THI4s comes from sulfide (HS^−^). Sulfur loss from the active-site cysteine leaves a dehydroalanine (DHAla) residue that is not reconverted to cysteine, making yeast and plant THI4s suicide enzymes. In contrast, THI4s that use sulfide mediate multiple turnovers, i.e. are true catalysts.

Biosynthesis of the adenylated carboxythiazole (ADT) precursor of thiamin is chemically complex and energetically expensive [1,2]. Plants, fungi, and some prokaryotes make ADT via the thiazole synthase THI4, a single-turnover suicide enzyme [3–6]. In a reaction requiring iron (yeast) or zinc (*Arabidopsis*), these THI4s form ADT from NAD, glycine, and a sulfur atom stripped from an active-site Cys residue [3,5,7,8]. The sulfur loss converts Cys to dehydroalanine and irreversibly inactivates the enzyme [3,5] (Figure 1). Such THI4s must, therefore, be replaced after just one reaction cycle, and this — plus the high demand for thiazole [9] — makes THI4 one of the shortest-lived proteins in plant leaves [10,11].

Bioenergetic calculations indicate that the cost of THI4 degradation and resynthesis in plants reduces biomass accumulation by 2–4% [2]. Crop biomass gains of this order could, therefore, result from engineered replacement of a suicide THI4 with a catalytic THI4 that, like most enzymes, mediates thousands of turnovers in its lifetime [12,13]. But do catalytic THI4s that can operate in the mild, aerobic conditions typical of plant cells exist in nature? And if so, what characteristics confer this ability?

Catalytic THI4s have been demonstrated biochemically from strictly anaerobic, O_2_-sensitive, thermophilic methanogens from hydrothermal vents, where sulfide levels are high enough (millimolar) to kill plants and most other organisms [7,14]. These THI4s use sulfide as the sulfur donor, have His in place of Cys in the active site, and prefer iron as cofactor *in vitro* (Figure 1) [7,14]. Indirect evidence suggests that catalytic, O_2_-sensitive THI4s may also occur in certain plants [5]. An exploratory survey [15] of prokaryote genomes identified THI4s with no active-site Cys (non-Cys THI4s) in several diverse organisms, and preliminary tests showed that two such THI4s complemented an *Escherichia coli* thiazole synthase (Δ*thiG*) mutant in aerobic conditions [15]. By indicating that non-CysTHI4s can have at least some activity in mild conditions in the presence of O_2_, these pilot data prompted further research.

In this work, we deeply surveyed the diversity and genomic contexts of prokaryote non-Cys THI4s and ran complementation assays of thiazole synthase activity on a representative set. In addition, the crystal structure of a THI4 with aerobic complementing activity was determined and this THI4's *in vivo* metal preference was explored. The results implicated the cofactor metal as a determinant of O_2_ tolerance.

## Materials and methods

### Chemicals and media

Chemicals and reagents were from Sigma–Aldrich or Fisher Scientific unless otherwise indicated. MOPS minimal medium was prepared as described [16] except that it was supplemented with the concentrations of micronutrients as specified in [17].

### Bioinformatics

Microbial THI4 sequences were identified in the SEED [18] and UniRef90 [19] databases using *Thermovibrio ammonificans* THI4 as the query sequence. Comparative genomics analyses were performed using SEED and GenBank resources. Sequence similarity networks (SSNs) were constructed by submitting 199 THI4 sequences to the EFI-EST webtool using the FASTA option [20]. An *E-*value of 10^−5^ was used to delimit sequence similarity. A final SSN was generated with an alignment score cutoff set such that each connection (edge) represents ∼80% sequence identity. In this setting, some sequences remained as singletons. Network layouts were created and visualized using Cytoscape 3.4.

### Knockout strain and clone construction

An *E. coli* MG1655 Δ*thiG* strain was made by recombineering [15,21] using the Δ*thiG* cassette from the corresponding Keio collection strain [22]. Selected THI4 genes were recoded for expression in *E. coli* or yeast and synthesized by GenScript (Piscataway, NJ) or Twist Biosciences (San Francisco, CA). Recoded nucleotide sequences are given in Supplementary Table S1. For *E. coli*, recoded sequences with an added N-terminal His_6_ tag were cloned between the EcoRI and XbaI sites in pBAD24 [23]. For yeast (*Saccharomyces cerevisiae*), the recoded *T. ammonificans* THI4 sequence (preceded by the putative yeast THI4 targeting peptide MSATSTATSTSASQLHLNSTPVTHCLSDGG plus a GG linker) or the native yeast THI4 was inserted into the *CEN6/ARS4* nuclear plasmid carrying the *HIS3* marker and the TDH3 promoter to drive THI4 expression.

### THI4 protein expression analysis

pBAD24 constructs were introduced into the *E. coli* MG1655 Δ*thiG* strain and single colonies were used to inoculate 3 ml of MOPS medium containing 0.2% (w/v) glycerol, 100 nM thiamin, 100 μg/ml ampicillin, and 50 μg/ml kanamycin. The next day, 25-ml cultures were grown at 37°C in MOPS-glycerol medium with 100 nM thiamin supplementation until OD_600nm_ reached 0.8. Cells were then induced by adding 0.02% (w/v) arabinose and incubated for another 4 h at 37°C. Cells were harvested by centrifugation (6000***g***, 15 min, 4°C), flash-frozen in liquid nitrogen, and stored at −80°C. Cell pellets were extracted by sonicating in 1 ml of 100 mM potassium phosphate (pH 7.2) containing 2 mM β-mercapto­ethanol, and separated into soluble and insoluble fractions by centrifugation (17 000***g***, 10 min, 4°C). Proteins in the pellet were solubilized by boiling for 5 min in 0.5 ml of SDS sample buffer. Aliquots (10 µl) of the insoluble fraction extract or 10-fold-diluted soluble protein extract were separated by SDS–PAGE on 15% gels; proteins were detected by Coomassie Blue staining. The identity of the THI4 bands was confirmed by immunoblotting using anti-His_6_ tag antibodies (Thermo Fisher Scientific MA1-21315). The THI4 Coomassie band in soluble and insoluble fractions was quantified using Licor Image Studio Lite software. A 3441-pixel area around the band was used to calculate signal intensity. The method was calibrated using a standard curve for purified recombinant *T. ammonificans* THI4.

### Functional complementation assays in *E. coli* and yeast

For assays in *E. coli*, three independent clones of each construct were used to inoculate 3 ml of MOPS med­ium containing 0.2% glycerol (w/v), 100 nM thiamin, and 50 μg/ml kanamycin. After incubation at 37°C for 18 h, cells were harvested by centrifugation, washed five times with thiamin-free MOPS medium, resuspended in 500 μl of the same medium, serially diluted in 10-fold steps, and spotted on MOPS medium plates containing 0.2% glycerol, 0.02% arabinose, plus or minus 1 mM Cys. Plates were then incubated at 37°C in aerobic and near-anerobic (N_2_ containing ∼1 ppm O_2_) conditions as described [15]. Images were captured after 7 d. For complementation assays with *T. ammonificans* THI4 in yeast, three independent clones of strain Δ*THI4* BY4741 (*MATa his3*Δ*1 leu2*Δ*0 met15*Δ*0 ura3*Δ*0*; *THI4*Δ::KanMX) transformed with the *CEN6/ARS4* plasmid alone or containing *T. ammonificans* THI4 or yeast THI4 were used to inoculate 3 ml of synthetic, complete medium (SC; yeast nitrogen base, USBiological cat. no. Y2036), drop-out mix (USBiological cat. no. D9540) minus histidine supplemented with 20 g/l glucose, 5 g/l ammonium sulfate, and 300 nM thiamin. After 48 h of incubation at 30°C and 220 rpm, cells were pelleted (3000***g***, 5 min), washed five times with thiamin-free SC minus histidine medium, resuspended in the same medium, and used to inoculate 3 ml of thiamin-free SC minus histidine medium to an OD_600nm_ of 0.05. Growth was then monitored at OD_600nm_.

### Purification and anaerobic reconstitution of *T. ammonificans* THI4

*T. ammonificans* THI4 cloned in pBAD24 with an N-terminal His_6_ tag was transformed into *E. coli* BL21(DE3). A starter culture (15 ml LB plus 100 µg/ml ampicillin) was inoculated into 6 l of LB; THI4 expression was initiated at an OD_600_ of 0.6 with arabinose (0.02% w/v) and incubation was continued at 22°C for 20 h. Cells were then harvested by centrifugation; pellets were resuspended in 50 ml of lysis buffer (20 mM Tris–HCl, pH 8.0, 500 mM NaCl, 2 mM 2-mercaptoethanol) and lysed in a microfluidizer cell (14 000 psi, M-110L Pneumatic). The lysate was clarified by centrifugation (18 000***g***, 30 min, 4°C) and applied to a 1-ml Ni-NTA column (HisPur, ThermoFisher Scientific). After incubation at 4°C for 1 h the resin was washed with 50 ml of lysis buffer and eluted with 6 ml lysis buffer plus 250 mM imidazole. The eluate was dialyzed against 1 l of 20 mM Tris–HCl, pH 8.0, 100 mM NaCl, 2 mM 2-mercaptoethanol for 12 h and purified by anion exchange chromatography (HiTrap Q, GE Healthcare) with a linear gradient of 0–500 mM NaCl over 40 min and size-exclusion chromatography (HiLoad 16/600 Superdex 200, GE Healthcare) in 20 mM Tris–HCl, pH 8.0, 100 mM NaCl, 2 mM 2-mercaptoethanol. Purified protein fractions were concentrated to 500 μl (14 mg/ml) and transferred to an anaerobic glovebox. Protein reconstitution and crystallization experiments were performed under pure argon and all solutions used were degassed and purged with argon before use. Purified enzyme (500 μl, 14 mg/ml) was incubated with 10 molar equivalents of ferrous ammonium sulfate at 22°C for 30 min, followed by incubation with 10 molar equi­valents each of NAD^+^ and glycine for 1 h.

### Crystallization and structure solution of *T. ammonificans* THI4

Initial crystallization conditions were obtained using the hanging-drop method at 22°C, with 2 μl reconstituted enzyme and 2 μl of 100 mM HEPES-NaOH, pH 7.5, 200 mM NaCl and 35% 2-methyl-2,4-pentanediol. Optimization of crystal morphology resulted in cubic shaped crystals in ∼2 weeks with 100 mM HEPES-NaOH, pH 7.5, 200 mM NaCl, 35% 2-methyl-2,4-pentanediol, 10 mM praseodymium acetate hydrate as the precipitant.

### Data collection, processing, and structure refinement

Diffraction data were collected on beamline 23-ID-D of LS-CAT at Argonne National Laboratory Advanced Photon Source at a wavelength of 1.033 Å. Data were collected at 100°K and processed using XDS [24] to 2.3 Å resolution in space group I121 (Supplementary Table S2). The structure was solved by molecular replacement using a homology model of *Methanococcus igneus* THI4 (PDB: 4Y4N, ∼50% sequence identity). A model of *T. ammonificans* THI4 (four monomers per asymmetric unit) was iteratively built by combining AutoBuild (PHENIX [25]) with manual building in COOT [26]. Structure refinement was performed in PHENIX and REFMAC [25,27]. The parameter file for the bound glycine imine intermediate was generated using phenix.elbow [28]. The co-ordinates and structure factors are available from the Protein Data Bank (PDB ID: 7RK0).

## Results

### Diversity of non-Cys THI4s and selection of representatives

We first surveyed ∼1000 prokaryotic THI4 proteins in the SEED [18] and UniRef90 [19] databases using BlastP and non-Cys THI4s [15] as query sequences. After removing entries that were truncated, redundant, or from unidentified organisms, there remained 199 unique sequences in which the active-site Cys position was occupied by His (171 cases) or by Met, Leu, Pro, Ala, Ser, Glu, Asp, Tyr, or Trp (28 cases) (Supplementary Table S3). The 199 sequences shared only 47% identity on average.

**Figure 2.**
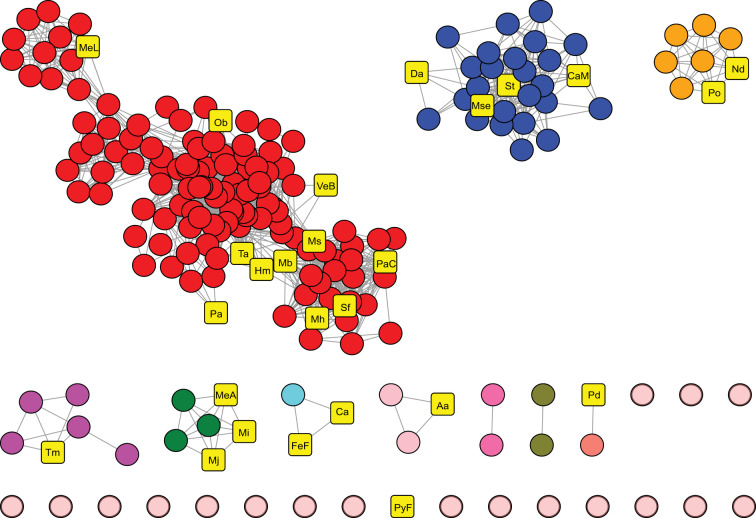
Sequence similarity network (SSN) of 199 diverse non-Cys THI4s. Each node in the SSN corresponds to a single sequence; each edge (gray lines) represents a pair-wise connection between two sequences at a BLAST *E*-value < 1 × 10^−5^. Lengths of edges are not significant, except that tightly clustered groups share more similarity than sequences with only a few connections. The 26 representative sequences selected for testing are shown as yellow squares; organism name abbreviations are as in Table 1.

To analyze this sequence diversity and help select representatives to test for activity, we built SSNs using the Enzyme Function Initiative webtool [20,29]. The final SSN (*E-*value = 10^−5^, alignment score = 80) contained a series of clusters plus various singletons (Figure 2). Some clusters included non-Cys THI4s that have been tested for activity [7,14,15] but others did not, indicating that much non-Cys THI4 sequence space remained to be sampled.

**Table 1.** Ecophysiology of the prokaryote sources of the THI4s selected for testing

Organism (abbreviation)	Natural habitat^1^	O_2_ adaptation	Temperature	Sulfide^1^^,^^2^
THI4s with readily detectable activity				
Bacteria				
*Fervidicola ferrireducens* (FeF)	Thermal aquifer	Obligate anaerobe	Thermophile	Low
*Thermovibrio ammonificans* (Ta)	Hydrothermal vent	Anaerobe	Thermophile	High
*Hippea maritima* (Hm)	Hydrothermal vent	Anaerobe	Thermophile	High
*Thermotoga maritima* (Tm)	Hot marine sediment	Anaerobe	Thermophile	High
*Caldanaerovirga acetigignens* (Ca)	Hot springs	Anaerobe	Thermophile	High
*Mucinivorans hirudinis* (Mh)	Leech gut	Anaerobe	Mesophile	High
*Parabacteroides chinchillae* (PaC)	Chinchilla gut	Anaerobe	Mesophile	High
*Verrucomicrobia* bacterium (VeB)	Soil	Anaerobe	Mesophile	Low
*Pseudoramibacter alactolyticus* (Pa)	Abscesses	Anaerobe	Mesophile	High
*Marinilabilia salmonicolor* (Ms)	Marine mud	Facultative aerobe	Mesophile	High
*Saccharicrinis fermentans* (Sf)	Marine mud	Facultative aerobe	Mesophile	High
*Candidatus* Marinimicrobia (Mb)	Sea water column	Facultative aerobe	Mesophile	Low
Archaea				
*Pyrodictium delaneyi* (Pd)	Hydrothermal vent	Obligate anaerobe	Thermophile	High
*Methanococcus jannaschii* (Mj)	Hydrothermal vent	Obligate anaerobe	Thermophile	High
*Methanococcus aeolicus* (MeA)	Marine sediments	Obligate anaerobe	Mesophile	High
*Methanococcus igneus* (Mi)	Hydrothermal vent	Anaerobe	Thermophile	High
THI4s with little to no detectable activity				
Bacteria				
*Candidatus* Omnitrophica (Ob)	Hydrothermal vent	Anaerobe	Thermophile	High
*Poribacteria* sp. WGA-A3 (Po)	Sponge symbiont	Facultative aerobe	Mesophile	Low
*Nitrospira defluvii* (Nd)	Sewage sludge	Aerobe	Mesophile	High
Archaea				
*Desulfurococcus amylolyticus* (Da)	Hot springs	Obligate anaerobe	Thermophile	High
*Candidatus* Aenigmarchaeota (Aa)	Hot springs	Anaerobe	Thermophile	High
*Pyrolobus fumarii* (PyF)	Hydro­thermal vent	Facultative microaerobe	Thermophile	High
*Metallosphaera sedula* (Mse)	Acidic hot springs	Aerobe	Thermophile	High
Poorly expressed archaeal THI4s^c^				
*Sulfurisphaera tokodaii* (St)	Hot springs	Obligate aerobe	Thermophile	High
*Methanofollis liminatans* (MeL)	Wastewater bioreactor	Obligate anaerobe	Mesophile	High
*Caldivirga maquilingensis* (CaM)	Acidic hot spring	Facultative microaerobe	Thermophile	High

1 Information was extracted or inferred from literature reporting the isolation or description of the organisms.

2 Broad estimates. Low sulfide: low micromolar range. High sulfide: high micromolar to low millimolar range.

3 Expressed poorly in soluble form in *E. coli* and therefore not analyzed further.

We selected 26 representative sequences from throughout the SSN and from organisms with different ecophysiologies (Figure 2 and Table 1). These sequences were about as diverse (45% average identity) as the whole set of 199 and included THI4s described previously [7,14,15]. Fifteen of the selected sequences were bacterial and 11 were archaeal, eight were from organisms that require or tolerate O_2_, 11 were from mesophiles, 15 were from thermophiles, 22 were from habitats known or likely to be sulfide-rich, and four were from habitats likely to be sulfide-poor (Table 1).

### Genomic context of non-Cys THI4 genes

**Figure 3.**
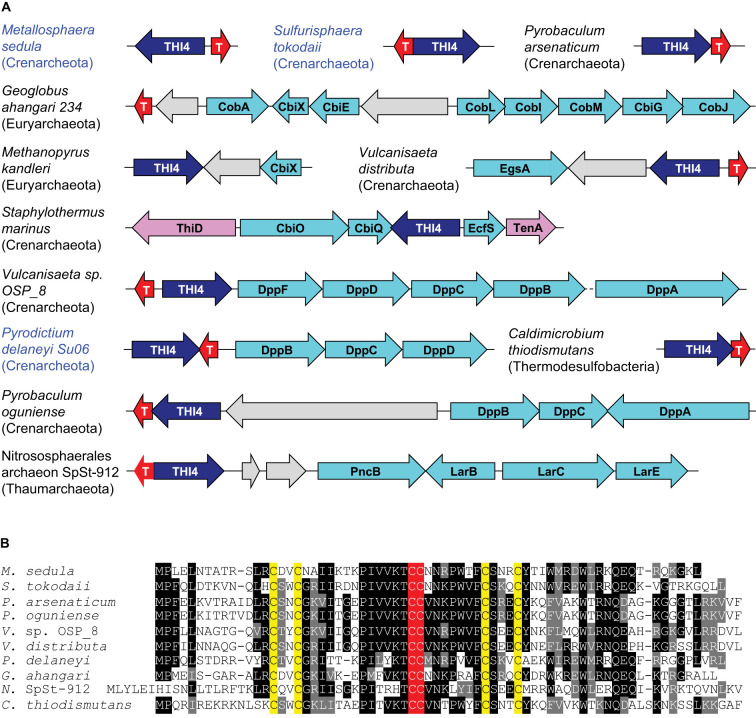
Association of THI4 genes with TRASH genes and cobalt- or nickel-related genes. (**A**) Chromosomal gene clustering arrangements. Names of organisms whose THI4s were tested are in blue. T, TRASH protein gene. Genes colored aqua encode homologs of nickel- and/or cobalt-related proteins: CobA, uroporphyrin-III C-methyltransferase, involved in cobalamin and sirohaem synthesis; CbiX, sirohydrochlorin nickel/cobalt chelatase, which inserts nickel or cobalt into tetrapyrroles; CbiE, involved in cobalamin synthesis; a cobalamin synthesis operon encoding CobL, CobI, CobM, CbiG, CobJ, plus (not shown) CbiC and CbiD; CbiO, CbiQ, and EcfS, the A + A′, T, and S components of a cobalt energy coupling-factor (ECF) transporter. DppA-D,F, subunits of ABC transporters for oligo­peptides, nickel, or (rarely) cobalt; LarBCE, nickel-pincer nucleotide (NPN) cofactor synthesis genes; PncB, synthesis enzyme for the NPN precursor NaAD; EgsA, glycerol-1-phosphate dehydrogenase, which has a nickel or zinc cofactor; gray genes have no known nickel or cobalt associations. Genes colored lilac encode thiamin synthesis or salvage enzymes. (**B**) Alignment of the TRASH proteins encoded by genes in part A. The Cys residues of the extended TRASH motif (Cys-Xaa_2_-Cys-Xaa_19–22_-Cys-Xaa_3_-Cys) are in yellow; the two extra Cys residues are in red.

The selected THI4s all came from genomes that encode ThiE or ThiN (Supplementary Figure S1), indicating capacity to synthesize thiamin from its thiazole and pyrimidine precursors. All genomes also had ThiD and all but one had ThiC (Supplementary Figure S1), respectively, indicating capacity to utilize or produce the pyrimidine precursor. Nearly all the selected THI4s, therefore, came from organisms with complete thiamin synthesis pathways. Only six genomes encoded the alternative thiazole synthase ThiG, so that THI4 was typically the only identifiable endogenous source of the thiazole precursor. Three of the selected archaeal *THI4* genes abut a gene coding for a protein from the TRASH family, whose members bind nonferrous heavy metals, with characterized examples involved in zinc, copper, cadmium, and/or mercury transport/resistance [30–35] (Supplementary Figure S1). Similar clustering occurs in many other archaea and bacteria (Figure 3A). The TRASH proteins in these clusters have two adjacent Cys residues in addition to the four canonical metal-binding Cys residues in the TRASH motif (Cys-Xaa_2_-Cys-Xaa_19–22_-Cys-Xaa_3_-Cys) [30] (Figure 3B); these extra Cys could help ligand a six-co-ordinate metal such as cobalt, nickel, or iron. Furthermore, non-Cys THI4 and TRASH genes cluster with genes for proteins from families that transport or metabolize cobalt or nickel [36–39] (Figure 3A). Non-Cys THI4s are thus more strongly genomically associated with nonferrous metals than with iron.

### Functional complementation tests of THI4 activity

**Figure 4.**
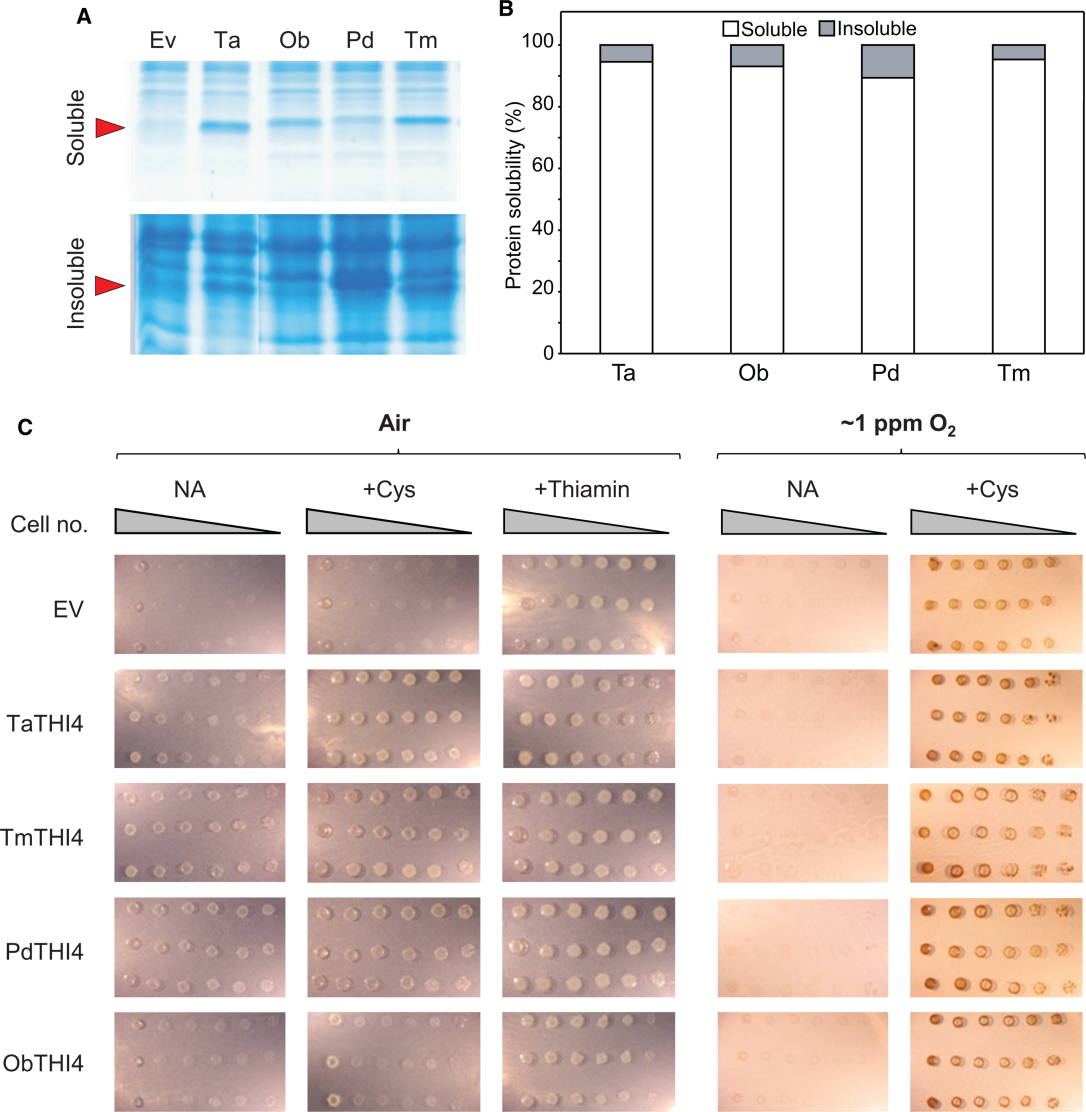
Soluble expression and functional complementation tests of non-Cys THI4s. (**A**) Gel analysis of soluble and insoluble expression in *E. coli* of representative THI4s. Soluble and insoluble fractions of cells were run on 15% gels, stained with Coomassie blue, and scanned to quantify the THI4 band, for which purified *Thermovibrio ammonificans* (Ta) THI4 served as a marker (arrow). Organism abbreviations are as in Table 1. (**B**) Quantification of the solubility (%) of the THI4s from (**A**). (**C**) Tests of functional complementation of an *E. coli* Δ*thiG* strain by the THI4s from (**A**) or the empty vector (EV). TaTHI4, TmTHI4, and PdTHI4 had clearly detectable activity in air while ObTHI4 did not; none had more than slight activity in ∼1 ppm O_2_. Overnight cultures of three independent clones per construct were 10-fold serially diluted and spotted on plates of MOPS minimal medium containing 0.2% (w/v) glycerol and 0.02% (w/v) arabinose with no additions (NA) or plus 1 mM Cys or 100 nM thiamin. The medium used for culture in ∼1 ppm O_2_ contained 40 mM nitrate as electron acceptor. Cells were cultured in air or under N_2_ containing ∼1 ppm O_2_. Images were captured after incubation at 37°C for 7 d. The high background in the ∼1 ppm O_2_ +Cys treatment is due to staining of the inoculum cells.

The 26 selected THI4s were recoded for *E. coli* and inserted into pBAD24 [23]; the resulting constructs were then introduced into an *E. coli* Δ*thiG* strain [15]. To check THI4 expression, cells were grown in thiamin-supplemented minimal medium and harvested for gel analysis of the soluble and insoluble fractions (Figure 4A,B and Supplementary Figure S2). Of the 26 THI4s, 23 expressed well, with ≥85% in soluble form (Table 1) and were advanced to testing for thiazole synthase activity by complementation.

Complementation tests were run in air or in N_2_ containing ∼1 ppm O_2_, plus or minus supplementation with 1 mM Cys to increase intracellular sulfide level [40]. Sixteen strains showed clear growth in air, particularly when supplemented with Cys while seven did not, and no strain showed clear growth in ∼1 ppm O_2_ but not in air (Figure 4C and Supplementary Figure S3). The enhanced growth with Cys supplementation fits with use of sulfide as sulfur donor [5,15]. The complementation tests in air thus split the THI4s into one group with readily detectable activity (henceforth: aerotolerant THI4s) and another with little or none. Both groups included THI4s from mesophiles and thermophiles, aerobes and anaerobes, and organisms from high- and low-sulfide habitats (Table 1). There was hence no evident correlation between THI4 aerotolerance and source organism ecophysiology. Neither was aerotolerance correlated with the residue (His, Met, Leu, Tyr, or Ser) that replaces Cys in the active site or with the number of Cys and Met residues, which can affect oxidative instability [41,42] (Supplementary Table S3). To summarize: the complementation data establish that some catalytic THI4s operate quite well (although not optimally, see below) in aerobic conditions but do not suggest why others do not. One of many possible causes is that the redox environment in *E. coli* differs enough from that in the THI4 source, especially for extremophiles (Table 1) [43–45], to disrupt protein disulfide formation or metal insertion and coordination [45–47] and thus prevent THI4 expression in its native form.

It is important to note that supplementation with thiamin markedly stimulated the growth of all strains (compare NA and + Thi columns in air in Figure 4C and Supplementary Figure S3). This stimulation shows that the *in vivo* activity of THI4s with complementing activity did not fully meet the demand for ADT despite their high expression levels (Figure 4A and Supplementary Figure S2), i.e. that there is room for activity improvement, at least when no Cys is supplied. We revisit this point later.

### Protein structure of the aerotolerant *T. ammonificans* THI4

As structural data for THI4s with little or no complementing activity in air would be hard to interpret in terms of aerotolerance alone since failure has other possible causes, we explored molecular features associated with aerotolerance by determining the crystal structure of the complementation-active THI4 from *T. ammonificans* (TaTHI4) (PDB: 7RK0). This bacterium is an anaerobe but is likely to be intermittently exposed to O_2_ in its habitat near the oxic/anoxic interface in hydrothermal vents, and encodes enzymes to detoxify reactive oxygen species (ROS), including catalase/peroxidase, cytochrome *c* peroxidase, and cytochrome *bd* complex [48,49]. This makes the TaTHI4 structure a potentially informative ‘routinely O_2_-exposed’ comparison with the structures of ‘never O_2_-exposed’ THI4s from the archaeal methanogens *M. igneus*, *M. jannaschii*, and *Methanothermococcus thermolithotrophicus* [7,50]. Like *T. ammonificans*, these organisms are anaerobic thermophiles from hydrothermal vents [48,51] but, unlike *T. ammonificans*, they inhabit the anoxic region of the vent plume and lack the ROS defense genes present in *T. ammonificans* as well as heme- and manganese-catalase genes (Supplementary Table S4). Furthermore, *M. igneus* and *M. jannaschii* THI4s had similar complementing activity in *E. coli* to TaTHI4 (Supplementary Figure S3), meaning that differences among their recombinant protein structures are unlikely to be artifacts of misfolding in the heterologous host. In comparing the TaTHI4 structure with those of the three archaeal methanogens we made the reasonable assumption that these non-Cys THI4s are all catalytic, as *M. jannaschii* THI4 has been shown to be [14].

**Figure 5.**
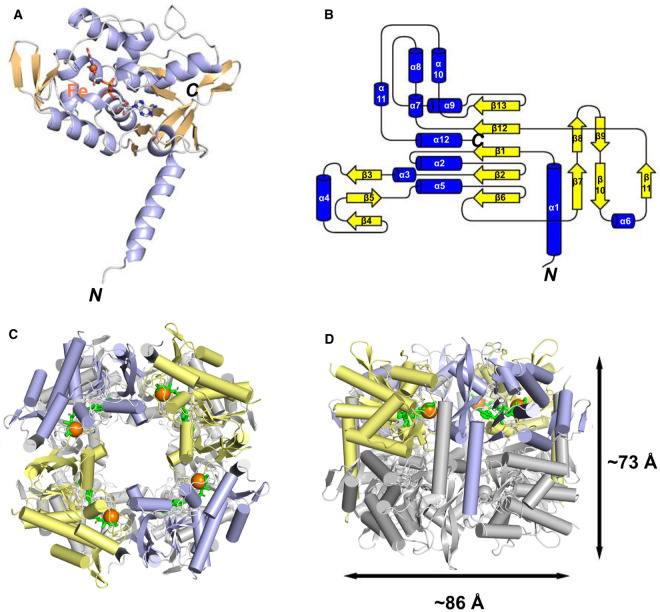
Structure of the aerotolerant non-Cys THI4 from *Thermovibrio ammonificans*. (**A**) Ribbon diagram of the TaTHI4 monomer with bound glycine imine shown as sticks; α-helices are colored blue, β-strands are colored beige; Fe(II) is shown as a brown sphere. (**B**) Topology diagram of TaTHI4 with α-helices in blue and β-strands in yellow. (**C**,**D**) The biologically relevant TaTHI4 homooctamer shown in ribbon representation in alternate top and side views with the metal Fe(II) indicated as orange spheres and the glycine imine as green sticks. One tetramer is blue/yellow and the other is colored gray.

Protein crystals of TaTHI4 diffracted to 2.3 Å resolution in space group I121 and the structure was solved by molecular replacement (Supplementary Table S2) using *M. igneus* THI4 as a search model (PDB: 4Y4N) [7]. In common with the methanogen THI4s, TaTHI4 is an overall homooctamer with four monomers per asymmetric unit (Figure 5A). The octamer is tightly packed as a two-layer ring structure with approximate dimensions 73 × 86 Å (height × width) enclosing a ∼26 Å diameter pore. The monomer structure (Figure 5B) consists of a long α-helix (residues 6–24) and a barrel-like core domain sandwiched by helix bundles (Figure 5C). The barrel-like core comprises a central five-stranded β-sheet with β6, β2, β1, β12, β13 topology, flanked by a twisted antiparallel β-sheet (β7, β10, β11) connecting α6 and a four-helix bundle (α9, α12, α2, α5). The barrel core is capped by a variation of the Rossmann fold β-hairpin motif. The C-terminal topology is similar to the canonical Rossman fold with an inversion of strands. TaTHI4 is thus structurally homologous to the previously solved methanogen THI4s [7,50].

**Figure 6.**
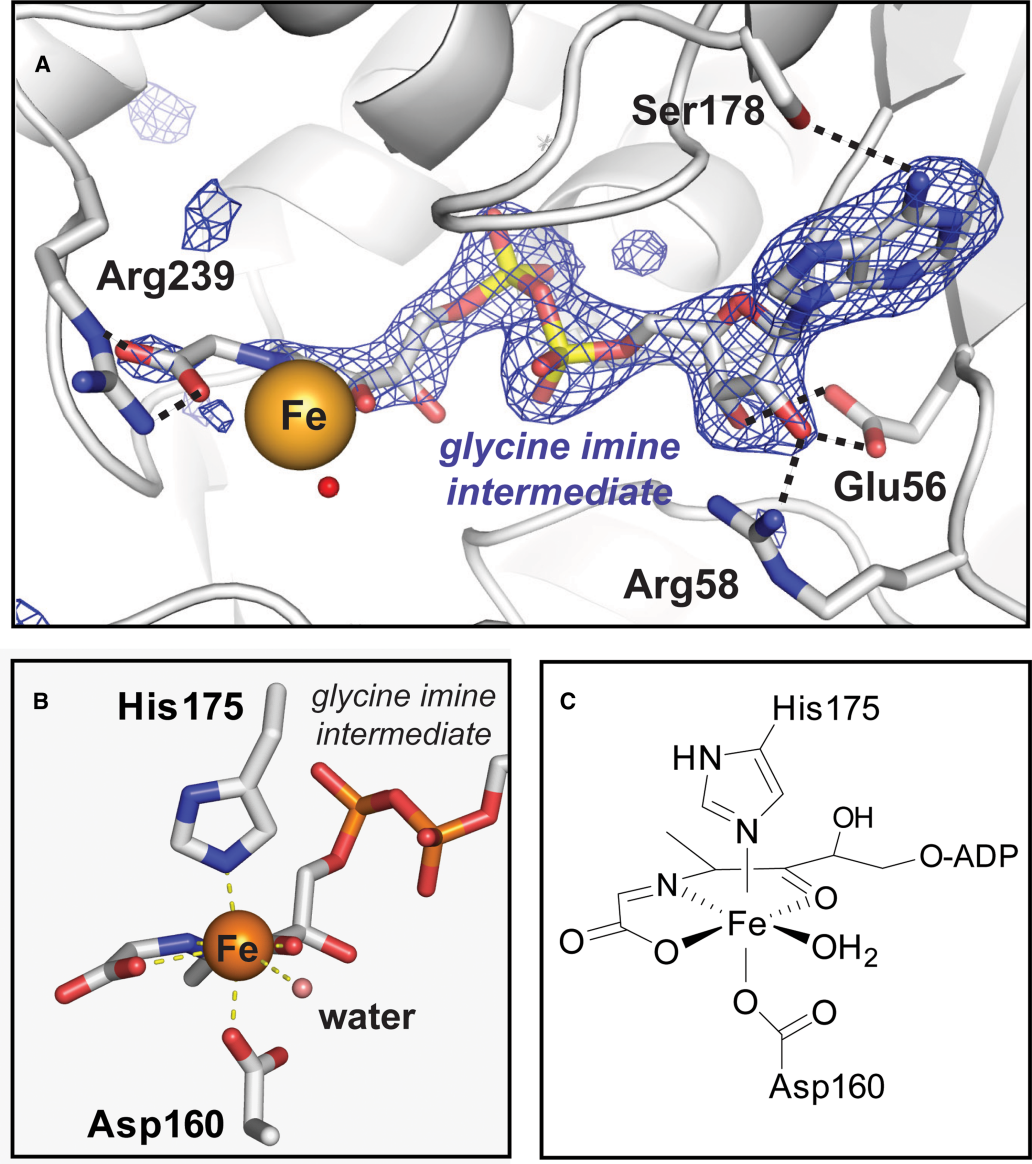
The active site of *Thermovibrio ammonificans* THI4. (**A**) Interactions of the bound glycine intermediate with the TaThi4 protein active site. A glycine imine intermediate omit map, Fo–Fc contoured at 3.5σ, is shown in blue. (**B**) Ligand geometry around the metal center with a near-octahedral coordination environment as diagrammed in (**C**).

The enzyme active site lies in a channel formed by two helix bundles and is capped by a loop from an adjoining monomer. Clear density for the TaTHI4 treated with NAD and glycine shows the expected glycine imine intermediate [7] in the active site (Figure 6A). This intermediate adopts an elongated conformation with the adenine moiety positioned in the barrel-like core domain. Binding interactions between the intermediate and TaTHI4 include a hydrogen bond between N6 of adenine and the side chain of Ser178, a bidentate hydrogen bond with the 2′ and 3′ hydroxyls and Glu56, a second hydrogen bond between Arg58 and the 2′ hydroxyl, along with a stabilizing interaction of the glycine imine carboxylate and Arg239 (Figure 6A). Based on our reconstitution conditions, the bound metal in the active site can be assigned as ferrous iron, Fe(II). The iron is co-ordinated in a pseudooctahedral geometry with three sites occupied by the pincer-type ligand of the bound glycine imine, axial ligands of His175 and Asp160, and a predicted water molecule (Figure 6B,C).

To probe the role of the Met158 residue that in TaTHI4 occupies the position of the sacrificial Cys, we changed Met158 to His, Lys, or Cys and assayed complementing activity (Supplementary Figure S4). The mutant enzymes performed similarly to wild-type TaTHI4 in both air and ∼1 ppm O_2_ atmospheres, with or without Cys supplementation. This result confirms the inference from natural variation in this residue (Supplementary Table S3) that it has no role in catalysis. That the Cys form did not have greater activity in the absence of Cys supplementation suggests that it cannot operate efficiently in suicide mode and still depends on sulfide as sulfur source, as was the case for *M. jannaschii* THI4 [7].

### Investigation of TaTHI4 metal preference *in vivo* in *E. coli* and yeast

As we could not obtain quantifiable *in vitro* activity [14] from reconstituted TaTHI4, we sought to test metal cofactor preference *in vivo*. Adding 100 µM cobalt, nickel, zinc, or manganese to the medium of *E. coli* expressing TaTHI4 (or five other THI4s) did not detectably affect complementing activity in air (Supplementary Figure S5). This result does not indicate which metal is preferred, but does show that the supply of this metal is not limiting in *E. coli* grown with normal trace metal supplementation.

We also tested metal preference using yeast, which resembles *E. coli* in having native cobalt-dependent enzymes and cobalt uptake systems [52] but differs in having no native nickel enzymes or high-affinity nickel uptake system and in needing an added nickel transporter to express a foreign nickel enzyme in active form [53]. We, therefore, tested TaTHI4 for the ability to complement a yeast Δ*THI4* strain. The observed complementation (Supplementary Figure S6) proves that TaThi4 has access to its metal cofactor and implies that this metal is more likely to be cobalt than nickel if it is not iron.

## Discussion

The biochemically characterized catalytic THI4s come from archaea of anoxic, sulfide-rich, reducing environments, use an O_2_-sensitive sulfur donor (sulfide) and cofactor (ferrous iron), and are thermophilic [7,14]. It was consequently *a priori* doubtful whether catalytic prokaryotic THI4s could function in mild, fully aerobic conditions, and recent evidence [5] that putatively catalytic plant THI4s are expressed only at severely hypoxic stages of seed development reinforced this doubt.

However, our survey of THI4 sequences and their provenances began to dispel the doubt because non-Cys THI4s confidently predicted from genomic context to be functional thiazole synthases were found in aerobic or aerotolerant organisms from mild environments. Confirmation that certain non-Cys THI4s — the majority of those tested, in fact — can function in air came from complementation experiments, which also indicated that these THI4s use sulfide, or a sulfide metabolite, as sulfur donor.

Having established that certain non-Cys THI4s can — unexpectedly — operate in mild, fully aerobic conditions, we investigated characteristics that enable them to do so. We could dismiss any role, positive or negative, for thermophily because complementing activity varied similarly in thermophiles and mesophiles (Table 1). There was likewise no association between complementing activity and the O_2_ adaptation of the source organism (Table 1). Nor did the residue that replaces the sacrificial Cys or the number of Cys or Met residues appear to be important (Supplementary Table S3).

What then, might confer aerotolerance? The comparative genomic analysis provided a clue by associating non-Cys THI4s with proteins that bind, transport, or metabolize nonferrous transition metals, notably cobalt or nickel (Figure 3A), and the observed complementing activity of TaTHI4 in yeast favored the possibility that the metal cofactor is cobalt (Supplementary Figure S6). Neither the comparative genomics nor the yeast complementation data ruled out a ferrous iron cofactor, however.

Comparing the TaTHI4 structure — the first deposited for a bacterial THI4 — with those of the ecologically less O_2_-exposed THI4s from archaeal methanogens (PDBs: 4Y4M, 4Y4N, 6HK1) [7,50] did not provide strong evidence on the aerotolerance phenomenon. The active sites are largely identical, with one major difference, the variation of residues (Met vs. His) replacing the Cys residue of suicide THI4s. Variants at this position, however, do not correlate with aerotolerance (Supplementary Table S3). The octamer structures show similar dimensions of the overall barrel-like architecture and similar surface electrostatic charge. Notable differences are two extended loop regions in TaTHI4 at the entrance to the large active-site pore (residues 134–142 and 186–197); however, based on the pore diameter, these changes are unlikely to affect O_2_ diffusion. An analogous comparision of the structures of O_2_-sensitive and O_2_-tolerant homologs of [NiFe] hydrogenase showed distinct, well-defined tunnels to the metal center [54,55]. In this case, structural variations of the tunnel were predicted to regulate O_2_ diffusion to the reactive metal center. All THI4s have a large pore (∼26 Å) linking the active site to solvent, precluding variation in O_2_ diffusion. The lack of dissimilar structural features favors the alternative possibility of a role for the active site metal in O_2_ tolerance.

Semi-quantitative *in vitro* assay of *M. jannaschii* THI4 activity [14] showed that cobalt supported ∼60%, and nickel ∼25%, of the activity given by iron. The presented TaTHI4 structure contains an obligate bound Fe(II) atom, based on Fe(II) being the only metal present during protein reconstitution. The ligand geometry in the structure (Figure 6) does not preclude other metals, including Co(II) or Ni(II). A bound Ni(II) would be predicted to favor a square pyrimidal geometry [56] over the octahedral although the active site could possibly accommodate a minor shift in ligand position to square pyrimidal. There is solid precedent for octahedral Co(II), including colbalamin enzymes and methylmalonyl-CoA carboxytransferase [57]. In terms of THI4 chemistry, Co(II) is less likely to react deleteriously with molecular O_2_ than Ni(II) and Fe(II). Contrasting with many examples in which mononuclear Fe(II) reacts with O_2_ to generate reactive O-atom transfer species in metalloenzymes and model compounds [58,59], reaction of Co(II) with O_2_ has strong precedent for reversible formation of Co(III)-superoxide species in the absence of additional reducing equivalents or protonation [60], implying that replacing iron with cobalt could provide a mechanism for the observed O_2_ tolerance. In addition, a Ni(II)-SH intermediate in the catalytic cycle would be particularly stable [61], disfavoring catalysis. Other metals are known to be able to perform THI4 chemistry, notably Zn(II), but the ligand geometry and metal preference analysis of *M. jannaschii* THI4 [14] argue against a tetrahedral liganded metal.

In summary, the comparative genomics, functional complementation, and structural evidence collectively implicate the bound metal as a natural determinant of THI4 aerotolerance, with Co(II) the best candidate although Ni(II) cannot be rigorously excluded. Definitive evidence on this point will require the development of quantitative *in vitro* assays for THI4 activity. Besides the nature of the metal inserted, there may well be other determinants of aerotolerance. We hope to identify such determinants, and to gain insight into the metal cofactor, from ongoing continuous directed evolution experiments [62] to improve the complementing activity of native non-Cys THI4s. If — as seems likely [63] — such improvement is possible, there is a realistic prospect of replacing suicidal plant THI4s with catalytic THI4s that work well in aerobic conditions and thus slash the energy cost of thiamin synthesis.

## Data Availability

Co-ordinates and structure factors of the *T. ammonificans* THI4 crystal structure have been deposited in PDB under code 7RK0.

## Open Access

Open access for this article was enabled by the participation of University of Florida in an all-inclusive *Read & Publish* pilot with Portland Press and the Biochemical Society.

## Abbreviations

ADT: adenylated carboxythiazole
IPTG: Isopropyl β-d-1-thiogalactopyranoside
ROS: reactive oxygen species
SSN: sequence similarity network

## References

[1] Begley, T.P., Ealick, S.E. and McLafferty, F.W. (2012) Thiamin biosynthesis: still yielding fascinating biological chemistry. Biochem. Soc. Trans. 40, 555–560 10.1042/BST20120084 22616866 PMC3771315

[2] Hanson, A.D., Amthor, J.S., Sun, J., Niehaus, T.D., Gregory, III, J.F., Bruner, S.D. et al. (2018) Redesigning thiamin synthesis: prospects and potential payoffs. Plant Sci. 273, 92–99 10.1016/j.plantsci.2018.01.019 29907313

[3] Chatterjee, A., Abeydeera, N.D., Bale, S., Pai, P.J., Dorrestein, P.C., Russell, D.H. et al. (2011) *Saccharomyces cerevisiae* THI4p is a suicide thiamine thiazole synthase. Nature 478, 542–546 10.1038/nature10503 22031445 PMC3205460

[4] Faou, P. and Tropschug, M. (2004) *Neurospora crassa* CyPBP37: a cytosolic stress protein that is able to replace yeast Thi4p function in the synthesis of vitamin B1. J. Mol. Biol. 344, 1147–1157 10.1016/j.jmb.2004.09.097 15544818

[5] Joshi, J., Beaudoin, G.A.W., Patterson, J.A., García-García, J.D., Belisle, C.E., Chang, L.Y. et al. (2020) Bioinformatic and experimental evidence for suicidal and catalytic plant THI4s. Biochem. J. 477, 2055–2069 10.1042/BCJ20200297 32441748

[6] Hwang, S., Cordova, B., Chavarria, N., Elbanna, D., McHugh, S., Rojas, J. et al. (2014) Conserved active site cysteine residue of archaeal THI4 homolog is essential for thiamine biosynthesis in *Haloferax volcanii*. BMC Microbiol. 14, 260 10.1186/s12866-014-0260-0 25348237 PMC4215014

[7] Zhang, X., Eser, B.E., Chanani, P.K., Begley, T.P. and Ealick, S.E. (2016) Structural basis for iron-mediated sulfur transfer in archael and yeast thiazole synthases. Biochemistry 55, 1826–1838 10.1021/acs.biochem.6b00030 26919468 PMC4811699

[8] Godoi, P.H., Galhardo, R.S., Luche, D.D., Van Sluys, M.A., Menck, C.F. and Oliva, G. (2006) Structure of the thiazole biosynthetic enzyme THI1 from *Arabidopsis thaliana*. J. Biol. Chem. 281, 30957–30966 10.1074/jbc.M604469200 16912043

[9] Hanson, A.D., Beaudoin, G.A., McCarty, D.R. and Gregory, III, J.F. (2016) Does abiotic stress cause functional B vitamin deficiency in plants? Plant Physiol. 172, 2082–2097 10.1104/pp.16.01371 27807106 PMC5129723

[10] Nelson, C.J., Alexova, R., Jacoby, R.P. and Millar, A.H. (2014) Proteins with high turnover rate in barley leaves estimated by proteome analysis combined with in planta isotope labeling. Plant Physiol. 166, 91–108 10.1104/pp.114.243014 25082890 PMC4149734

[11] Li, L., Nelson, C.J., Trosch, J., Castleden, I., Huang, S. and Millar, A.H. (2017) Protein degradation rate in *Arabidopsis thaliana* leaf growth and development. Plant Cell 29, 207–228 10.1105/tpc.16.00768 28138016 PMC5354193

[12] Tivendale, N.D., Hanson, A.D., Henry, C.S., Hegeman, A.D. and Millar, A.H. (2020) Enzymes as parts in need of replacement – and how to extend their working life. Trends Plant Sci. 25, 661–669 10.1016/j.tplants.2020.02.006 32526171

[13] Hanson, A.D., McCarty, D.R., Henry, C.S., Xian, X., Joshi, J., Patterson, J.A. et al. (2021) The number of catalytic cycles in an enzyme's lifetime and why it matters to metabolic engineering. Proc. Natl Acad. Sci. U.S.A. 118, e2023348118 10.1073/pnas.2023348118 33753504 PMC8020674

[14] Eser, B.E., Zhang, X., Chanani, P.K., Begley, T.P. and Ealick, S.E. (2016) From suicide enzyme to catalyst: the iron-dependent sulfide transfer in *Methanococcus jannaschii* thiamin thiazole biosynthesis. J. Am. Chem. Soc. 138, 3639–3642 10.1021/jacs.6b00445 26928142 PMC4805478

[15] Sun, J., Sigler, C.L., Beaudoin, G.A.W., Joshi, J., Patterson, J.A., Cho, K.H. et al. (2019) Parts-prospecting for a high-efficiency thiamin thiazole biosynthesis pathway. Plant Physiol. 179, 958–968 10.1104/pp.18.01085 30337452 PMC6393793

[16] Stewart, V. and Parales, Jr. J. (1988) Identification and expression of genes *narL* and *narX* of the *nar* (nitrate reductase) locus in *Escherichia coli* K-12. J. Bacteriol. 170, 1589–1597 10.1128/jb.170.4.1589-1597.1988 2832370 PMC211006

[17] Neidhardt, F.C., Bloch, P.L. and Smith, D.F. (1974) Culture medium for enterobacteria. J. Bacteriol. 119, 736–747 10.1128/jb.119.3.736-747.1974 4604283 PMC245675

[18] Overbeek, R., Begley, T., Butler, R.M., Choudhuri, J.V., Chuang, H.Y., Cohoon, M., et al. (2005) The subsystems approach to genome annotation and its use in the project to annotate 1000 genomes. Nucleic Acids Res. 33, 5691–5702 10.1093/nar/gki866 16214803 PMC1251668

[19] Suzek, B.E., Wang, Y., Huang, H., McGarvey, P.B. and Wu, C.H. (2015) Uniref clusters: a comprehensive and scalable alternative for improving sequence similarity searches. Bioinformatics 31, 926–932 10.1093/bioinformatics/btu739 25398609 PMC4375400

[20] Gerlt, J.A., Bouvier, J.T., Davidson, D.B., Imker, H.J., Sadkhin, B., Slater, D.R. et al. (2015) Enzyme function initiative-enzyme similarity tool (EFI-EST): a web tool for generating protein sequence similarity networks. Biochim. Biophys. Acta 1854, 1019–1037 10.1016/j.bbapap.2015.04.015 25900361 PMC4457552

[21] Datsenko, K.A. and Wanner, B.L. (2000) One-step inactivation of chromosomal genes in *Escherichia coli* K-12 using PCR products. Proc. Natl Acad. Sci. U.S.A. 97, 6640–6645 10.1073/pnas.120163297 10829079 PMC18686

[22] Baba, T., Ara, T., Hasegawa, M., Takai, Y., Okumura, Y., Baba, M. et al. (2006) Construction of *Escherichia coli* K-12 in-frame, single-gene knockout mutants: the Keio collection. Mol. Syst. Biol. 2, 2006.0008 10.1038/msb4100050 PMC1681482 16738554

[23] Guzman, L.M., Belin, D., Carson, M.J. and Beckwith, J. (1995) Tight regulation, modulation, and high-level expression by vectors containing the arabinose P_BAD_ promoter. J. Bacteriol. 177, 4121–4130 10.1128/jb.177.14.4121-4130.1995 7608087 PMC177145

[24] Kabsch, W. (2010) XDS. Acta Crystallogr. D Biol. Crystallogr. 66, 125–132 10.1107/S0907444909047337 20124692 PMC2815665

[25] Afonine, P.V., Grosse-Kunstleve, R.W., Echols, N., Headd, J.J., Moriarty, N.W., Mustyakimov, M. et al. (2012) Towards automated crystallographic structure refinement with phenix.refine. Acta Crystallogr. D Biol. Crystallogr. 68, 352–367 10.1107/S0907444912001308 22505256 PMC3322595

[26] Emsley, P. and Cowtan, K. (2004) Coot: model-building tools for molecular graphics. Acta Crystallogr. D Biol. Crystallogr. 60, 2126–2132 10.1107/S0907444904019158 15572765

[27] Murshudov, G.N., Skubak, P., Lebedev, A.A., Pannu, N.S., Steiner, R.A., Nicholls, R.A. et al. (2011) REFMAC5 for the refinement of macromolecular crystal structures. Acta Crystallogr. D Biol. Crystallogr. 67, 355–367 10.1107/S0907444911001314 21460454 PMC3069751

[28] Moriarty, N.W., Grosse-Kunstleve, R.W. and Adams, P.D. (2009) Electronic ligand builder and optimization workbench (eLBOW): a tool for ligand coordinate and restraint generation. Acta Crystallogr. D Biol. Crystallogr. 65, 1074–1080 10.1107/S0907444909029436 19770504 PMC2748967

[29] Zallot, R., Oberg, N. and Gerlt, J.A. (2019) The EFI web resource for genomic enzymology tools: leveraging protein, genome, and metagenome databases to discover novel enzymes and metabolic pathways. Biochemistry 58, 4169–4182 10.1021/acs.biochem.9b00735 31553576 PMC7057060

[30] Ettema, T.J., Huynen, M.A., de Vos, W.M. and van der Oost, J. (2003) TRASH: a novel metal-binding domain predicted to be involved in heavy-metal sensing, trafficking and resistance. Trends Biochem. Sci. 28, 170–173 10.1016/S0968-0004(03)00037-9 12713899

[31] Monchy, S., Benotmane, M.A., Wattiez, R., van Aelst, S., Auquier, V., Borremans, B. et al. (2006) Transcriptomic and proteomic analyses of the pMOL30-encoded copper resistance in *Cupriavidus metallidurans* strain CH34. Microbiology (Reading) 152, 1765–1776 10.1099/mic.0.28593-0 16735739

[32] Baker-Austin, C., Dopson, M., Wexler, M., Sawers, R.G. and Bond, P.L. (2005) Molecular insight into extreme copper resistance in the extremophilic archaeon ‘*Ferroplasma acidarmanus*’ Fer1. Microbiology (Reading) 151, 2637–2646 10.1099/mic.0.28076-0 16079342

[33] Boyd, E.S. and Barkay, T. (2012) The mercury resistance operon: from an origin in a geothermal environment to an efficient detoxification machine. Front. Microbiol. 3, 349 10.3389/fmicb.2012.00349 23087676 PMC3466566

[34] Szymanski, M.R., Fiebach, A.R., Tratschin, J.D., Gut, M., Ramanujam, V.M., Gottipati, K. et al. (2009) Zinc binding in pestivirus N(pro) is required for interferon regulatory factor 3 interaction and degradation. J. Mol. Biol. 391, 438–449 10.1016/j.jmb.2009.06.040 19540847

[35] Orell, A., Remonsellez, F., Arancibia, R. and Jerez, C.A. (2013) Molecular characterization of copper and cadmium resistance determinants in the biomining thermoacidophilic archaeon *Sulfolobus metallicus*. Archaea 2013, 289236 10.1155/2013/289236 23509422 PMC3595675

[36] Zhang, Y., Rodionov, D.A., Gelfand, M.S. and Gladyshev, V.N. (2009) Comparative genomic analyses of nickel, cobalt and vitamin B_12_ utilization. BMC Genomics 10, 78 10.1186/1471-2164-10-78 19208259 PMC2667541

[37] Hausinger, R.P., Desguin, B., Fellner, M., Rankin, J.A. and Hu, J. (2018) Nickel-pincer nucleotide cofactor. Curr. Opin. Chem. Biol. 47, 18–23 10.1016/j.cbpa.2018.06.019 30015232

[38] Schuelke-Sanchez, A.E., Stone, A.A. and Liptak, M.D. (2020) Cfba promotes insertion of cobalt and nickel into ruffled tetrapyrroles in vitro. Dalton Trans. 49, 1065–1076 10.1039/C9DT03601F 31868194

[39] Guldan, H., Sterner, R. and Babinger, P. (2008) Identification and characterization of a bacterial glycerol-1-phosphate dehydrogenase: Ni^2+^-dependent AraM from *Bacillus subtilis*. Biochemistry 47, 7376–7384 10.1021/bi8005779 18558723

[40] Korshunov, S., Imlay, K.R. and Imlay, J.A. (2016) The cytochrome *bd* oxidase of *Escherichia coli* prevents respiratory inhibition by endogenous and exogenous hydrogen sulfide. Mol. Microbiol. 101, 62–77 10.1111/mmi.13372 26991114 PMC4925259

[41] Hamza, M.A. and Engel, P.C. (2007) Enhancing long-term thermal stability in mesophilic glutamate dehydrogenase from *Clostridium symbiosum* by eliminating cysteine residues. Enzyme Microb. Technol. 41, 706–710 10.1016/j.enzmictec.2007.06.008

[42] Yang, H., Liu, L., Li, J., Du, G. and Chen, J. (2012) Structure-based replacement of methionine residues at the catalytic domains with serine significantly improves the oxidative stability of alkaline amylase from alkaliphilic *Alkalimonas amylolytica*. Biotechnol. Prog. 28, 1271–1277 10.1002/btpr.1611 22887900

[43] Hummel, C.S., Lancaster, K.M. and Crane, III, E.J. (2005) Determination of coenzyme A levels in *Pyrococcus furiosus* and other Archaea: implications for a general role for coenzyme A in thermophiles. FEMS Microbiol. Lett. 252, 229–234 10.1016/j.femsle.2005.09.004 16213671

[44] Rocha, E.R., Tzianabos, A.O. and Smith, C.J. (2007) Thioredoxin reductase is essential for thiol/disulfide redox control and oxidative stress survival of the anaerobe *Bacteroides fragilis*. J. Bacteriol. 189, 8015–8023 10.1128/JB.00714-07 17873045 PMC2168685

[45] Heinemann, J., Hamerly, T., Maaty, W.S., Movahed, N., Steffens, J.D., Reeves, B.D. et al. (2014) Expanding the paradigm of thiol redox in the thermophilic root of life. Biochim. Biophys. Acta 1840, 80–85 10.1016/j.bbagen.2013.08.009 23962628

[46] Herrmann, J.M. and Jakob, U. (2008) Special issue: redox regulation of protein folding. Biochim. Biophys. Acta 1783, 519 10.1016/j.bbamcr.2008.03.002 18395585 PMC2794659

[47] Leary, S.C. (2010) Redox regulation of SCO protein function: controlling copper at a mitochondrial crossroad. Antioxid. Redox Signal. 13, 1403–1416 10.1089/ars.2010.3116 20136502

[48] Vetriani, C., Speck, M.D., Ellor, S.V., Lutz, R.A. and Starovoytov, V. (2004) *Thermovibrio ammonificans* sp. nov., a thermophilic, chemolithotrophic, nitrate-ammonifying bacterium from deep-sea hydrothermal vents. Int. J. Syst. Evol. Microbiol. 54, 175–181 10.1099/ijs.0.02781-0 14742477

[49] Giovannelli, D., Sievert, S.M., Hügler, M., Markert, S., Becher, D., Schweder, T. et al. (2017) Insight into the evolution of microbial metabolism from the deep-branching bacterium, *Thermovibrio ammonificans*. eLife 6, e18990 10.7554/eLife.18990 28436819 PMC5441870

[50] Engilberge, S., Wagner, T., Santoni, G., Breyton, C., Shima, S., Franzetti, B. et al. (2019) Protein crystal structure determination with the crystallophore, a nucleating and phasing agent. J. Appl. Crystallogr. 52, 722–731 10.1107/S1600576719006381 31396026 PMC6662991

[51] Stewart, L.C., Algar, C.K., Fortunato, C.S., Larson, B.I., Vallino, J.J., Huber, J.A. et al. (2019) Fluid geochemistry, local hydrology, and metabolic activity define methanogen community size and composition in deep-sea hydrothermal vents. ISME J. 13, 1711–1721 10.1038/s41396-019-0382-3 30842565 PMC6776001

[52] Pimentel, C., Caetano, S.M., Menezes, R., Figueira, I., Santos, C.N., Ferreira, R.B. et al. (2014) Yap1 mediates tolerance to cobalt toxicity in the yeast *Saccharomyces cerevisiae*. Biochim. Biophys. Acta 1840, 1977–1986 10.1016/j.bbagen.2014.01.032 24486411

[53] Milne, N., Luttik, M.A.H., Cueto Rojas, H.F., Wahl, A., van Maris, A.J.A., Pronk, J.T. et al. (2015) Functional expression of a heterologous nickel-dependent, ATP-independent urease in *Saccharomyces cerevisiae*. Metab. Eng. 30, 130–140 10.1016/j.ymben.2015.05.003 26037463

[54] Kalms, J., Schmidt, A., Frielingsdorf, S., van der Linden, P., von Stetten, D., Lenz, O. et al. (2016) Krypton derivatization of an O_2_-tolerant membrane-bound [NiFe] hydrogenase reveals a hydrophobic tunnel network for gas transport. Angew. Chem. Int. Ed. Engl. 55, 5586–5590 10.1002/anie.201508976 26913499

[55] Kalms, J., Schmidt, A., Frielingsdorf, S., Utesch, T., Gotthard, G., von Stetten, D. et al. (2018) Tracking the route of molecular oxygen in O_2_-tolerant membrane-bound [NiFe] hydrogenase. Proc. Natl Acad. Sci. U.S.A. 115, E2229–E2237 10.1073/pnas.1712267115 29463722 PMC5877991

[56] Alfano, M. and Cavazza, C. (2020) Structure, function, and biosynthesis of nickel-dependent enzymes. Protein Sci. 29, 1071–1089 10.1002/pro.3836 32022353 PMC7184782

[57] Kobayashi, M. and Shimizu, S. (1999) Cobalt proteins. Eur. J. Biochem. 261, 1–9 10.1046/j.1432-1327.1999.00186.x 10103026

[58] Fukuzumi, S., Cho, K.B., Lee, Y.M., Hong, S. and Nam, W. (2020) Mechanistic dichotomies in redox reactions of mononuclear metal-oxygen intermediates. Chem. Soc. Rev. 49, 8988–9027 10.1039/D0CS01251C 33316016

[59] Wang, Y., Li, J. and Liu, A. (2017) Oxygen activation by mononuclear nonheme iron dioxygenases involved in the degradation of aromatics. J. Biol. Inorg. Chem. 22, 395–405 10.1007/s00775-017-1436-5 28084551 PMC5360381

[60] Fiedler, A.T. and Fischer, A.A. (2017) Oxygen activation by mononuclear Mn, Co, and Ni centers in biology and synthetic complexes. J. Biol. Inorg. Chem. 22, 407–424 10.1007/s00775-016-1402-7 27853875

[61] Shearer, J., Peck, K.L., Schmitt, J.C. and Neupane, K.P. (2014) Cysteinate protonation and water hydrogen bonding at the active-site of a nickel superoxide dismutase metallopeptide-based mimic: implications for the mechanism of superoxide reduction. J. Am. Chem. Soc. 136, 16009–16022 10.1021/ja5079514 25322331

[62] García-García, J.D., Joshi, J., Patterson, J.A., Trujillo-Rodriguez, L., Reisch, C.R., Javanpour, A.A. et al. (2020) Potential for applying continuous directed evolution to plant enzymes: an exploratory study. Life (Basel) 10, 179 10.3390/life10090179 PMC7555113 32899502

[63] Rix, G. and Liu, C.C. (2021) Systems for in vivo hypermutation: a quest for scale and depth in directed evolution. Curr. Opin. Chem. Biol. 64, 20–26 10.1016/j.cbpa.2021.02.008 33784581 PMC8464631

