# Supplementary material for: Structure and function of aerotolerant, multiple-turnover THI4 thiazole synthases
Source: Biochem J. 2021 Sep 7;478(17):3265–79. doi: 10.1042/BCJ20210565 (PMC8454699; doi:10.1042/BCJ20210565)
Supplement: Supplementary Figures S1-S6 and Tables S2-S4 [file BCJ-478-3265-s1.pdf]

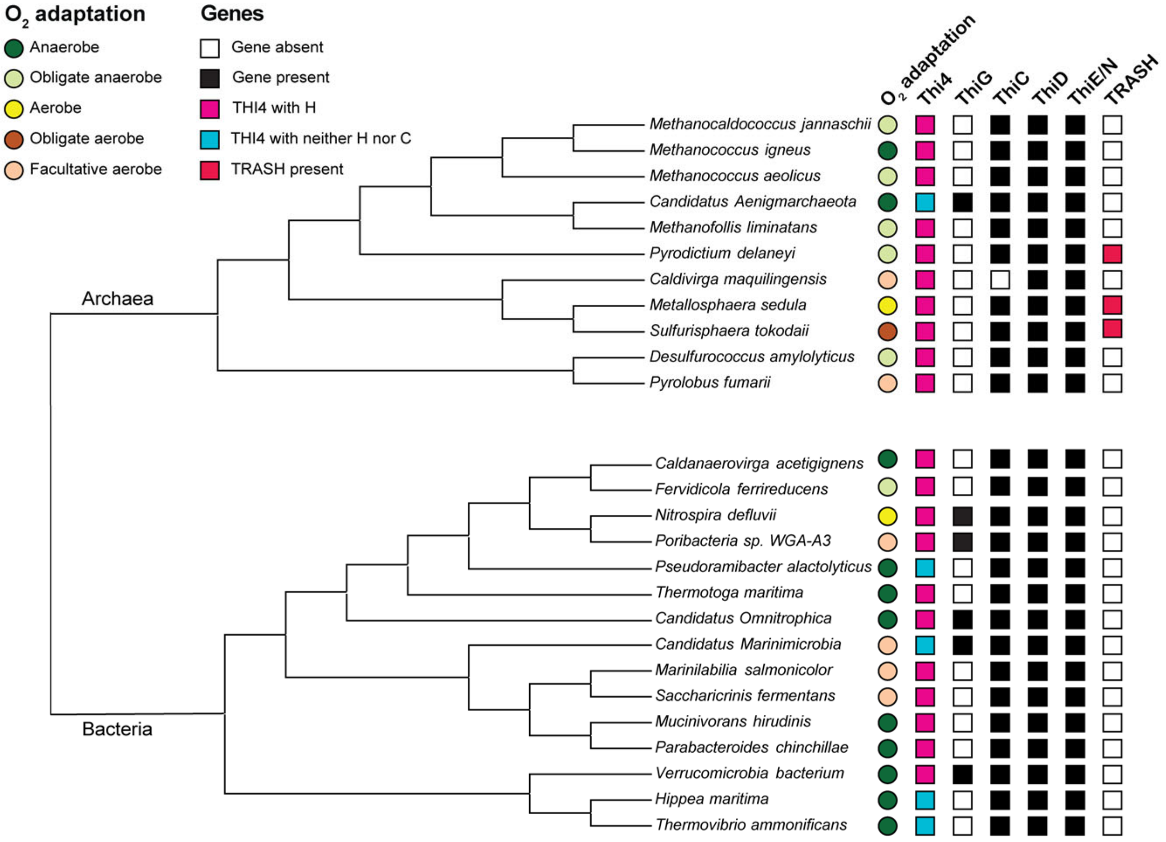

Supplementary Figure 1. Ecology and genomic context of the 26 THI4s selected for testing.

The first column (colored bullets) shows the O_2 adaptation of the 15 bacteria and 11 archaea whose THI4s were tested. The second column (colored squares) shows which residue replaces the active-site cysteine in each THI4. The next four columns (black or white squares) indicate the presence or absence of other thiamin synthesis enzymes (ThiG, ThiC, ThiD, and ThiE or ThiN). The last column (red or white squares) indicates presence or absence of a gene encoding a protein from the TRASH family (Trafficking, Resistance, And Sensing of Heavy metals) that is clustered with the THI4 gene.

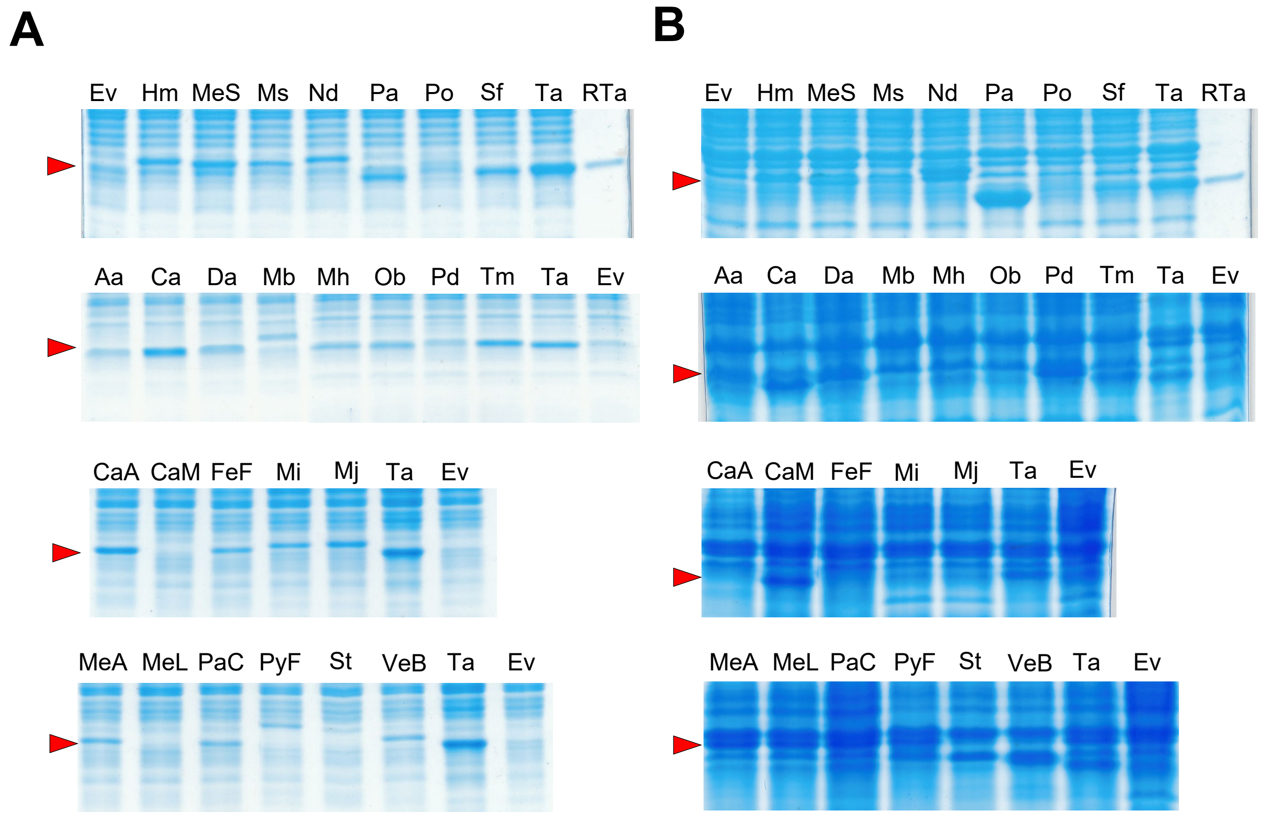

Supplementary Figure 2. Soluble expression of non-Cys THI4s.

Quantitative gel analysis of (**A**) soluble and (**B**) insoluble expression in *E.coli* of 26 selected non-Cys THI4s. Soluble and insoluble fractions of cells were run on 15% gels, stained with Coomassie blue, and scanned to quantify the THI4 band, for which purified recombinant *Thermovibrio ammonificans* THI4 (RTa, arrow) served as a marker. Organism abbreviations are as in Table 1.

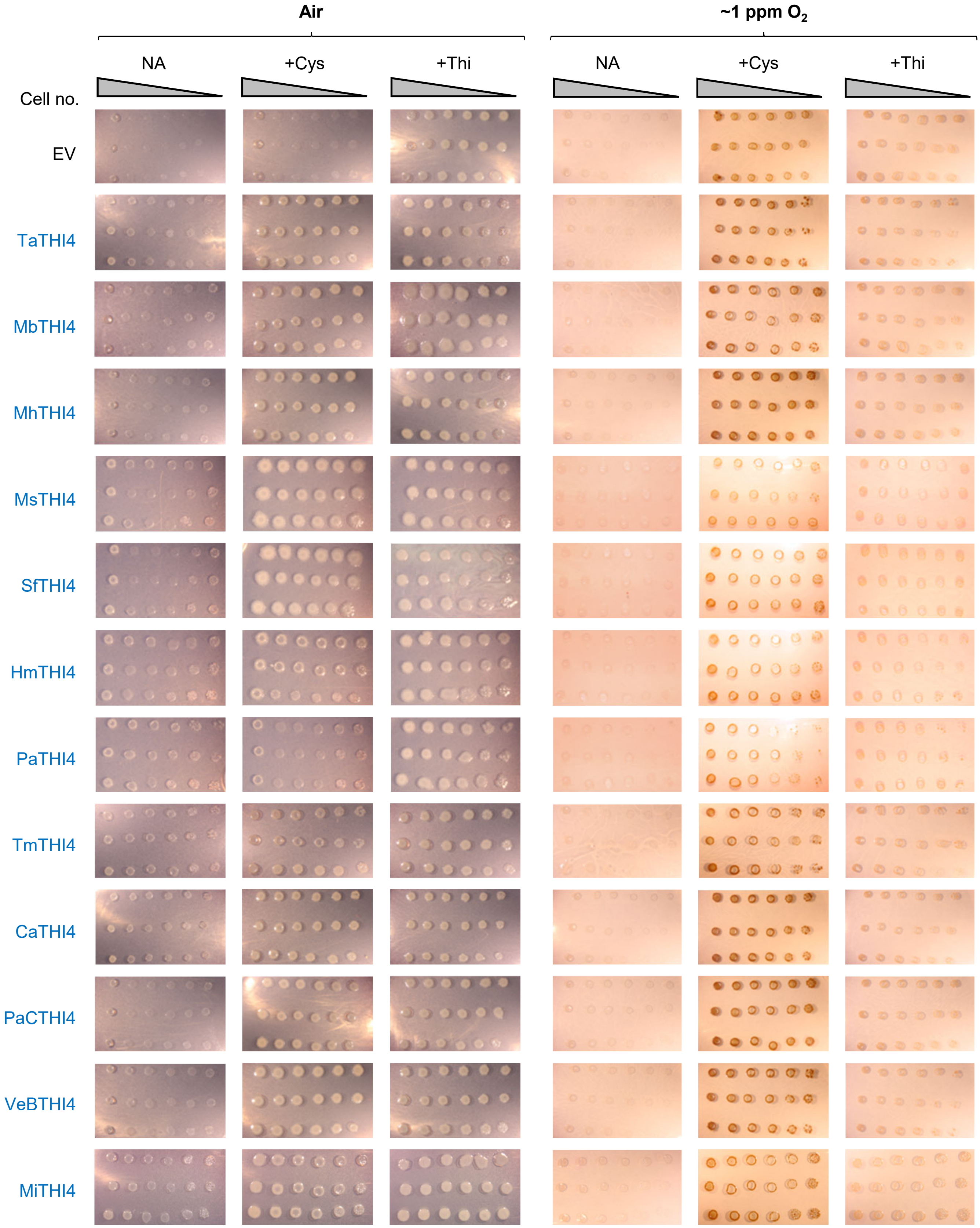

Air
~1 ppm O_2
NA
+Cys
+Thi
NA
+Cys
+Thi
Cell no.
EV
TaTHI4
MbTHI4
MhTHI4
MsTHI4
SfTHI4
HmTHI4
PaTHI4
TmTHI4
CaTHI4
PaCTHI4
VeBTHI4
MiTHI4

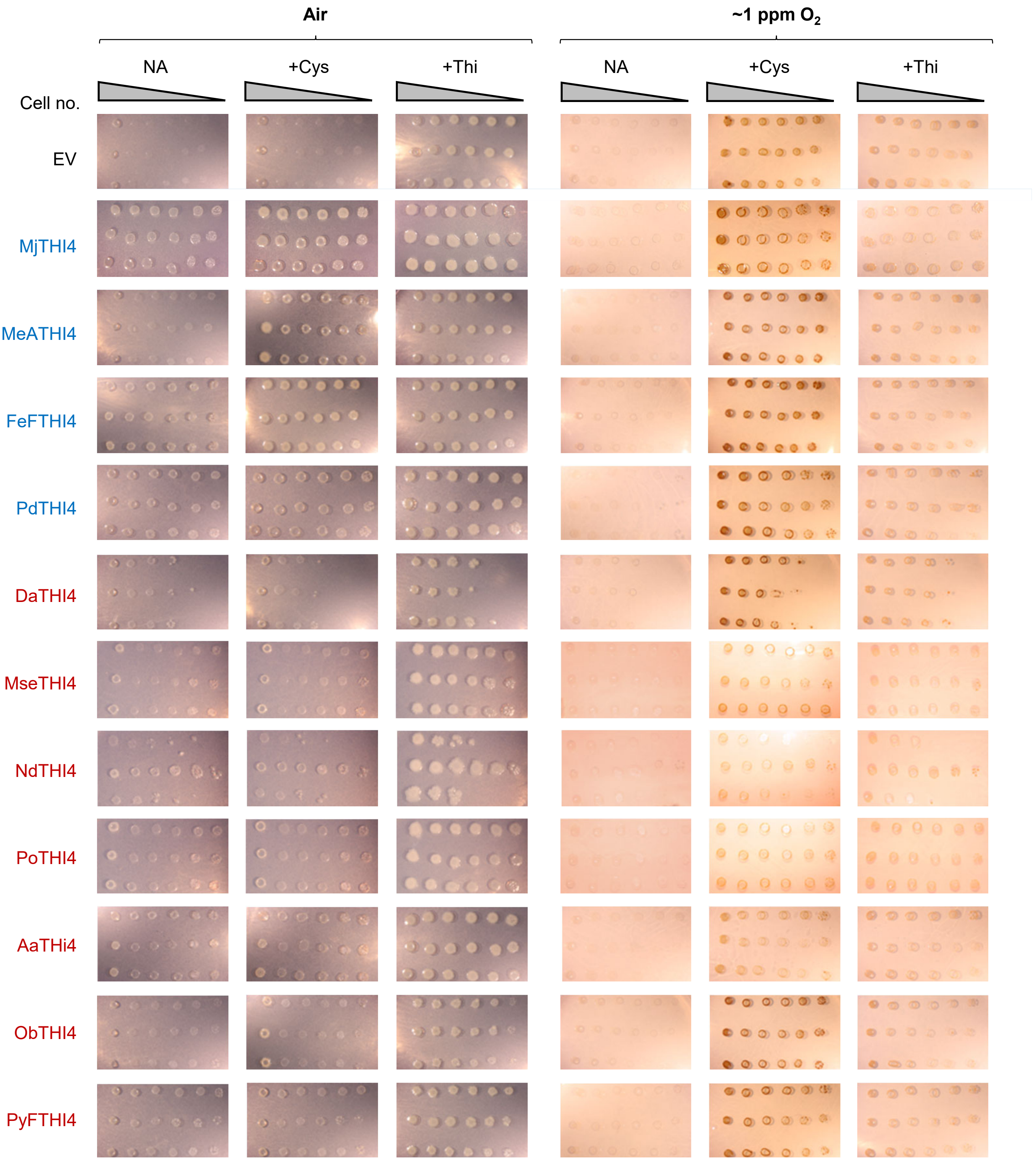

Supplementary Figure 3. Functional complementation tests of non-Cys THI4s.

Tests of functional complementation of an *E. coli* Δ*thiG* strain by all 23 soluble non-Cys THI4s or the empty vector (EV). Organism abbreviations are as in Table 1. Overnight cultures of three independent clones per construct were 10-fold serially diluted and spotted on plates of MOPS minimal medium containing 0.2% glycerol and 0.02% arabinose with no additions (NA) or plus 1 mM Cys or 100 nM thiamin. Cells were cultured in air or ~1 ppm O_2. The medium used for culture in ~1 ppm O_2 contained 40 mM nitrate. Images were captured after incubation at 37°C for 7 d. The high background in the ~1 ppm O_2 +Cys treatment is staining of the inoculum cells. Organisms whose THI4s showed clear complementing activity in air, particularly with Cys supplementation, are blue; organisms whose THI4s did not show such activity are red. Note that complementing activity was scored from direct visual inspection of plates, not from the above images, which do not fully capture growth in every case.

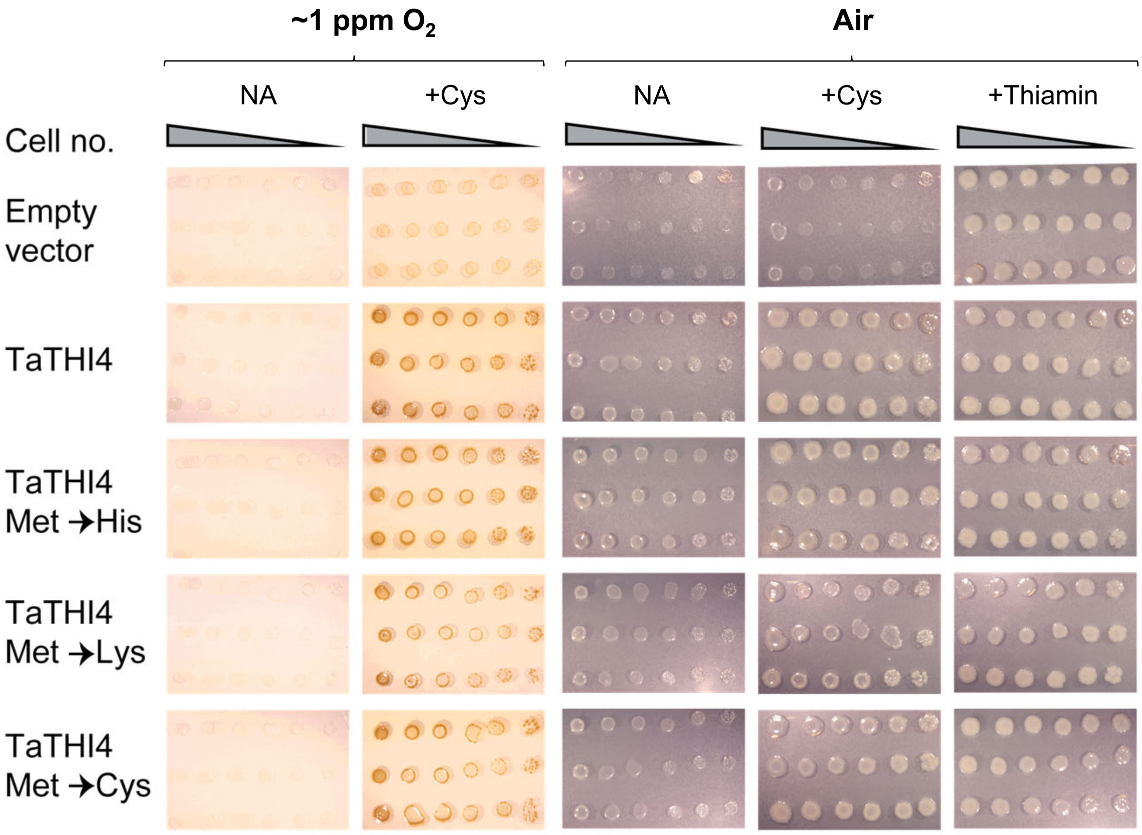

Supplementary Figure 4. Complementation activity of TaTHI4 mutants

An *E. coli* Δ*thiG* strain was transformed with empty vector or vector harboring wild type TaTHI4 or TaTHI4 with the indicated mutations of Met158. Overnight cultures of three independent clones per construct were 10-fold serially diluted and spotted on plates of MOPS minimal medium containing 0.2% (w/v) glycerol and 0.02% (w/v) arabinose with no addition (NA) or with1 mM Cys or 100 nM thiamin. The medium used for culture in ~1 ppm O_2 contained 40 mM nitrate. Cells were cultured in air or under N_2 containing ~1 ppm O_2. Images were captured after incubation at 37°C for 7 d.

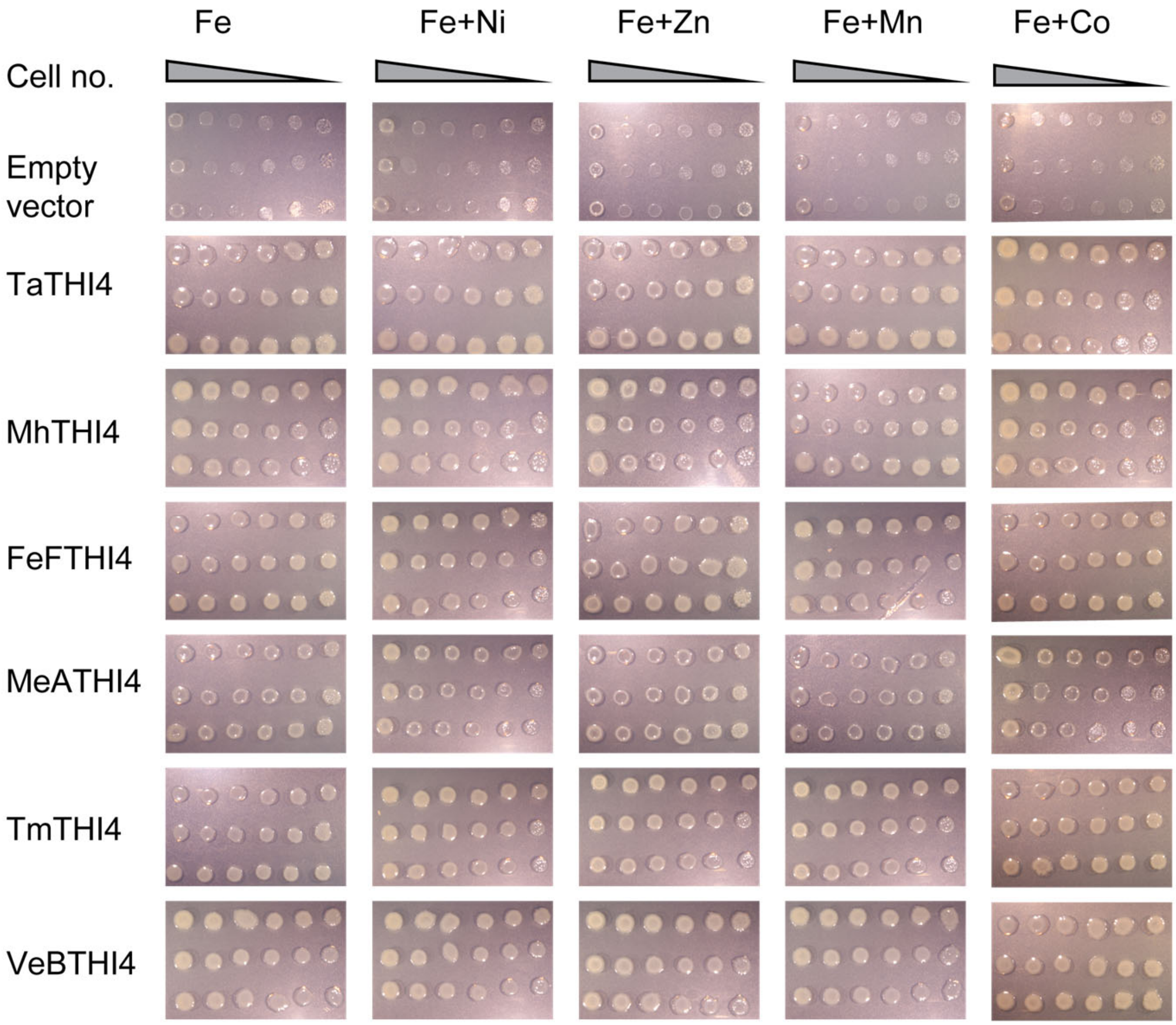

Supplementary Figure 5 Effect of metal supplementation on complementing activity of THI4s

An *E. coli* Δ*thiG* strain was transformed with empty vector or vector harboring the indicated THI4 sequence. Overnight cultures of three independent clones per construct were 10-fold serially diluted and spotted on plates of MOPS minimal medium containing 0.2% (w/v) glycerol, 0.02% (w/v) arabinose, 1 mM Cys, and 100 µM of the indicated metal. All media also contained the standard concentration of ferrous iron (100 µM). Cultures were incubated in air at 37°C for 7 d.

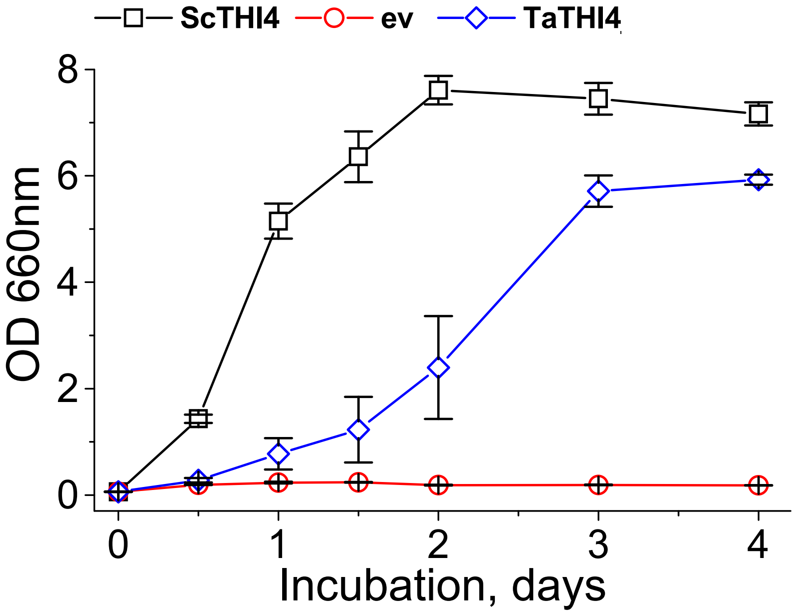

Supplementary Figure 6 Complementation of a yeast Δ*THI4* strain by TaTHI4

Cells of the Δ*THI4* strain transformed with empty vector (ev) or vector harboring TaTHI4 or yeast THI4 (ScTHI4) as positive control were cultured in thiamin-free SC minus histidine medium. Data are means of three or four independent clones ± S.E.

Supplementary Table 2 ***T. ammonificans*** **THI4 data collection and refinement statistics**

	TaThi4
**Data collection**	
Space group	I121
Cell dimensions	
a, *b*, *c* (Å)	89.89, 89.69, 131.81
α, β, γ (°)	90, 96.98, 90
Resolution (Å)	43.63-2.30 (2.36-2.30)*
R_{merge}	0.092 (1.552)
$I / \sigma I$	8.23 (0.77)
Completeness (%)	99.46 (96.62)
Redundancy	2.0 (2.0)
**Refinement**	
Resolution (Å)	43.63-2.30
No. reflections	93685
R_{work} / R_{free}	0.217/0.275
No. atoms	
Protein	7835
Ligand/ion	160
Water	58
B-factors	
Protein	50.69
Ligand/ion	41.32
Water	47.34
R.m.s. deviations	
Bond lengths (Å)	0.009
Bond angles (°)	1.12

*Values in parentheses are for highest-resolution shell.

Supplementary Table 3 Sequences of 199 non-Cys THI4s from SEED and UniRef90 databases

The 26 sequences selected for testing are boxed. Those that had little to no complementing activity are in red font. Those that were poorly expressed in soluble form in *E. coli* and were not tested are in gray font. The residue that replaces Cys in the active site is highlighted in cyan. Met and non-active-site Cys residues in each sequence are highlighted in yellow; the number per sequence is given in the header. Mean values were: Active THI4s: 10.6 Met, 2.3 Cys. Inactive THI4s: 9.1 Met, 3.1 Cys.

```
>Caldanaerovirga acetigignens (2 Cys, 11 Met)
MKSFSIPVPDTKVSSLIMKHYFKDLEDAVKSDVIVAGAGPSGLTCAWTLADQGYKVTVLDRRLAPGGGIWGGAMSFNKVVLQKDVEWILKEADVPFV
EDEGALVVSAPLFASKLIAKAAAHPGIRFFNMLTVVDLHSSGDRITGVVVNNSAIEMAGLHVDPMVLTAKAVLDATGHDAVLANLYSRRAGTGLIRE
SFMNAEKGEEDVVANTRMLAPGLFVAGMAANNVEGGCRMGPIFGGMLLSGKKAARLIINYLSENSK

>Candidatus Marinimicrobia bacterium (4 Cys, 12 Met)
MEKIVSFGIIDSYQKKLKENLEVDVAIVGGGPSGLIAAKYLAQAGKKVVLFERKLAPGGGMWGGAMMFNQIVVQEDAISILEDVDISYNLYEEGYYV
CDSVEATAALIFSAKKAGATIFNCFSVEDVVFQRGSVAGVVVNWASVHREGMYVDPLVIMAKAVLDSTGHSCEVASILARKNEVKLMTRTGNIMGER
SLSIEEGELTTIENTKEIFPGLYVSGMAANAVSGSFRMGPIFGGMLMSGKKVAGLINDRLSKRDGCK

>Fervidicola ferrireducens (2 Cys, 11 Met)
MKSFSVPVPDTKVSSLIMKHYFKDLEDAVKSDVIVAGAGPSGLTCAWTLADQGYKVTVLDRRLAPGGGIWGGAMSFNKVVLQKDVEWILKEADVPFV
EDEGALVVSAPLFASKLIAKAAAHPGIRFFNMLTVVDLHSTGDKITGVVVNNSAIEMAGLHVDPMVLTAKAVLDATGHDAVLANLYSRRAGTGLIRE
SFMNAEKGEEDVVANTRMLAPGLFVAGMAANNVEGGCRMGPIFGGMLLSGKKAARLIINYLSENSK

>Hippea maritima strain ATCC 700847 (2 Cys, 9 Met)
MNNLDERVISRAIVERYMNKLLDYLECDVTIVGGGPAGLVCAYYLAKANIKVAIFDKRLTIGGGMWGGAMLFNEIVVQEIGREILDEFGINYEKYTD
GYYTADSIEATTTLISKTVKAGAKIFNAIEVEDVVFKKIDGQYRVNGLVVGWTTVNMAGLLVDPLVVTSKYVIDATGHDADIANILTRKGGIKLNTP
EGVVIGEKPMWAEVGEQSTIEETQEVYPGLIVAGMAAVAVSGSHRMGPVFGGMLNSGKKAAQIVIESLKK

>Marinilabilia salmonicolor (2 Cys, 12 Met)
MEQIVSSGIIDSYFSKLKENLAVDVAIVGGGPSGLIAAYYLAKTGKKVALFERKLAPGGGMWGGAMMFNEIMVQKEALHILKELGIEYKHYRDDYYT
VDSVHATSALTYHATKAGARIFNCTSIEDVVFHNNIVSGLVINWAPVHREGMHVDPLIIMAKAVIDGTGHDCEIVHTVARKNDIKIDTPSGKVMGER
SLAVEEAERTTVDNTKEVFPGLFVSGMAANGTSGSYRMGPIFGGMLLSGQKVAGIISEKLAKEAIMESANN

>Methanocaldococcus jannaschii DSM 2661 (= Methanococcus jannaschii) (3 Cys, 10 Met)
MVNLMNIKDIKLNADETKTTKAILKASFDMWLDIVEADVVIVGAGPSGLTCARYLAKEGFKVVVLERHLAFGGGTWGGGMGFPYIVVEEPADELLRE
VGIKLIDMGDGYYVADSVEVPAKLAVAAMDAGAKILTGIVVEDLILREDGVAGVVINSYAIERAGLHIDPLTIRSKVVVDATGHEASIVNILVKKNK
LEADVPGEKSMWAEKGENALLRNTREVYPNLFVCGMAANASHGGYRMGAIFGGMYLSGKLCAELITEKLKNKE

>Methanotorris igneus (= Methanococcus igneus) (2 Cys, 8 Met)
MDVRLRADEYATTRAILKSAFDMWLDIIDVDVAIVGGGPSGLTAARYIAKEGYKVVVLERHLAFGGGTWGGGMGFPYIVVEEPADEILREVGVKLEK
VEGEDGLYTADSVEVPAKLAVGAIDAGAKVLTGIVVEDLVLRENRVAGVVINSYAIEKAGLHIDPITITAKYVVDATGHDASVVTTLSRKNPELGLE
VPGEKSMWAEKGENALLRNTREVYPGLFVCGMAANAVYAGHRMGAIFGGMYISGKKCAEMIVEKLKNNE

>Methanococcus aeolicus strain ATCC BAA-1280 (3 Cys, 8 Met)
MDISKIDLKADEKAVTKSIFKATYEMWMDNLEVDVVIVGGGPSGLTAGRYLADAGVKVLILERHLSFGGGTWGGGMGCPYITVQSPADEILSEVGIK
LEEGEDGLFVADSVEVPAKLGTGAIDAGAKVLTGIVVEDVILKEGKVSGVVINSYAINKAGLHIDPLTINAKYVIDATGHDASVACTLARKNEDLGL
VIPGEKSLWADEGENGLLKYTKELFPGLFVCGMASNATHGGYRMGAVFGGMYISGKIVADMILEKLKNE

>Mucinivorans hirudinis (3 Cys, 10 Met)
MEKIVSAGIVESYFDKLRRNLVLDVAIVGGGPSGLVAAYYLAKAGRRVALFERKLAPGGGMWGGAMMFNDIVVQSDALPILEELGVSYRHYRGDAYL
VDSVHATAALIYAATRAGATIFNCYSVEDVVFKDERVAGLVVNWAPVIREGMHVDPLVIMATAVLEGTGHDCAIARLVARKNGVRLNTPTGEVIGER
SLSIEEAERTTVENTKEIYPGLFVSGMAANGVSGSFRMGPIFGGMLLSGKKAAQMICDSL

>Parabacteroides chinchillae (3 Cys, 11 Met)
MEQIVSTGIIDSYFAKLKSNLSVDVAIVGGGPSGIVAAYYLAKAGKKVALFDRKLAPGGGMWGGAMMFNDIVVQEEAMPIVKELGVSYHAAGNGTYI
MDSVHTTSALIYQATKAGATIFNCYSVEDVVFHNDAVAGVVVNWAPVIREGMHVDPLTIMAKAVLEGTGHDCEVARTVARKNDIKLNTPTGGVIGER
SLNVELGESTTVENTKEIYPGLFVSGMAANGVSGSFRMGPIFGGMLMSGKKAAELICDKLGK

>Pseudoramibacter alactolyticus ATCC 23263 (2 Cys, 19 Met)
MLSDTKISEAILTTYTDRFKQMLSSDAVIVGGGPSGLIAAYYLGKAGVKTTLLDRRLSVGGGMWGGGMMMNQIVVQKSVLPILEEMGIACKAYDAEH
YTVSSVACISGLIFRAAQSGATIMNLVTMEDAVVREGRLEGLVINWSTVEMAHLMVDPLMMDARVVLDATGHDAALVTKLVERMGPVLNTPSGGLEG
EKPMWADHGEKQVVANTREVYPGLYVSGMAANATFGGQRMGPVFGGMLLSGKKAAEAMLRRLAQ

>Pyrodictium delaneyi (0 Cys, 10 Met)
MGIASFYPGELEKQYSEAKLARIALKVALEKLSYAVEADVAIAGAGPAGLTLAWLLAEQGLRVTLVEHRLSTGGGMKGGSMLFPVALVEEGLAAVIL
EKAGVRLHRVGEGLYAMDPVEAVAKLTARAVDAGAVILPGLHVEDLIVRGSGSNVRVAGIVVNWAPVVEAGWHVDPLYIEARAVVDATGHDAQLARL
LERRLPGSLKVPGMSSLDVWTGERQVVEHTGEIFPGLYAAGMSVAEVYNLRRMGPVFGGMIASAARLAEMLAERLAGKRMGLATGVARSG
```

```
>Saccharicrinis fermentans DSM 9555 (2 Cys, 10 Met)
MEQIVSVGIVDSYFKKLKENLTVDVAIVGGGPSGMVAAYYLARQGFKVSVYERKLAPGGGMWGGAMMFNEIVIQKEALPILDELNISYKHYDKDYYT
LDSVHATSALIYHATQAGATFFNCTSVEDVVFLDNKVSGVVLNWAPVHREKMHVDPLVIMAKAVIDGTGHDCDIARILERKNNIQLLTASGKVEGER
SLSIDEAERTTIENTKEIYPGLYVSGMASNGVSGGFRMGPIFGGMLLSGKKVANLIADNLNK

>Thermotoga maritima strain ATCC 43589 (2 Cys, 8 Met)
MRDVLISRLIVERYFEKLRNSLELDVAIVGAGPSGLTAAYELAKNGFRVAVFEERNTPGGGIWGGGMMFNEIVLEKELENFLKEVEIEYEVKEDHIV
VDSVHFASGLLYRATKAGAIVFNNVSVEDVAVQNGRVCGVVVNWGPTVRLGLHVDPITVKASFVVDGTGHPANVVSLLAKRGLVEMKTEFPMDADEA
EKFVVDNTGEIFPGLLVSGMAVCAVHGGPRMGPIFGGMILSGQKVARIVSERLR

>Thermovibrio ammonificans strain DSM 15698 (3 Cys, 10 Met)
MQNLSEVVISEAIITAFMEKLKSHLETDVAIVGGGPSGLVAGYYLAKKGYRVAIFERRLSIGGGMWAGAMFFNEIVVQEMGREILDEFGVNYREFKP
GYYLADAVEATTTIASKAVKAGATVFNGVTAEDVVLKQVNGQYRVCGLVINWTTVELNHLMVDPLVITAKYVVDATGHDASVVSTLQRKAGIKLNTE
TGCVVGEKPLWASVGEEDTVKNSKEVFPGIYVSGMAANATCGSHRMGPVFGGMLMSGKKVAEEIAAKLNQNKEA

>Verrucomicrobia bacterium (1 Cys, 11 Met)
MLNEVTISRAIIDAYFKKLTRHLEVDVAIVGGGPSGLVAGHDLARAGKKVALFESKLAIGGGIWGGGMGFNEIVVQEAAREMLVEFGLRATEFEPGY
YTLDAVHVAAALAARAMEAGLTVFNLTSMEDVVIQKDRVAGLVLNWTAIRHLKWHVDPLTIHSRFVLDATGHPASVAETLVRKMNVRLDTTTGGLVG
EKPMAAEDGERQTVENTREVYPGLFVSGMAAITVCGGHRMGPVFGGMLLSGRKAAQAMLAELGT

>Candidatus Omnitrophica bacterium 4484_171 (8 Cys, 9 Met)
MLEETIISKAIIDSYHNKLSSIIDVDAAICGGGPSGLVCAASLAAAGKKVVLFEKKLSLGGGMWGGGMMFNEIVVQKKAKKILDEFSVRTKKYKENY
YLADSSECVCALGYNAVHSGAVIINGVFAEDVMVKKNRICGLVINWSAAASANLHVDPLTVRAKFVVDATGHPSEVVKVVEKKSGVKIKTKTGKVLG
EKSMWAHAAENTIEKNTRQIAPGLFVTGMCANAVCGAPRMGPIFGGMLLSGKKCAKIILSRL

>Poribacteria sp. WGA-A3 (5 Cys, 10 Met)
MDNLQPAPLRERDVTRHIAREFYKEFDQLIESDVIIVGGGPSGLVCAHDLATQGFRTLLIEQSLALGGGFWSGGYLMNKATLCEPAHSILENMGVPC
KPVKDCAGMRIVDPPHATARLIASAYEAGAKVLNLTRVVDLILHGEGVLEGVVVNNTTAEMAGHDMIHVDPIALESRVVVDATGHDAVVVGLLNQRG
LYATVPGNGAMWVARSEAMVVDNTREVFPNCFVTGLAVAAVDGSPRMGPAFGSMLLSGRRAADLVRHKLKGE

>Nitrospira defluvii (6 Cys, 12 Met)
MEELARSRKACSTAVEGEYRMGKPKPAPLRERDITRQIAREYYKEFDQLIESDVIIVGAGPSGLICAHDLGRMGIKTLIVEQSLALGGGFWSGGYLM
NKATICAPAHKILKEVGVPCKQIKECPGMYMVDPPHATGALIAAAYNAGAKIMNLTRVVDLILRREGVLEGVVVNSTTAEMAGHDIIHVDPIALESK
IVVDATGHDAVVVNLLHKRGLYQQVPGNGAMWVSRSEEEVMDRTGEVSPNCFVIGLAVAAVFGTPRMGPAFGSMLLSGRYGAELIRDKLKNR

>Desulfurococcus amylolyticus strain DSM 18924 (1 Cys, 5 Met)
MSLESHITRVIWEEASRDWVELSSCDIVVVGAGPSGLTAAKYLAEKGLKTLVLERRLSFGGGIGGGGMLLHKTVVDERGLGILRDFNIRYKPSSIKG
LYVVDTAELTAKLAAGALDAGAKIIPGISVEDVIVRYNPFRVQGVVVEWSAVQLSGLHVDPLFIESKAVIDATGHDAEVLRILEKKNPESKVKIPGE
KSAYSEKADVDVVEYTGRVIPGLYATGMAVAAVRGLNRMGPIFTGMLLSGRKVAEAVIRDLESAPK

>Candidatus Aenigmarchaeota archaeon (2 Cys, 10 Met)
MGEIFSKVSEKEVTSAIVSGFIKEFEKIIESDVIIVGGGPSGLMAGKELSSKGKKVVIIERNNYLGGGFWTGGYLMNKITVRHPGEEILKDLGIPFE
EFGEGLYLADGPHACSKLIAATCDAGVKILNMTTLEDVVLKEKGAVGGVVINWTPIETLPREIASVDPIALESKVVIDATGHDANVVKKIEERGILK
TKGYGAMWVEKSEDMVVKYTGEVHPGLVVTGMAVSTFFGLPRMGPTFGAMLLSGKKAAEVTMEILSR

>Pyrolobus fumarii strain DSM 11204 (0 Cys, 10 Met)
MVIPGHMTTRRTAMPGLDAIITRVIIEEASKELVEYAESDVIVVGAGPAGLTAAFYLAKRGFRVLVLERRLSVGGGIGGGGMLFHKVLVQEEALPVL
NDMGIRVHPTSVKGIYSLDSVALITGLASAAVNAGAKIILGLEAVDLVVRKEGERHRVAGVMALWSAVGIANLHVDPLMFEAKAVVDATGHDARLAR
IAHQKLRGEAPEVPGDGPAWAEEGEKLVVKATGELIPGLYVAGMAATAVKGYYRMGPIFGGMLLSGKKVADLITEKLRGK

>Metallosphaera sedula strain ATCC 51363 (0 Cys, 8 Met)
MNIKQVDEIKITRYILKATFEDWMDFSVNDVVIVGAGPSGLAAAYYSAKAGLKTTVFERRLSFGGGIGGGAMLFHKIVIESPADEILREIGVKLQKF
EEGVYVVDSSEFMAKLAAATIDAGAKIIHGVTVDDVIFRENPLRVTGVAVEWTATQMASLHVDPLFISAKAVVDATGHDAEVISVASRKIPELGIVI
PGEKSAYSEIAEQLTVEQSGEVAPGLYAAGMAVTEIKAIPRMGPIFGAMLLSGKKVAEDIIKNLQANSATLKSVQKE

>Caldivirga maquilingensis strain ATCC 700844 (0 Cys, 10 Met)
MAGISIREASITRAIVNSALKLLSEYSSVDVAIVGAGPSGMTAAYYLAKAGLKTLVLERRFSFGGGIGGAASHLPSIIVEHPVSEILSKDFGIKIMD
MGDGLFTVDPAEMIAKLAVKAIDAGAKFLLGVHVDDVIYRDNPPRITGLALYWATIQMAGVHTDPFFIESNAVVDATGHDAEVAAVASRKIPELGIV
VRGEKSAYVGVAEDLVVKYTGKVIDGLYVTGMAVAAVHGLPRMGPIFGSMIMSGKRVAEIIIEDLKGNH

>Methanofollis liminatans DSM 4140 (5 Cys, 12 Met)
MELDEVTISRAILATQMETMVEYLDLDVAVVGGGPSGITCAALLAEKGVKVGLFEKKLSIGGGMWGGGMMFPRIVVQAEAKRILDRFGIASKEFEPG
YHVAKSVEAVSKLTAAACTAGAEFFNLIAVEDVVIKGDGRLAGLVVNWSPVEMAGLHIDPLTIRCKAVVDASGHDATIAHMVAKKGGDLPIRGEGFM
WADRAEGNILEHTREVFPGLFVCGMAANAVAGECRMGPIFGGMLLSGERAADLAAAAVLHP

>Sulfurisphaera tokodaii strain DSM 16993 (0 Cys, 10 Met)
MDSNSIKVKQVDEVKISKYILKYTFQDWEDIVESDVVIVGAGPSGMTAAYYLAKAGLKTVVFERRLSFGGGIGGGAMLFHKIVIESPADEILKEMKI
KLNKVEEGVYIVDSAEFMAKLAASAIDAGAKIIHGVTVDDVIFRENPLKVVGVAVEWTATQMAGLHVDPLFISAKAVVDATGHDAEVISVAARKIPE
LNIVIPGEKSAYSETAEELTVENTGMVAPGLYAAGMAVTEVKGLPRMGPIFGAMVLSGKRVAEIIIKDLRYS
```

>Deltaproteobacteria bacterium HGW-Deltaproteobacteria-1
MVLDEIVISKAIIERFLEKLLQATDVDVAIVGGGPSGLVAAYYLASAGKKVALFERKLSLGGGMWGGGMMFNEIVVQDEAREILDVFDIRYREYQQG
YYTADAVLAVTSICSQAARAGASIFNCVSVEDVMIREGRVTGLVINWSPVEMAGLHVDPLTIAAGSVIDTTGHATEVLKVIERKADMQLATPSGKLV
GERSMWAEKAERLTMDNTRQICPGVYVAGMSANAAFGGPRMGPIFGGMLLSGRKVAELILASS

>Thermogladius calderae (strain DSM 22663 / VKM B-2946 / 1633)
MELESIITRLVVEESARELVELSESDVLVVGAGPSGLTAAKYLADKHLKVVVLEKRLSYGGGIGGGGSLFHKVVVDERALPVLGDFKVRYKAAGVAG
YYVVDSAELMSKLAAGALDSGAKIILGAEVEDLVVRDNPLRVVGVMFKWSAITAAGLHVDPLFALSRAVVDATGHEAVLVSILSRKNRVAGVAVPGE
RSGFAERAERDVVEYTGRMVPGLYVAGMSVAAVHGLHRMGPIFTGMLLSGRKVAEAIARDLGVPQ

>Acetomicrobium thermoterrenum DSM 13490
MKLDELVITKAIVEGYFKKLMNCLELDVAIVGGGPSGLVAALELAKAGKKVALYERKLSVGGGMWGGGMLFNEIVIQHEAKEILEGVGVNVRPYEVE
GYYTADSVEAVSTLTSKAVKAGATIFNALSVEDVVVDDEERINGLVVNWTAVEMAGLHVDPLSIHCKYVIDATGHDTEVVRVVARKMPGRLFTATGN
IEGEKFMSPDRAEKLTIVNTREVFPGLYVAGMAANATFGGPRMGPIFGGMLLSGVKAAREILSKI

>Acidianus hospitalis (strain W1)
MQSIRIKQVNEVKISKYILKYTFEDWNNLVESDVVIVGAGPSGMTAAYYLAKAGLKTVIFERRLSFGGGIGGGAMNFHKIVIETPADEIIKELKIRY
IEPEEGIFIIDSAEFMAKLATAAIDAGAKIIHGVTVDDVIFRENPLRVAGVAVEWTSTQMSGLHVDPLFISAKAVVDATGHDAEIISVASRKVPELG
IAVPGEKSAYSEIAEELVVENTGKVAPGLYATGMAVCEVKSLPRMGPIFGAMILSGKKVAEEIIKDLRNS

>Acidithiobacillales bacterium SM23_46
MCCQSLEAWRSEPEERKRNADVVVVGAGPSGMTAAIHLARERHRVILLEKRLSPGGGIWGGGMAMSEAIVQDDALPWLDDLGVRHKPSRGGLHSADA
VELAAALCLKTVQSGTVLFNLLTVEDVCIHQDRVTGVVVNRSMIAGALPVDPIAFRTNAVIDATGHEAVVVEAVHKRGLLAHPAVAKPLGEGPMDAA
SGEAFVVENVKEVYPGLWICGMSVCATLGGPRMGPIFGGMLLSGQRVAALVSSALTEFAQKDRESRK

>Aciduliprofundum boonei (strain DSM 19572 / T469)
MLDEVEITKLIVENYMKDLMEYADLDVAIVGAGPSGLTAAYYLATAKKKVAIFDRRLSIGGGMWGGGMMFNKIVVQEDAKHILDDFSINYERFGDYY
VADSVHSVTSLAYHATKEGAKIFNLIGAEDVIIKNNRVSGLVINWSVIGELPIDPLSIYAKYVIDATGHESEVIKTLVRKNNIKLNTPTGSIEGEHS
MDADTAESVIVDNVKEVYPGIFVTGMAANAVFGSPRMGPIFGGMLLSGKKVADEIIRRLS

>Actinobacteria bacterium HGW-Actinobacteria-3
MPLSEIEVTRGILEGFSRDFLSSLQSDVAIAGAGPSGMVCAYYLAREGLKVSVFERNLHVGGGMWGGGMLFPRIIIQEAAREIVEEFGVRLKPFKEG
YFVGDSVETVSKVTAAAIDAGVRVWVGVSVEDVLIREENRLAGVVLNWRAVELANLHVDPLAVEAKVVVDATGHEAGVVRTVARKIPGCRLNTDTGG
VIGEMPMWAQVGEELIVGNTREVYANLLVTGMAANAVYGAPRMGAIFGGMFLSGYKCAHLAADIVRKA

>Alistipes inops
MIETKVSQGIVSTYFDKLQKNLELDVAIVGGGPSGIVAAYYLAKAGLRVAQFDRKLAPGGGMWGGAMMFNQIVIQEEAMDIVREFGINYAPFGEGLY
VMDSVESTSALLYHAVHAGATVFNCYSVEDVVYKENRVSGVVVNWTPVLREGLHVDPLNILARVVIDGTGHDSEIAATVARKNGARLNTETGGVVGE
RSLDVTAGEDEVVKGTKEIYPGLYVCGMAASAVSGTPRMGPIFGGMLMSGKKVADEIIARLKK

>Ammonifex degensii (strain DSM 10501 / KC4)
MAGGAIDERLVSRAIIQTYSEELLQLTDFDVAVVGAGPSGLTAAYYLAQGGLKTVVFERRLSVGGGMWGGAMMFNYLVFQEEARPIFETMGVRYREY
QPGYYVAHSVEAVAAFTLAACRAGARIMNLITVEDLVLRDNRVAGLVLNWTAVDMAGMHIDPLAVHCRYVVDATGHDAEVVRILTQKNQVTVKVPGG
HVQGEKSMWSERGEKQTLDHSGEVFPGLYVAGMAANAVAGGYRMGPIFGGMVLSGKKVAELILEAHRREKSQTL

>Ancylomarina sp. 16SWW S1-10-2
MEQIVSAGIVDSYFKKLKENLSVDVAIVGGGPSGLVASYYLAKKGFKVALYETKLAPGGGMWGGAMMFNEIVVQKDALHILDELNVSYTNYQGDYYT
LDSVHATSALIYHATQAGVKIFNCSSIEDVVFQNNKVCGVVLNWSPVRREGLHVDPLVIMAKAVVDGTGHECDIVSTLERKNGVKLNTKTGKVMGEC
SLSIDEAERTTVENTKEVYPGLYVSGMASNGVSGGFRMGPIFGGMLLSGEKLAGLIAENLSK

>ANME-2 cluster archaeon HR1
MELDEITITRAIIEDFTSDFLQSIDTDVALVGGGPANLIAARTLARAGVKTVLFERKLEVGGGMWGGGMMMPRIVVQEEARHILDDLGVRYRKYEEG
YYVADSIECTGKLIYEAASSGASIYNLISVEDVMIREGDAVTGLVINRTIVDMQKLHVDPITIRAKVVIDGTGHDAEICTTLSRKIPGALHVAGEKP
MWADVAERIILDNTKEVYPGLIVTGMAANAVAGAPRMGPIFGGMLLSGEKAAQIAIAKLGL

>Archaeoglobales archaeon ex4484_92
MEARISKAIIEEVAKDWSNISQVDVVIVGAGPSGLTAGKYLAEKGLKTLILERRLCFGGGIGGGGMLFHKIVIEKFAREILDDFDVRYYEHDNLLVA
DVAEFMAKLAVGCVNAGTKIIHGVSVCDVIFRLEPIRITGVCIQWSAVELSGLHVDPMFIESKAVLDATGHDAEVVSIASKKVPLDLNVTGEKSAYA
ELGEKLVVEKTGKVVEGLYATGMAVCSVFNLPRMGPIFGGMLQSGKKAAEIIYNDLK

>Archaeoglobus fulgidus
MEAEITKAIVETASEEWVEYAESDVIVVGAGPSGLTAARYLAEKGLKTLVLERRLSFGGGIGGGGMLFHKVVVEREAKDILDDFGIRYTEHRNFLVA
DSAEFMAKLAAKAIDAGAKIIHGVSVEDVIFRDDPLGVRGVCIQWSAVEISGLHVDPLFLRSRAVVDATGHDAEVISVAARKIPLEVSVVGERSAYS
EVAEREIVEKTGKIVKGLYAAGMAVAAVHNLPRMGPIFGGMLLSGKKVAEIVAEDLKL

>Armatimonadetes bacterium
MSFEKDSFKWDELTVTRGIVETFMADFLDSIDLDVAVVGAGPSGITAARILAGQGHRVGIFERNLHIGGGIWGGGMLFPRIVIEEEAAPLMEAAGVK
LRPWKDGTVIADAVESATKMTAAAIDAGARIFVGIEAEDVVVDDSDRVCGVVINWGAVTAAKLHVDPLAVHSKVLIESTGHPCEVGDVLLRKIPGAR
LDTGETCVPGEASMNARAGEAALIANTREIYPGVVVAGMAANAVSRSPRMGAIFGGMLLSGQKAAEISAQIIADLG

>bacterium (Candidatus Ratteibacteria) CG23_combo_of_CG06-09_8_20_14_all_48_7
MLDDVVISRAIVETYFQDLLNYLENDVAIAGAGPAGLTAAYFLAKKGRKVAIFERQLRVGGGMPGGGMMFNKIVIQEETKKILDEFGIRYQKYQNGY
YVADSLETTSLLTSKAIQAGAKIFNLIAVEDLSLQEGRVNGLVLNWSAVKTAGLHVDPITIRAKAVVDATGHDAVLCRLLVDKGKVTLRTPTGKVAG
EGPMWAEKGEEMIIANTGEVFPGLFIAGMTVNAVCGGPRMGPIFGGMLLSGERLSLLIP

>bacterium (Candidatus Stahlbacteria) CG23_combo_of_CG06-09_8_20_14_all_40_9
MMLDETIISRAIIETYKEKFVNILKSDVAIAGGGPSGLIAGYYLKKKKPDLKVVLFERKLSIGGGMWGGGMMMNEIVVQEEGKKILDEFGVKSKKFE
NGYYTADSIETVSALALNTVKAGVTILNAISVEDTIIEDNIAKGLVINWTSALDIGLHVDPLALRADHIIDATGHPCEIAHLIEKKGKKLFTRTGKI
IGEGAMYADKGERVIIENTKELFPNVWACGMAANAVFGGPRMGPIFGGMLLSGKIVAEKILQKN

>bacterium 42_11
MKDILISKAILESFFNKLRDSLELDVAIVGAGPSGLVASYELAKKKVKIAIFEERNTPGGGIWGGGIMFNEVVLEKELEDFLKELDIKYKYVEDYIV
VDSTHFASALIYHTTTWGTRIFNSISVEDIAMQNNRVCGVVINWGPVKKLGLHVDPITIKASYVVDGTGHPANVVSLLVKRGLLEKKTEFPMNAEEA
EKFVVEKTGEVFPGLLVSGMAVCEVYGGPRMGPIFGGMVLSGRRIAEIITERVNKR

>Bacteroidales bacterium 6E
MEQIVSSGIIDSYFKKIKESLSVDVAIVGGGPSGLVAAYYLAKKGLKVAMFERKLAPGGGMWGGAMMFNEIVVQKGALQILDEFKIDYTHYEGDYYT
LDSVQATSSLIYHAGKAGARIFNCTSVEDVVFHNNKVSGIVLNWAPVHRERLHVDPLVIMAKAVIDGTGHDCDIARILERKNNIQLNTVSGKVQGER
SLSIDEAERTTVENTKEIFPGLYVSGMAANGVSGGFRMGPIFGGMLLSGEKVAALIMNQLNK

>Bacteroides cellulosilyticus
MIETKVSKGIISTYFEKLERNLDLDVAIVGGGPSGIVAAYYLAKAGLKVAQFDRKLAPGGGMWGGAMMFNQIVIQEEAIDIVKEFNINHEKYEDGLY
VMDSVESTSALLYHAVHAGATVFNCYSVEDVIFKNNTVSGVVVNWTPVLREGMHVDPLNILAKIVIDGTGHDSEIAATVARKNGSRLATETGGVIGE
RSLDVIAGEEEVVNGTKEIYPGLYVCGMAASAVSGTPRMGPIFGGMLMSGKKVAEEIIAKLKK

>Bacteroides sp. 3_1_19
MEQIVSSGIIDSYFEKLKSNLSVDVAIVGGGPSGIVAAYFLAKAGKKVALFDRKLAPGGGMWGGAMMFNDIVVQEEAMPIIKELGVSYKEGANGTYI
MDSVHTTSALIYQATKAGATIFNCYSVEDVVFHNDAVAGVVVNWAPVIREGMHVDPLTIMAKAVLEGTGHDCEIARVVARKNDIQLNTPTGGVIGER
SLNVELGEQTTVENTKEIYPGLFVSGMAANGVSGSFRMGPIFGGMLMSGKKAAELICEKLGN

>Bacteroides stercorirosoris
MIETKVSKGIISTYFEKLERNLDLDVAIVGGGPSGIVAAYYLAKAGLKVAQFDRKLAPGGGMWGGAMMFNQIVIQEEAIDIVKEFNINHEKYEDGLY
VMDSVESTSALLYQAVHAGATIFNCYSVEDVIFKNNTVNGVVVNWTPVLREGMHVDPLNILAKVVVDGTGHDSEIAATVARKNGIRLATETGGVIGE
RSLDVVAGEDEVVNGTKEIYPGLYVCGMAASAVSGTPRMGPIFGGMLMSGKKVADEIIAKLKK

>Bacteroidetes bacterium ADurb.Bin035
MEQIVSNGIIDSYFNKLKQYLSVDVAIVGGGPSGLVASYYLAKNNYKVAIYERKLAPGGGMWGGAMMFNEIVVQKAALHILDELSIEYREFEHDFYV
IDSVHAASALIYNASKAGVKIFNCTSVEDVVFLDNKVCGIVLNWAPVAREHLEIDPLVIMAKVVVDSTGHDCDVAHTLERKNNIKLNTETGKVIGER
SLSINEGENSTIDNTKEIYPGLFVTGMAANGVSGSFRMGPIFGGMILSGKKVADLIINKIKNT

>Brockia lithotrophica
MFDERVITRAIVETYLEEFRSIVDLDVAVVGSGPSGLVAARELARRGYRVAVFEKRLSVGGGLWGGGMLMNRIVVQEAARPILEEFGVRTREYAPGV
YVAHSVETVAALVFGALQAGAYVFNGVAMEDVVVRDGRVAGLVLNWGAVHAAGLHVDPLAIHARAVLDATGHDAEVVRKLAAKNGVSLSVSGERSMW
ADRGEEEAVALTGEVYPGLFASGMAATNVYGGHRMGPIFGGMLLSGVRAAELIAEALGS

>Caldimicrobium thiodismutans
MELQINQAIIREGMRDLEDFSDVDVLIAGAGPSGLTAAKYLAERGFKVLIYERRLSFGGGIGGGGNMIPKIVVQTEALPIVEDFKIRAKKVENGFFT
IDPAELIAKLATGALDAGAKIFLGVNVDDVIVRDAPPRVVGVLWHWTAIQLSGLHVDPLYTHCKALVDATGHDAELIAIAGRKNPELGIEMAGEKSN
WSEVSERLVVEHTGRVAQGLYVTGIAVCAVYGLPRMGPIFGGMLMSGKKLAEIIEKDLKNASKGKGKGKKAG

>candidate division MSBL1 archaeon SCGC-AAA259E19
MLDEEVITKAIVEEYMRTFLENTDVEAALGGAGPANLVAAKKLAEKGVKTAVYEEKLNVGGGMWGGGMMYPRIVVQEEAKRILKEFGINFSEYEKGY
YVASSIESVARLASEAVKAGAEIFNLTKVEDVMVREDDHIAGVVLNWSAVEKANLHVDPLTVKADVVIDGTGHDAEICRVTQRKIPDADLQVRGEKP
MWAEKGEKALMDTTKEVYPGLIVSGMAANAVSAGPRMGPVFGGMLLSGEKAAELALERIEEK

>candidate division TA06 bacterium DG_78
MIEETIISKAIVESYLKDVLDSIESDVIIAGAGPSGLCAGYYLAKHKYKTVLFERGLKLGGGMPGGGIMFNKIVVQDEGKATLDEFGITYHQYEPGY
FVADSLETTACLTEKALKAGLKIFNLVSVEDVMIREGCVTGVVINWSAVEMARLHIDPISFKSKVVIDATGHPSEIVHIVEKKSGGKLLTPSGRIEG
EKSMWAEVAERLSLENTKEVYPNLYVCGMAANAVFGGPRMGPIFGGMLLSGKQVAELVMKKV

>candidate division WOR_3 bacterium SM1_77
MIEETVISRAIIESYLHDLLDSIQSDVIIGGAGPSGLCASYYLAKKGHKVVLFERALKLGGGMPGGGIMFNRIVVQETARRILDEFAIRYEEYKPGY
YVANSLEATAALTDKAIKAGAKIFNLITIEDVVVREQVVSGVVINWSSVEMAKLHIDPISFESKFVIDATGHPSEIARIVERKNSGTLLTPSGKIEG
EKSMWAEVAEQFTVENTKEIFPNLYVCGMAANAVFGGPRMGPIFGGMLISGMKVAELVMKKI

>candidate division Zixibacteria bacterium 4484_93
MNFTDIKISKAIIDDYFRKLTSSLELDVAIVGAGPAGLTAGYFIAKQGYRVSLFERKLSAGGGMWGGGMMFNTIVVGKGANDILDEFGIRYRRTEDS
LYIADAVESVGALIYRSTQAGLRIFNLITVEDLLVKDGKVEGLVINWSPVEMASLHIDPLTVQSRYSVDATGHPLEVVRTLCKKSGVQLFTETGDVL
GERSMCADEGEKFVLEKTGEVAPNMFVAGMAACAAFGGPRMGPIFGGMLLSGKKVADIITKRLKEKK

>Candidatus Acetothermia bacterium
MKIDDVLVSRLLIEEYMADFLDCLHIDVAIVGAGPAGLTAAYYLAKAGAKVAVYERKLAIGGGMWGGGAMFSRIVIQEEAKQILDQFKITSIAKEDG
YYVADAIEAITLLAAGAIQAGAKVFNLIHIEDLLLRNDRVEGLVLQWSPVEMGGLHVDPITIGAREVIDATGHDCEVVKKLLHKGGVKIDTETGGML
GERPMWAEKGEKMTVQYTKQIYPGLYVAGMAVGAVFGTPRMGPIFGGMLLSGKKAAQIIAQTLS

>Candidatus Acidianus copahuensis
MKVKQVDEGKISKYILKFTFEDWENIIDSDVIIVGAGPSGMTAAYYLAKAGLKTVLFERRLSFGGGIGGGAMLFHKIIIESPADEILKELGIRLVKA
EEDVYAVDTAEFMAKLASSAIDAGAKFIHGITVDDVIFREEPLKVAGVAVEWTATQMSSLHVDPIFISAKAVVDATGHDAEVISVASRKLPELEISI
PGEKSAYSEVAEQQVVDGTGKVAPGLYAAGMAVCEIKGLPRMGPIFGAMVLSGKKVADEIINDIRKS

>Candidatus Altiarchaeum sp. CG2_30_32_3053
MIEETKITELIVRNAVDDFLNNLDVDVVVAGGGPAGLTTARYLAKAKKKVVLFERKLSIGGGMWGGGMMFPRVVLQKGGKEILEECDVKCKKSGGLW
VADSIECVTKMTAKAIDEGVKIFNLVSIEDVIIRNNEDNKNNKNRKNKTKICGVVINWTAVQMANLHVDPLSVKSKFVVDATGHEASISHLVVKKVG
NLNTKTGDVLGERSMWAEKGESDIMGNTGEVYPGLFVSGMAANAVYGSERMGAIFGGMLMSGKKVSELILKKI

>Candidatus Aminicenantes bacterium 4484_214
MIDEIVISRAITEAYLKEFLDCLESDVIISGAGPAGLCAALNLAQEGYKVVVFERTLRPGGGVPGGGMMFNKIVIQEEARPLLEELDVTLKPYQENY
YVVQALELLGALLVKAIKQGVYLFNCISVEDVLIYDKKVSGVVINWSAAQAAGLHVDPLTARTKFVVDATGHAAEVAEIVSRKSGCKLFTSTGKVTG
EKPMWAEEGEKILLTNTKEVYPHLYVCGMAANAVFGGPRMGPIFGGMLLSGQKVAQLIAARLKTK

>Candidatus Aramenus sulfurataquae
MQSIRIKQVDEVKISRYILKYTFEDWYSLVDSDVVVVGAGPSGLATAYFTAKAGLKTVVFERRLSFGGGIGGGAMNFHKIVIESPADELLREWKVKL
VEAEEGVFIVDAAEFMAKLGAAAIDAGAKVIHGINVDDVIFRDKPLRVAGVAVEWTSTQMSGLHVDPLFVSAKAVVDATGHDAEILSVASRKIPELG
IVIPGEKSAYSEVAEELVVNNAGKVAEGLYTTGMAVCEVKSLPRMGPIFGAMVLSGKKVAEDIINDLRNS

>Candidatus Aureabacteria bacterium SURF_26
MPLDDLIVSRAIIDEYHTTLVDALNMDVAIVGGGPAGLVAGYYLAKQGYKVSLFERKLSIGGGMWGGGIMFNKIVLQEDALKVLNEFNVRVKKYRDN
YYVADSVETVGALIYHATQAGLQIFNCMSIEDVKITADDAVCGLVVNWTSVELTNMHVDPITFGAKFVIDATGHSCDMANIILKRIGKVLFTPTGDI
MGEGSMNAELAEKVVAENSREIYKNLYVTGMAANAIWGSKRMGPIFGGMLLSGKKVADDISARLAQEAVNA

>Candidatus Bathyarchaeota archaeon B26-2
MRIKEVDEAVVTKAILEGSLKYLHELTEVDVAVVGAGPAGLTASRYLAKAGLKTVVFERRLSFGGGIGGGGMQLPMLVVQSPADEILREVGCNLTTY
REGVYLANSSEMLAKLAYSAVKAGAHIILGVTVDDLIYRSEENRTRIVGVVVQWSSVIISGLHVDPLAFKAGAVVDCTGHDAEVLSVASRKIPELNL
MIQGEKAMWVSESERLIVEKTGEVSPGLYVAGMAVATLNQTPRMGPIFGGMLLSGKKVAEIIIERYKQNRTQG

>Candidatus Desantisbacteria bacterium CG2_30_40_21
MQLDDVVISRAIIESWNKDLLDSLDIDVAIVGGGPAGLTCGYYLSKKGLKVVLFERNLSIGGGMWGGGMMYNKCVFQQESLPILNEFGIRTQEYQDG
YFITDSLETVTTLCSGALKAGLKIFNLIGVEDVMIRQEGVTGLVLNWTAVTMAKLHIDPLTIRAKAIVDSTGHAAEVAGIIVRKIGKKLLTETGEML
GEKPMWAEVGERTIAENTKEIYPGVFVAGMSANAVFGGPRMGPIFGGMLLSGKQAAEIIAARL

>Candidatus Desulforudis sp.
MKIDETVVSRAIIERYTQKLLSCLDVDVEIVGGGPAGLTAAHYLAKHGVKTTLYERKLSVGGGMWGGAIMMNEIVFQEQARPLFEEFGIRINPYSDG
YYTASSVECVAALTLQACQAGANIINLMTVEDVVLHEDRVSGLVLNWTAVDIAGLHVDPIATRSKYVIDCTGHDMEVANILSCKAGVKLVTPSGEPV
GEKPMWADVGENQLIGHTIEVYPGLFVAGMAANAVNGGYRMGAIFGGMVLSGRRAGELILKRLQS

>Candidatus Fermentibacteria bacterium
MENIVTSAIAEDYHRKIQESVTAHVAIAGGGPSGLVAAAELASRGLSVNLYEKNLTPGGGMWGGAMLFNSILIEEEFAETAEELGMKLRKYRDSVLL
ADSVQATAALISRACSAGVRMFNGMAVEDVTVDRYDRVNGVVVNWAPVMKLGMMVDPLMTCAGAVLDATGHPAEIVTRFAAKNNTEITVPGEASLNV
EMGEKHTVEHTGMVHPGLFVSGMSACATAGGYRMGPVFGGMLKSGLKAAEEITEYLNKSQ

>Candidatus Korarchaeota archaeon
MFPVEESVISSAIIERGSKFLVDLVKSDVIIVGAGPSGLVAGRYIAKTGLKTCIIERRLSFGGGIGGGGMLFPRIVVQEPAQEILEEVGVKLEPYSK
GVWIADVAETIAKLAAGAIDSGARILLGANVEDLIVRNSRVCGVVVQWSAVTSAGLHVDPLAFESRAVIDCTGHNAEVVAIAARKNPELGIRVLGEH
SMDAVRAEKEVVELTGEVLPGLWVAGMAAAAVRGGPRMGPIFGGMLLSGKKVAELVSRELEVL

>Candidatus Nitrospira inopinata
MHKPKPAPLRERDVTRHIAREYYKEFDQLIESDVIIVGAGPSGLLCAHDLAAMGFRTLIVEQSLALGGGFWHGGYLMNKATICEPANEILEELGVPC
KRIADCDGMYMVDPPHATGALVAAAYRAGAKILNLTRVVDLILRQDGLLEGIVVNNTTAEMAGHDVIHVDPIALESKIVVDATGHDAVVVELLHKRN
LYKPVPGNGAMWVSRSEEEVVDRTGEVYPNCFVVGLAVAAVYGTPRMGPAFGSMLLSGRYGAQLIKKKLKQE

>Candidatus Nitrospira nitrificans
MAKPRPAPLRERDITRHIAREYYKEFDQLIESDVIIVGAGPSGLICAHDLAAMGFRTVLIEQSLALGGGFWSGGYLMNKATICEPANEILEEVGVPC
KKIKECEGMYMVDPPHATGALIAAAYKGGAKIMNLTRVVDLILRNGGLLEGVVVNSTTAEMAGHDLIHVDPIALESKIVVDATGHDAVLVELLHKRN
LYNKVPGNGAMWVARSEEEVMDRTGEVYPNCFVIGLAVAAVYGTPRMGPAFGSMLLSGRYGAELIKKKLKQE

>Candidatus Nitrospira nitrosa
MTKPRPAPLRERDITRQIAREYYKEFDQLIESDVIIVGAGPSGLICAHDLAKMGFRTLIIEQSLALGGGFWHGGYLMNKATICEPANEILEEIGVPC
KKIRECEGMYMVDPPHATGALIASAYKAGAKVLNLTRVVDLILRRDGILEGVVVNNTTAEMAGHDVIHVDPIALESKIVVDATGHDAIVVELLHKRN
LYQKIPGNGAMWVSRSEEEVMDRTGEVYPNCFVIGLAVAAVYGTPRMGPAFGSMLLSGRYGAGLIAKKLKNE

>Candidatus Syntrophoarchaeum butanivorans
MDEVTISKAITESYMKDLIDSMVLDTVVVGAGPAGLLAAYNLAREGVKVAVFERRLSVGGGMWGGGMMFSRIVVQDAGREILDEIGVRCSEYEPGYY
IADAIEAVTTITSEVIRAGARIFNLMSVEDVVVRDDDRIHGVVINWSAVELSKLHVDPMTVIADYVIDATGHAAEVARIVEQKLGGGALTVRGERPM
WAEAGEAAVVENTKEIYPGLIVAGMAANAVLGSPRMGPVFGGMLLSGRKAAELVLSRL

>Chloroflexi bacterium RBG_13_51_52
MVKFSPVGEVVITRAIVEEFAKEFNEYVESDCIIIGGGPSGLVAGRDIARAGKKVVIIERNNYLGGGFWSGGYLMPKVTVRHPGEKILDELGVPYKT
VAKGLVVCDAPHACAALIAAACAAGVKIFNMTMLEDLVVKDGRVCGAVINWSPIASLPRQVAALDPVAIEAKVVIDATGHDATVVAKLEKRNLIKMK
GEGAMWIEKSEDLIVEHTGECFPGLIVTGMAVGAVYGLPRMGPTFGSMFLSGEVAAKVALEKMK

>Clostridium drakei
MYLEDTKISKAIIDTYKDKLEDILHSDVIIVGGGPSGLVGASYLAKAGIKTTLLERNLSIGGGMWGGGMMMNQIVIQESAKSILDEFNIGYKKYEEN
YYTADSIECVSALTLSASQSGARILNSISVEDVIVKDKCISGLVINWTAVEKTRMPIDPIMIESKYVLDATGHDASVVNKLVTRMGNVLNTPNGTLE
GEKPMWADRGEEQVIKNTREVYPGLYVSGMAANATFGGQRMGPIFGGMLISGQKVAQELIQKIKNC

>Clostridium ragsdalei P11
MYLEDTKISKAIIDTYKNKLEDVLHSDVIIVGGGPSGLVAASYLAKAGIKTTLLERSLSIGGGMWGGGMMMNQIVIQESAKSILDESNIGYKKYEEN
YYTADSIECVSGLTFNAAQAGARILNLITVEDVIVKDKCISGLVINWAAVEKTRMPIDPIMIESKYVLDATGHDASVVNKLVTRMGNVLNTPNGTLE
GEKPMWANRGEEQVVENTREVYPGLYVSGMAANATFGGQRMGPIFGGMLISGQKVAQELIKKIKNC

>Clostridium sp. JN500901
MYLEDTKISKAIIDTYKDKLEDILYSDVIIVGGGPSGLVAAAYLAEAGVKTTIIERSLSIGGGMWGGGMMMNQIVIQESARSILDDFNVNYKKYEEN
YYTVDSIECVSALTLKAVKAGAKILNLISVEDVIEKDNCIAGLVINWAAVEKTKMPIDPIMIESKYILDATGHDASIVNKLVAKQGNVLNTENGSFV
GEKTMWADRGEDQVVKNTGEVYPGLYVSGMAANATLGGQRMGPVFGGMLLSGQKAAKQLIEKLHAAK

>Coxiella sp. DG_40
MEQITTLGIVDSYYQKLKDNLFIDVAIVGGGPSALVAAYYLAKLQKKVAIFERKLAPGGGMWGGGMMFNQIVVQSEALSILDEFKISYALFKDNYYL
VDSIESTASLIYHTIHAGAKVFNCYSVEDIVLKNKKVSGIVVNWGTVHDQGLHVDPLVVVAKCVIEATGHSCEVAKVLAKKNGIKLHTETGGVVGEK
SLAMEQAERSTIENTKEIYPGLYVCGMAANGVSGDFRMGPIFGGMLMSGKKVAEIIVKDIT

>Dehalococcoidia bacterium
MPLFHPVTEGEITRAIVNSFLRQFEEYVSSDVIIVGGGPSGLMAGRELGKAGLKVIVIERNNYLGGGFWAGGYFMNKLTLREPAQEVLDELGVPYSR
AGEGLYVADAPHACSKLIGAAADSGVKFFNLTLLEDLVVREDKRVAGAVINWSPIAYLPREIAALDPVPLETKVIIDATGHDASVARKLERRGMLKL
AGEGALWIEESEEAVVEHTGEVYPGLVVTGMAVASVYGLPRMGPTFGGMLLSGKRAAEVALAAVLTDSR

>Desulfacinum hydrothermale DSM 13146
MALDERIITRAIMDRYIAKLKEAIDLDVAIVGAGPSGLVAGMLLAEAGKKVALFERKLSVGGGMWGGGMLFNEIVVQEEAKTILDQVGIRAQHYTDG
YYTADAVESVSTLTSRSVKAGARIFNCVSVEDVMMRPERIMGLVLNWSAVEMAGLHVDPLAVRCQVVVDATGHDTEVVKVVERKVPGSLSTPSGKRA
GERSMWAEEAERLTLENTCQVYPGLYVAGMAANATFGGPRMGPIFGGMLLSGQKVARLILEQLQS

>Desulfarculus sp.
MLEEVTITRAIIRRYLGKLDQSLELDAAIVGGGPAGLVAGKKLAQAGYKTALFERKLSVGGGMWGGGMMLNEIVVQQEARRILEEFGVPSSEFAPGY
YTADSVLATSTLCSVAAKAGLTIFNLVSVEDVVIRAQRVTSLVINWSAVQMAGLHVDPLTIKARWVIDATGHDSEVLHVIARKVDAELLTASGKVMG
ERSLWAEQAESDTLANTREAFPGVYTAGMCANAVFGSYRMGPVFGGMLLSGEKAAAEVAARLAAGE

>Desulfatibacillum aliphaticivorans
MEERITSAIVRTYFEKLQNFLEVDLAIVGAGPSGLVAAAALAKEGKKVAIFERLLAPGGGVWGGGMLFNEIVIQEEALHILDDFNISYKSAGDGLYT
ADSVEVASGLIFGAKKAGVMINNAVSVEDVVCREGRICGVVVNWTPVERLGMHVDPLVVMSKAVLDGTGHPGEITDLATRKAGIKIDTPTGKIMGEK
PMWMELGEASTVENTKCLYPGLYVSGMAANNASGGFRMGPIFGGMFMSGRKVAKMILEDIDG

>Desulfobacca acetoxidans (strain ATCC 700848 / DSM 11109 / ASRB2)
MGLDEIIISRAIIERFMEKFLDNLELDVAIVGGGVSGLVAGWRLAQKGRKAAIFERKLSVGGGMWGGGMMFNEIVVQEEAKHLLDELGITSRPYDRG
YYTADAIESTTTLASQAMKAGVKIFNLIHVEDVMVRENRIDGLVILWTAVNMAGLHVDPLTIRAKHVIDCTGHDVEVIKIFLRKNQPASLKTETGGI
MGERSMWAEVGEAKTVEYTSEVYPGLWVAGMTATGTLGTFRMGPIFGGMMLSGEKAANLIDERLKKG

>Desulfobacteraceae bacterium
MQLDDVAISKSILDAYFEKLLARLDVDVALVGAGPANLVAGYYLGKSGFKAVVFESKLAPGGGMWGGGMMFNEIVLQDDAVHIAEELGIHCNPGGDG
YYTMDSVEATSTIISRCVRAGTVIFNLIKVEDVLFRQDDRQPRVSGLVINWSPVEKLGLYVDPLSIRASFVVDGTGHPADICRTVARKMDVKLNTKT
GNVVGEMPLWAEKGEQFTVTNTAEVFPGLYVAGMAANNAFGGPRMGPIFGGMLRSGKKVAEILAEKLRSD

>Desulfobacterium sp. 4572_20
MAINEVVISKAIIDRFSQKFMEYTEVDTAIVGAGPSGLIAAYFLARAGQKVALFERQLSIGGGMWGGGMMFNEIVVQTQGKELLEMFGISAREYEPG
YYTADAVECVTTICSNAVKAGAKIFNCMSVEDVSIREDRVMGLVLTWSAVEAARMHVDPLTIAAKYVIDATGHDTEVIRLIEKKADIALQTETGKIM
GERSMWADKAEQLTIENTKEICPGVFVSGMAANAAFGGPRMGPIFGGMLLSGKKVAELIMAKEGSAFETSAEDFDSWFNRNQAIFESELLAQQQFIY
SL

>Desulfocarbo indianensis
MLDEITITRAIIDRYFEKLNRNLELDAAIVGGGPSGLIAGYKLAKAGYRVAMFERKLSIGGGMWGGGMMMNEIVVQEEAKRILDEVQVPTREFQPGY
YTADSVLCTSTLCSQAAKAGLTIFNLVSVEDVMVREQRVVGLVINWTAVEMAGLHVDPLTIRAKYTIDATGHAAEVMHVIARKVDAKLFTDDGKVAG
ERSLWAEVAETNTVNNTREAFGGVFTAGMCCNATFGSYRMGPVFGGMLLSGEKAAQLVAERLQAEK

>Desulfococcus oleovorans (strain DSM 6200 / Hxd3)
MELNEVTISRAIIDRFYEKLIANLEVDVAVVGGGPSGLVAAWRLARAGRKVALFERKLSIGGGMWGGAMLFNEIVVQKSALHVLDAMEIGYRLYAED
YYTADAVEAISTLTSQAAKAGVAIFNCVTVEDVMIRPDRIVGLVLNWSPVEMAGLHVDPLAMRASFVIDATGHATEVVHVVAKKVPGTLRTDSGKIE
GEKSMWSDRAESLTLENTREVYPGLYVAGMAGNATFGGPRMGAIFGGMLLSGEKVAAEILERLE

>Desulfofundulus australicus DSM 11792
MMHLEDVVISKAIISRYQEELLEALESDVAVVGGGPSGLVAAYYLARANKKVVLFERKLSIGGGMWGGGMMFNQIVIQDEALPLLEEFKISYRVFEE
GYYTASSVEAVAALTLGAVRAGAKIFNLISVEDIMVRDNRVAGLVINWTPVDLGRLHVDPLTVQSSYVIDCTGHDAQVAGMIVKKMGAVLKTTTGTL
EGEKPMWAARGEMATVANTREVYPGLIVAGMAANAVCGGHRMGPVFGGMLLSGQRAARIILEGDKT

>Desulfofustis glycolicus DSM 9705
MLNEVTISTAIINRYMTKLTSALDLDVAIVGGGPSGLVAGYYLAKAGRKVALFDRKLSIGGGIWGGGMMFNEIVVQEAGAAVLAEFGLAGSPFEPGY
YTLDSVYTTATLVHKAMAAGLLIFNLIGVDDVVIKDERVAGLVINWGAVSTLGWHIDPLTLFARYVLDATGHDAEIASVLVRKMGVRLNTETGGLVG
EKSMAAERAERETVTNTREVYPGLFVSGMAANAVCGGYRMGPVFGGMLLSGKRAAESILEGLA

>Desulfonatronospira thiodismutans ASO3-1
MALDEIIISRAIIETYTKKLMDSLELDVAICGAGPSGMVAAYYLASAGKKTAVFERNLAPGGGMWGGGMMFNEVVVQEEAREILDELDIKSVEYTPG
YYTADSVEAVCTLGSKAAKAGARFFNLVCIEDVMIRENRITGLVINWSAVESAGLHVDPLTVRADYVVEATGHPVEIMQVIESKMDTRLNTPSGRLE
GEKSMWAEKAEEHTIENTTEAFPGVYVCGMSANATFGSFRMGPVFGGMLRSGKKVAQEIINKAK

>Desulfonauticus sp. 38_4375
MSLDEKIISEAIISKYFEDFKRCLNLDVAIVGGGPSGLTAAYHLAKEGFKVALFERKLSIGGGMWGGGMTFNYIVVQEQGKQILEEMDIICEEYKPG
YYVVDAVLATTTLASKACKAGAKIFNCMSVEDVVIREEGKVKRVAGLVVNYSPVEIAGLHVDPLVLETKFVIEATGHDTEVLKTLVRKNDIKLFTPS
GGIEGEKSMWAEVAEENTLKNTREAFPGIYVCGMAANACFGSYRMGPIFGGMLLSGVKVAEEISTRLKEGK

>Desulforudis audaxviator (strain MP104C)
MKLDETIISRAIIESYVTRLLSCLEVDVEIVGGGPSGLTAAYYLARAGLKTTVYERKLSVGGGMWGGAAMMNEIVFQETARPVFEEFGVTIKKYRDN
YYTASSVECVAALTLGACRAGANIMNLLTVEDVVLHNNRVSGLVLNWSAVEISGLHVDPIATRSKFVVDATGHDVSVVGVLARKAGVQLDTPSGKVQ
GEKPMWADLGEAQIMENTSEIFPGLYVVGMAANAVHGGYRMGAVFGGMVLSGRRVAEMIIDRLKV

>Desulfovibrionaceae bacterium CG1_02_65_16
MIIDERIVSEAIASTYFGKFKSCLDLDVAIVGGGPSGLTAAWKLAKAGRKVALFERKLSIGGGMWGGGMTWNSIVVQESAKSILEDAGVALSEFKPG
YFTADSVAATAALAYQATHAGAHVFNCMSVEDVVLREVEGVKRVIGLVVNSSPVEIARLHVDPLVLHCKHAIECTGHDVEMLKTLVRKNDVRLDTPS
GGIEGEQSMWADVAEANTVRYTREVFPGVWVAGMAANAAFGSYRMGPIFGGMLLSGVKVAETIDALL

>Desulfovibrionales bacterium GWA2_65_9
MIIDERIVTEAIASAYFEKFKQCLDLDVAIVGGGPSGLTAAWKLAEAGRKVALFERKLSVGGGMWGGGMTWNYIVVQEEAKGILEEAGCAMSEYKPG
YFLADSVAATAALAYRATKAGAHVFNCMSVEDVVLREIDGEKRVMGLVVNSSPVEMARLHVDPLVLHCKHAIECTGHDVEMLKTLVRKNGVKLNTPS
GGIEGEQSMWADVAEANTVRHTREVFPGVWVAGMAANATYGSYRMGPIFGGMLLSGVKVAEEINARL

>Desulfurella amilsii
MALDERIISRAIIERYFQKLLANIDCDCAIVGAGPAGLVCGYELVKNGLKVTLFDKRLSVGGGMWGGAMMFNEIVVQEEGKLILDEFDIKCSLFEPN
YYTTDSIEAITTLISKTVKAGVKIFNGIEIEDVVLKKVDGDYKVGGVVINWTTVNMAHLPVDPIVISSSFTVDATGHDAHLAETLVRKGGVKLNTDS
GAVIGEKPMWAQIGEQDTVNHTKEIFSGLYVCGMAANAVSGAHRMGPIFGGMLNSGKKCAQLILEKWSRK

>Desulfurella multipotens
MALDERIISKAIIERYSQKLLSQLDCDCVIVGGGPAGLICGYELAKNGLKVTLFDKRLSVGGGMWGGAMMFNEIVVQEDGKAILDEFDIKTVLYEPN
YYTADSIEAISTLISKTVKAGVKIFNGIEIEDVVLKKVDGQYRVGGVVINWTTVNMASLPVDPIVVSASFTVDATGHDAHLAQTLVRKAGVKLNTDS
GGVPGEKPMWADVGEQDTVNHTKEIYNGLYVCGMAANACSGAHRMGPIFGGMLNSGKKCASLILEKWGKK

>Desulfurobacterium atlanticum
MELSEVVISRAIVERFMNKLLSNLKVDVAIVGGGPSGLVAAYYLAKEGFKVSLFERKLSIGGGMWGGAMLFNEIVVQEMGREILDEFDVGYEKFQEG
YYTTDSVEAVTTIASKAVKAGAKVFNGVTVEDVVLKKENGDYRVCGLVINWTPVEITGMHVDPLTIESKFVIDATGHDAYVVSTLQKKAGIRLDTKT
GCVVGEKPLWASVGEEDTVKNSREVYPGIYVSGMAANAVCGSHRMGPVFGGMLMSGKKIAKEIAERLKHNVEE

>Desulfurobacterium indicum
MENLSEVKISKAIIERFTEKLLSNLEVDVAIVGGGPSGLVAAYYLAKEGLKVSLFERKLSIGGGMWAGAMFFNEIVVQEMGREILDEFSVSYRKYDE
GYYTADAVEAVTTIASKAMKAGAKIFNGVTAEDVVLKKVNGQYRVCGLVINWSTVDMTGLMVDPLVVTSNYVIDATGHDATIVSTLQKKAGIRLDTE
TGCVVGEKPLWASVGEEDTVKNSREVFPGIYVSGMAANATCGSHRMGPVFGGMLMSGKKIAMEIAQKLKS

>Desulfurococcales archaeon ex4484_42
MVKELESRVTELIVKHASRDWAELASTDVVIVGAGPSGLTAAKYLAEDGIKVVIFERRLSFGGGIGGGGMLFHKVVVEDFALDILKDFGIRYVEDGG
LYVVDASELMAKLAVGALNAGAKIIHGVTVEDVIFRTNPLRITGVAIQWSAVPLANLHVDPLLIYSKAVIDATGHDAEVVRVASRKIPELRLKVTTE
KSAYSELGERLVVEKTGKVVPGLYVTGMAVAALNNLPRMGPIFGGMLLSGKKVANEVLKDLRT

>Desulfuromonas sp. SDB
MKDINITNHIIAEFYKDIQDRVSSDVIIIGAGPSGLVASYLLAQDNFKVTVFEKRNQPGGGIWGGGMMFNQLVLPDDLQDFLNQMSIKFKLHPDNLI
SVDSVHFSSALLYHATEVGVKIFNNIGVEDLLVVDDMVRGVVINWNDVIKNKIPIDPLTFEAKAVVDSTGHPADGVEKLARRGLVEISQEFPMNADV
AEKFVVEATGQLYPGLYVSGMAATAAKGGPRMGPIFGGMIKSGIKIANLIKQTWRSS

>Dethiosulfatarculus sandiegensis
MMLDEVTITRAIIDRYMEKLHANLDLDVAIVGGGPSGLVAGYYLAKKGYNVAMFERKLSIGGGMWGGGMMMNEIVVQEEAKRILDEFGIPCREYVEG
YYTADSVVSTSTLTSKATLAGLSVFNLITVEDVMVRDNRVNGLVINWSPVEMAGLHVDPLTLRARYTIDATGHPAEVLNVISKKVDAKLSTDTGKVI
GERSLWAEVAESTTIENTKEAFPGVYTAGMCANAVFGAHRMGPVFGGMLLSGEKVAQVLDRKLQEED

>Dethiosulfovibrio peptidovorans DSM 11002
MELDERVISKAIVSRFFERLTDHLENDVVIVGGGPAGLVAGYVLADAGVKVSLFDRRLSLGGGMWGGGMLFNEIVVQSEGARILDDLGVSLREFEPG
YYTAGSVEAVSTLISSAVRAGVTVFNGMVAEDVVMREDRVIGLVINWSTVETSGLLVDPLAVRSDFIIDATGHDSNVTSTVEKKVPGRLLTETGKVE
GEKSLWCERAEKLTVDNTKEVYPGLFVAGMSANAVFGGPRMGPIFGGMLLSGEKAAKEILLRLNGKRVS

>Dissulfuribacter thermophilus
MREIDITKAIIDKHIEELNKCLCSDVVIVGAGPSGLVAGSILAQKGYTITIFEKRLAPGGGIWGGGMGFKYVIIQKEALDIVEEFNIPYEKYSDDLY
AVDAINFASGLILEAGKRGVHIFNLIAVEDLLVREGRVQGVVINNTFAKMNQFPIDPLTIEAKAVVDATGHEHEVVKTLSQKNDVTLNTPTGKPLGE
RSLFAETAEKAVVTNTKEVYPGLYVCGMATAAVYGGYRMGPIFGGMLMSGKKLAGLLEEALKA

>Elusimicrobia bacterium CG_4_10_14_0_8_um_filter_37_32
MKLDDIVISKAIMETFTKDFVDYLEVDVAIVGGGPAGLTAGYYLAKKGKKVVLFERKLSIGGGMWGGGMMYNKCVFQEDAKKILDEFGVTTHKYQEG
YYVTDSLETVSVLCSKAIKAGLKIFNLISVEDVMIRKEKITGLVLNWSAVQLAKLHVDPMTIRAKYVIDATGHDAEVVKIVVRKIGKKLYTKTGDML
GEKPMWAEVGEKDIIKNTKECYPGLYICGMASNAVFGGPRMGPIFGGMLLSGKRISGLVT

>Euryarchaeota archaeon ADurb.Bin165
MTLDEVTISRAIITDHLNTLVQYMEMDVAVIGAGPSGLVCATILAEKGLKVGLIEKKLSVGGGMWGGGMMFPRIVVQQGAKRLLDRFGIRSSEFSPG
YYTARSIEAVAKLAAAASDADVEFFNLTTVEDVMVKGDGLLSGLVINWQPVEATGLHVDPLTVRCRMTVDATGHDAIIAHYVSKKCGGLEIKGEGTM
WADNAEAAVVAHTKEVYPGLYVCGMAANAVMGGNRMGPVFGGMLLSGESAAEQILSRF

>Ferroglobus placidus (strain DSM 10642 / AEDII12DO)
MPFSEKNITRVIVREAAKEWEEISETDVVVVGAGPAGLTAAHYLADFGFDVVVFERRLSFGGGIGGGGMLFHKIVVEKEAKEIAEEFGIKTREVEDG
LYVIDAAEMLAKLSAGAIDSGAKVILGVTVDDVIYRPEPLRISGVLVQWSAVQIAGLHVDPLMIESKAVVDATGHDAEVVSVAARKIPELEIYVAGE
KSAYSELSEKLVVEKTGKVVDGLYVAGMAVSAVYGLPRMGPIFGGMLLSGRKVAEQIMFDLKK

>Fervidicoccus fontis
MSENLEFKITKLILEHSMKDLIEFADSDVIIVGAGPSGMTAAKYLADKKLKVLVLERKLSFGGGIGGGGNLMHKIVIKSDALKIIKDFEIEYKKTEF
EDLYTLDASELISKLATGAINSGAKILFGYSVEDLIVREKPLRVSGVVVKWSAIDLAQLHVDPIFFTGKAILDATGHDAELIKILAKKNPSFAINVK
NESSAHAELGEKQVVEFSGKVCDGLYAAGMSVATLHGLYRMGPIFSGMLISGKKVAELISKELGK

>Fervidobacterium changbaicum
MGKDLTISKLIVENFFEKLSNALEVDVAIAGCGPSALTLSLELSKKGYKVAIFEAKNEPGGGIWGGGMMFNEVVLESELEGYLKELGIRFKKFDEFI
VTDSVHLASALLYHTTLAGTMIFNNVFVEDLVMYDRRVSGVVINWTPTLREKLHVDPISIVSKFTVDGTGHPANLVKLLSKRGIISSIGGSTEASYN
FGIVGYEFPMDAENGERFVVENTREIYPGLYIVGMAAVSVGAGPRMGPIFGGMIMSGLRAAELISNELRKMGGSDDER

>Gemmatimonas sp. SG8_17
MRGRRGGGVTDFSEGQITRAIITAYHEKLWGQVVGDVVVVGAGPSGLVAATDLARRGLKVTVLEKRLSPGGGIWGGAMAMNEVVVQDAALPLLAEFS
VSFRSVKGGLHVINAVELASALSLKAVQTGAVILNLTVAEDVCVHRGRVTGVVANRTNLAEALPVDPVSFEAKAVLDATGHDAALVQMLQRRGLLKG
LTEMQGEGPMDAAGGESFVVDKVTEVYPGLWVSGMAVVATLGGPRMGPIFGGMLLSGKRAADLISDTLSGE

>Geoglobus acetivorans
MSYSERNITRIIVREAAKDWEDISDTDVVIVGAGPAGLTAAYYLREFGFDVVVFERRLSFGGGIGGGGMLFHKIVIEEEAKEIAEGFGMKLKEVESG
LYSVDSSDFLAKLSYSAVESGAKVLLGVTVDDVVFRPDPLRISGVLVQWSAVQISGLHVDPLMIESRAVVDATGHDAEVISIAARKIPELEIFIHGE
KSAYSEMSEKLVVEKTGKVADGLYAAGMAVAAVHGLPRMGPIFGGMLMSGKKVAEQIMFDLKK

>groundwater metagenome
MIDETKITELIVRSAVDDFLGNLKVDVVVVGGGPAGLTTARYLAKAKKRVVLFERKLSIGGGMWGGGMMFPRVVLQKGGKEILEECNVRYKKFDDLW
VADSIECVTKMTAKAIDEGVKIFNLISIEDVIIRSVQSNKNNKEGKTKICGVVLNWTAVQMANLHVDPLSVKSDFVVDATGHEASICHLVVKKVGNL
NTKTGDVLGERSMWAEKGESDIMNNTKEVYPGLFVSGMAANAVYGSERMGAIFGGMLMSGKKVSELILEKEK

>Hadesarchaea archaeon DG-33-1
MGGIEDTEITAAILKRFMRDFEDVTNLDVAIAGAGPSGITAASFLASGGAKVAVFERNLHVGGGMWGGGILFSRVVIQEAAKVMLEEVGVKLKPTAA
GYYTADSVEAVTKSTTAAVDAGARVMVGLTVEDVMIREKDRVAGIAVNWKAVELAGLHVDPVGISAKIVIDATGHDAMIARIVQRKVPNAKFPTSTG
GVVGEKPVWAEVGETEIVNNTREIYPGLIVTGMAANTVFGSPRMGPIFGGMLLSGRRAAEVALKV

>hydrocarbon metagenome
MWYLVELDERVISRAIIAVQMEKMLRYTDMDVAIVGGGPAGLTAASFLGAEGFSVALIEKKLSVGGGMWGGGMMFPRIVVQEEGRQLLDHFAIRYTR
YEEGYYVASSVEAVAKLTAAACDAGVEFFTLVTVEDVMVRSDKRLSGLVITWSPVEMAGLHVDPLTLGCRYTIDATGHDAVIARLVARKSGAVTVKG
EGFMWADRAESRITSHTREVFPGLIVAGMAANAVAGENRMGPVFGGMLLSGRHAAALVSRELASPKP

>Hyperthermus butylicus (strain DSM 5456 / JCM 9403 / PLM1-5)
MVNAVQAPEHSWLPHNVTSLREGALAALIIRKTAEKLTSITSVDVAIAGAGPAGLTAAWLLAEKGLRVVVVEHSLGVGGGMRGGSMLMPVGLVEDGL
PAELLRRAGARLDRVADGLYAVDPTEAVVKLAAKAIDAGAVILPGLHVEDLILWRSGSGYRVAGLVINLSPVVEAGWHVDPIYIEARATIDATGHDA
ELVKLLSKALGDSSIRVRGTRGMDVWEGEKLVVEYTGEVYPGLYAAGMAVSETYQLPRMGPVFGGMLASGARVAELVASRLSEQ

>Ignicoccus islandicus DSM 13165
MIDEGKVTSIIIEESSKELSQMAKGVDVVIVGAGPAGLTASHYLAKAGLKVLILERRVSLGGGISGGGSLFHKVVVEDVELEGYNPKEIAEELGVPL
KKVDDNLYTTDAAALVAKLSNASVSAGAKIVLGMHVEDLIYRIEEGVTKVKGVVALWSPIYLSGLHVDPIFFKAKAVVDATGHDAEILKIASKKLPN
VNFEVGREYGAWIDEAEKLVVKYTGKVLEGLYAAGMSVASFYRLPRMGPVFGGMLASGKKVAEKIIGDLEVS

>Ignisphaera aggregans (strain DSM 17230 / JCM 13409 / AQ1.S1)
MKELELRISRAILRNSVRELIEYSDVDVVIVGAGPSGLTAARYLAMNGFRVVVLERRLSFGGGIGGGGMLFHKIVVSSEALPILNDFDIKYYRDDEE
DLYMIDSSELMAKLAVGAINAGAKIFHGIHVEDVIYRENPLRITGVVIQWSAVVMSGLHVDPLFITSRAVVDATGHDAEVLQIVSRKIPEVGISLPG
ESSAYSELSEKIVVEKTGMVIPGLYVAGMAVAALYKLPRMGPIFSSMLLSGKRVAEIIANDLKKK

>Korarchaeum cryptofilum (strain OPF8)
MESLESRISKAIWESTYKDWLDIIDSDVVIVGAGPSGLTAASYLAKSGFKTTVIERRLSFGGGIGGGGMQLHKVVVDGRALKVLEDFKVRYSYLEKY
DLYVLDSAELMAKLASGAIDSGAKLIHGLTVEDLIVREDPFRVEGVVVQWSSVLLAGLHVDPLFIHSRVVVDATGHDAEVIRILERKNPSLGIKVPG
ERSAYSELSELSVVERTGKVVEGLYVTGMAVAALNQLHRMGPIFSGMLLSGRKVAEEIIRDLS

>Labilibaculum filiforme
MEQIVSAGIVDSYFKKLKENLSVDVAIVGGGPSGLVASYYLAKKGFKVALYESKLAPGGGMWGGAMMFNEIIVQKDALHILNELGVSYQHYQEDYYT
LDSVHATSALIYHATQAGVKIFNCSFIEDVVFQNDKVCGVVLNWSPVRREGLHVDPLVVMAKAVVDGTGHDCDIARTLERKNDVKLNTKTGKVMGEC
SLSIDEAERTTVENTKEIYPGLYVSGMASNGVSGGFRMGPIFGGMLRSGEKLAGLIAENLSK

>Latescibacteria bacterium DG_63
MKLDDVEISKAIIESFYAKLLDSLMCDVAVVGGGPAGLTAAYYLAKHGRKVVLFERKLSIGGGMWGGGIMFNEIVVQRDGKKILDEFGVRTTLVKEG
YFCADSVEAVSTICSKASQAGARIFNLFSVEDVMMTEERVTGLVINWSAVELSNLHVDPISIKAEHVIDATGHAAEVAHIIQTKSGSKLLTPTGTVI
GERPMCAEVAEKSILENTKEIFPGVLAAGMCCNAVFGAPRMGPIFGGMLMSGKKAAELIIDKPAAPKRRCLPDDE

>Lentisphaerae bacterium ADurb.Bin082
MAMENIITTAIIRQFADKLSAGTDLDVAFVGGGPSALVAAAKLAKKGLKTAIFEKSLAPGGGVWGGGMLFNEIVVQENVLGILEQIGISYQAVPDAK
GYYTVDSVEMASGLIFNAVKAGAKIFNAMSVEDIVFKEGRVNGLVINWAPVRKLAMPVDPLTVIAKAVVDATGHPCEIIRIACEKAQVKIATETGGV
LGERPMWVQHGEQQTVDSTAEYYPGLFACGMSATNVTGGYRMGPIFGGMILSGLKAADLIAKSLASLEKR

>Metallosphaera yellowstonensis MK1
MEIRQVDEVKITKYILKATFEDWMDIAENDVVIVGAGPSGLSAAYYLAKKGLKTTVFERRLSFGGGIGGGAMLFHKIVIESPADQVLREMNIRLQRV
EEGVYIVDSSEFMAKLASSAIDAGAKIVHGVTVDDVIFRENPLRVTGVAVEWTATQMASLHVDPLFIHAKAVVDATGHDAEVISVAARKIPELGIAI
PGEKSAYSEVAEKLTVDNTGEVAPGLYAAGMAVTEVKGLPRMGPIFGAMVLSGKKVAEDIASTLLMKARNT

>Methanobacteriales archaeon HGW-Methanobacteriales-1
MELDDITISRAIVEEFMNDFMDYMDIDVAIGGGGPAGLTAGYYLAKAGLKVALYERKLSIGGGMWGGGMMFNKIVVQEEGKRILDEFGIQSKKYQEN
YYVSDSVEATSTLCSKATQAGLKIFNLMSIEDVMIRGDDISGLVLNWSSVEMGGLHVDPLSIRSKAVIDATGHPCEVVKVVQNKIGPKLNTPTGEII
GEKSMWAEVGEPAIMENTREVYPNLYVAGMAANAVYGAPRMGPVFGGMLLSGEKIANMLIEKLK

>Methanobacterium subterraneum
MKLDDIIVSKGIVAGYMEELLDYMEMDVAIGGGGPSGLTAGYYLAKAGLKVALFEKKLSMGGGMWGGGMMFNKIVVQEEGKRILDEMGIRNQEYEEG
YYLADSVESASTICSKACQAGLKVFNLMEIEDVMIKGEGVEGLVINWSPVEMAGLHVDPITVGARAVIDATGHPCEVVKVLERKMEAPLKTETGKIM
GEKSMWADVAEQNIMGNVGEIYPGMYVTGMAANAVHGSPRMGPIFGGMLLSGEKVAEMLIEKLK

>Methanobrevibacter woesei
MKKLDDITVSKAIIQEYMNDFLDYTDMDVAIGGGGPSGVTAGYYLAKAGYKVALFERKLSIGGGMWGGGMMFNKVVVQEEGKRILDEFGIKSKKFED
NYYTVDSIECTSTLCSKATQAGLKIFNLMSIEDLMVRENGINGIVLNWSSVEMSGLHIDPLTVRAKAVIDATGHPTEITKIVEQKMGANLKTETGKI
MGEKSMWADRAEGKILDNVTEVYPGLWVTGMAANAVHGSQRMGPIFGGMLLSGEYVAQKIIEKLENE

>Methanocalculus sp. 52_23
MQLDEVTISRAILETHAEISSRYLDLDIAIVGGGPSGLVCAALAAEDGRKVAVIEKKLSVGGGMWGGGMTFPRIVVQEEGKRLLDQFGIRSRVYKPG
YHVASSVESVAKLTAAACDAGAEFFNLTSVEDVVIKEDGRVSGLVITTSPVEMTGLHVDPLTLAAKVTVDATGHDAVVAHCVLRKGGDITIHGESFM
WAERAETNIINHTREIFPGLIACGMAANAVAGEARMGPVFGGMLLSGEHAAVLAREISERV

>Methanocella conradii (strain DSM 24694 / JCM 17849 / CGMCC 1.5162 / HZ254)
MELDETLISRAIIDDFLRTLSDYVSVDVGIVGGGPSGLVCATYLARAGVKVAVFERKLSVGGGMWGGGMMFPRIVVQQEATRILDDFGIRYREYRPG
YYIAGSIEAVGRLTSAAAGAGAEIFNLMSVEDVMIRENKEVVGLVINWSAVDIAGLHVDPLTVRTRVVVDATGHPAEVCRIVERKVSGGAFKVPGEQ
SMWADRGERALISTTKEVYPGLVVAGMAANAVAGGPRMGPIFGGMLLSGEIAARIVKEKLGVS

>Methanococcoides burtonii (strain DSM 6242 / NBRC 107633 / OCM 468 / ACE-M)
MKLDEVTISRAIIEEFSKVFLDYTDVDVALVGGGPANLVAAKYLAEAGLKTVIYEKKLAVGGGMWAGGMMFPRIVVQEDALHILDEFGISYHEYENG
YYVANSIESVGKLISGATSAGAEIFNLVNVEDVMIRENDEICGLVINWTAVEIGKLHVDPLAIRSKVVVDGTGHPAVVCSTVQRKVPGAKLGELGVV
GEKPMWADVGEKMLLDTTKEVYPNLYVAGMAANAVAGAPRMGPVFGGMLLSGKQVAELIIERLG

>Methanococcoides methylutens MM1
MKLDEVTISRAIIDEFSKVFLDYTEVDVALVGGGPANLVAAKYLAEAGLKTVIYEKKLSVGGGMWAGGMMFPRIVVQEEARHILDDFGIDYHEYEEG
YYIANSVESVGKLISAATSAGTEIFNLVNVEDVMIRDNDEVCGLVINWTAVEIGRLHVDPLAIRAKVVVDGTGHEAAVCNTVQRKVPGAKLGELGVV
GEKPMWADVGERMLVETTREVYPNLYVDGMAANAVAGAPRMGPVFGGMLISGKQVADLIIERLK

>Methanococcoides vulcani
MKLDEVTISRAIIDEFSKVFLDYTEVDVALVGGGPANLVAAKYLAEAGLKTVIYEKKLSIGGGMWAGGMMFPRIVVQEEARHILDDFDITYHEYEKG
YYIANSVESVGKLISGATTAGTEIFNLVNVEDVMIRENDEVCGLVINWTAVEIGRLHVDPLAIRAKVVVDGTGHEAAVCNTVQRKVPGAKLGDLGVV
GEKPMWADVGERMLLETTKEVYPNLYVDGMAANAVAGAPRMGPVFGGMLLSGKQVAELIIERLK

>Methanococcus maripaludis
MDGKLRADEVAVTKSILKSTFDMWMDLIDVDVVIVGAGPSGLTAAKYLAQNGVKTVVLERHLSFGGGTWGGGMGFPNIVVEKPADEILREAGIKLDE
VIGEPELFTADSVEVPAKLGVAAIDAGAKILTGIVVEDLILKEDKVSGVVIQSYSIEKAGLHVDPITISAKYVIDSTGHDSSVIHTLARKNKDLGIE
VPGEKSMWADKGENSLTRNTREVFPGLYVCGMAANAYHAGYRMGAIFGGMYLSGKKCAELILEKLENK

>Methanocorpusculum labreanum (strain ATCC 43576 / DSM 4855 / Z)
MDLEVTKAITESWFARLQENLCFDAAIVGTGPSGLIAAVKLADAGYKVSMFESKLAPGGGMWGGAMLFSSIAVQNEAVYLLDELEIPYKRYNENLVV
CDSVLATSALIYQASKRGVVIHNGMSVEDVVFMDNRVSGVVVNWGPVVREGLHVDPLSFRAKIVVDATGHPCMISETAARKNNITLNTPTGKVCGEC
SLNAVEGEAMTVENTKEIYPGLYVCGMAANGVFGSPRMGPIFGGMLLSGEKVAKLIIEELK

>Methanoculleus thermophilus
MTLNEVTISRAILEESHRALIEHLEMDVAVVGGGPSGLACAALLGEKGLSCALIEKKLSIGGGMWGGGMMFPRIVVQEEARRLLDRFGIAYKEFEPG
YYVAKSVEAVAKLTAAACDAGVEFFNLTTVEDVMIRGDGRVGGLVINWTPVDMAGLHVDPLTVACTCTVDASGHDAVVARMIERKGGRLQVKGESFM
WAERAESRILDHTKEVFPGLFVAGMAANAVAGECRMGPIFGGMLLSGERAAELVAESLER

>Methanohalobium evestigatum (strain ATCC BAA-1072 / DSM 3721 / NBRC 107634 / OCM 161 / Z-7303)
MELDDITITKAIVDDFSKTFIDYTEVDVALVGGGPANMIAATRLAQEGYKVALFEKKLALGGGMWGGGMMFPRIVVQDEARKILEEFDINHYEYDNE
KGYYIANSIESVSRLINKTVTSGVQVFNLVNFEDVMIREDDRVTGIVINWTAVSIANLHVDPLTIRAKVVIDGTGHEAVVCNTVQRKIPNAKFEGVV
GERPMWADAGEKSLKETTREVYPGLIVTGMAANAVAGAPRMGPVFGGMLLSGEMAAKIAMSKLD

>Methanohalophilus euhalobius
MELDERIITRAIVEEFTNVFLDYTDVDVALVGGGPANLVAARYLAEAGLKTVLFEKKLSVGGGMWGGGMMFPRIVVQEEARRILDDFDVPYHEYEEG
YYVANSVGTVGKLISAAVSAGVEIFNLVSFEDVMIRDNDEVCGLVINWTAVEIARLHVDPLTIRSRLVLDGTGHEATVCNTVQRKIPGAFGGKEVVG
EKPMWADTGERLVMKNTREVYPGLIVTGMAANAVAGSPRMGPVFGGMLLSGEKAAQLAISRLKD

>Methanolacinia petrolearia (strain DSM 11571 / OCM 486 / SEBR 4847)
MKLDEVTISRAILSEQHKIMTEYLDIDCAVVGGGPSGITCAAILAQNGVKVALIEKKLSIGGGMWGGGMMFPRIVVQEEARRLLDHFGIKYTEYEKG
YYVASSVEAVSKSLAAACDAGAEVFNLTTVEDVVVKEDGGVSGLVINWTAVEMAGLHIDPLTMRTKVTVDATGHDSMIAHMVRKKGGALEIKGEGFM
WAERAETNILSHTKEVFPGLIVAGMAANAVGGETRMGPIFGGMLLSGEKAANMIIERLKK

>Methanolinea sp. SDB
MELSETTITRAIVSSQMKILLEYSELDVAVVGAGPSGLTAAAILGDAGYKVGVIEKKLSVGGGMWGGGMMFPRIVVQEPARRLLDRFEISYQPFEEG
YYVASSIEAVARLTSAACRGGAEFFNLTSVEDVMVKDDGRVSGLVINWTPVEMAGLHVDPLTIGCRYTIDATGHDAVVATLVERKGRNLEVKGEGFM
WADRAESEIISHTREVYPGLIVTGMAANAVAGEHRMGPVFGGMLLSGEFAASLVREKLNR

>Methanolobus profundi
MELDETIITRAIVEEYSKVFLDYIEVDVALVGGGPANLVAAKYLGEAGLKTVLFEKKLSIGGGMWGGGMMFPRIVVQEDAKHILDDFNINYHEYEKG
YYVASSIESVGKLICGATDAGAEIFNLIDVEDVMIRENDTVCGLVINWGPVSMNRLHVDPLAIRAKVVIDGTGHDAGICSTVQRKIPGTDIKLDVVG
EKPMWADVGEKILMDTTKEVYPGLIVTGMAANAVAGAPRMGPVFGGMMLSGKKAAELAIEKLRK

>Methanomassiliicoccales archaeon PtaB.Bin215
MEIDEVLVTRKIVERYTEEFLENVDVDVVIAGAGPSSLTAARYLAKAGLRVVIFERKLTPGGGMWGGGMTFPIIVVQEGSKDLLGEIGVRLRDAGDG
YFTADSVEASAKLISAAVTAGARLYNTISVEDVMIRQDSICGVVINSSAVEVAGLHVDPLAVRSKYVIDGTGHPAEVVHVVQRKVGRLNTPTGQIEG
EKSMWAEVGEQMTVENTVEVYNNLYVCGMASNAVMGAPRMGPIFGGMLLSGRKVAEMIIAREKKKGKKK

>Methanonatronarchaeum thermophilum
MNVDDKFVSKAIIDEFSKDFLDSLSVDVAIGGAGPAGMVAAKYLAENDIKTAVFERKLSVGGGMWGGGMMFPRIVIKEKSLPILDDLNINYREYQDG
YYIANSIESVGQTAAEAVKAGAEIYNLMTVEDLHYKENKVNGVVINWSSVDLAGLHVDPLTIESKITIDATGHDCELVKVAQERINKKLNTKTGKIM
GEKSMWAEQGEKDVVELTGEVLPGLYVTGMAVNAVHGKPRMGPIFEGMLLSGKKVAEQCIKKLK

>Methanoplanus limicola DSM 2279
MKTKVYESENKMKLDEVAISRAIVSEQSKVMLDYYDLDCAIVGAGPSGLTCAAMLGEEGLKVGVIEKKLSVGGGMWGGGMTFPRIVVQEEARRLLDH
FGIKYREYESGYFVSSSVEAVAKITSAACDAGAEFFNLTYVEDVVIKGDNRISGLVINQTPIQMTGLHIDPLTLATKVTIDATGHDSVVAHLVRDKG
GSVEIKGEGFMWADRAESNILSHTKEIFPGLIVTGMAANAVGGETRMGPVFGGMLLSGEKAAKLAKSALKK

>Methanopyrus kandleri (strain AV19 / DSM 6324 / JCM 9639 / NBRC 100938)
MEREITPIVLREGYEFINDCSESDVIVVGAGPAGLTCAYELAKSDVDVTIVERKLYVGGGMTGGGMLFPAGVIMEETAEVLEEVGVELRPAEAGLLA
FNPVEAAIKLANAALEAGARILVGIEVEDVIERRGRVCGVVVNWTAVKAANMHVDPLALEAEYTVDATGHEAAVCKLAGIEVKGEGPMWAERGEELV
VKHTQEVKPGLFVAGMAASAVKGAYRMGPIFGGMLESGKKAAEEILERLTE

>Methanoregula formicica (strain DSM 22288 / NBRC 105244 / SMSP)
MELDELTISRAILASQTNVLINHLELDAAVVGGGPAGLTCAALIAGQGKKVGVIEKKLSVGGGMWGGGMMFPRIVVQEEARRLLDLFGIRYTPFESG
YYVARSVEAVSKLTAAACDAGVEFFNLMSVEDVMIKADKRISGLVINWTAVEMGKLHVDPLVMGSRYTVDATGHDAVVARLVEKKGGDIRVKGEGFM
WADRAETNILNHTKEIFPGLVVAGMAANAVAGESRMGPVFGGMFLSGERAAQIVLREMKA

```
>Methanoregulaceae archaeon PtaB.Bin009
MELDEITISRAILSSQVEKLLEFMEMDVAVVGGGPSGLTAAALIGEQGFRVGLIEKKLSVGGGMWGGGMMFPRIVVQEEAKRLLDQFDIAHTSYTEG
YYVASSVEAVSKLTASACDAGVEFFNLFSVEDVMIRGDSRLSGLVVNWTPVEMAGLHVDPLTMGCRVAVDATGHDAVLARLVERKGGDVKVRGEGFM
WADRAESEIVSHTREVFPGLVVCGMAANAVAGEHRMGPVFGGMLLSGERAAALATSSLRQENSAA

>Methanosaeta harundinacea
MALDEVTITKAIVESYMESFLKYTDVDVALVGAGPANLVAAKKLAEADAKTVVFERNLSVGGGIWGGGMMFPRIVVQKEGCRILDEFGVWYREYEEG
YYIASSIETVAKLTAGVIDAGAEIINLVTVEDVMIREDERIAGLVINWEAVERTRLHVDPLSVRARVVIDGTGHDANICKVVQRKIPGAKVGSLGVV
GEKPMWADVGEKTVVEVTQEVYPGLIATGMAAAAVAGGPRMGPIFGGMLLSGEKAAMLALEKLGL

>Methanosalsum zhilinae (strain DSM 4017 / NBRC 107636 / OCM 62 / WeN5)
MELDEVVITRAIVDEFLNVFLDYTDVDVALAGGGPANLVAAKYLAEAGYKTVLFEKKLSIGGGMWGGGMMFPRIVVQEEARRILDDFNITYKEYEDG
YYVANSIESVSKLAAGATSAGAEIFNLVSVEDVMIRENDRVSGLVINWTAVGIGKLHVDPLTIRSKVVIDGTGHDASVCNIVQQKVPGAQLGELGVV
GEKPMWADVGEKLLMETTREIYPGLIVSGMAANAAAGAPRMGPVFGGMLLSGEKAAELAISKLD

>Methanosarcina acetivorans (strain ATCC 35395 / DSM 2834 / JCM 12185 / C2A)
MELDEVIITRAIFDEYSKTFLDYTDIDVALVGGGPANLVAAKYLAEAGVKVALYEQKLSLGGGMWAGGMMFPRIVVQEEATRILDDFGIRYKEYESG
YYVANSVESVGKLIAGATSAGAEVFNLVSFEDIMIRENDRVTGIVINWGPVTTQRLHVDPLMIRTKLVIDGTGHEAVVCNTILRKIPNAKIGELGLL
GEKPMWSEVGERLAVNATQEIYPGLIVAGMAANAATRAPRMGPVFGGMLLSGEKAAKLALDRLKTI

>Methanospirillum stamsii
MTLDEITISRAIISDYMHTLLEYMEMDVAIVGGGPSGLVCSALIAEKGYKVGLIEKKLSIGGGMWGGGMMFPRIVVQSEAKRLLERFNITHSEFSPG
YYTARSIEAVSKLTTAAVDAGVEFFNLTTVEDVMVKGDGRLSGLVINWQPVEATGLHVDPLTIRCRMIVDATGHDAVIAHYVSKKMGKPDIKGEGTM
WADNAESAVVTHTKEVFPGLFVCGMAANAVSGGHRMGPVFGGMFLSGESAAVQILQQL

>Methanothermobacter defluvii
MEIHAGVKMKLDDIKISRAIVEGYMEDLLDYMEMDVAIGGGGPSGLTAGYYLARAGLKVALFERKLSIGGGMWGGGMMFNKIVVQDEGREILDEFGI
RSEPYDEGYHVADSVEATSTLCSRACQAGLKIFNLMSIEDVMIRDEGITGLVLNWSSVEMAGLHVDPLTVRAGAVIDATGHDCEIVKVVERKIGPEL
NTPDGRIQGERSMWADVGEAALIENTREVYPNLYVAGMASNAVYGAPRMGPIFGGMLVSGRRVAEMIIEKLK

>Methanothermococcus okinawensis (strain DSM 14208 / JCM 11175 / IH1)
MDKFKIEEKDVTTSILKATFNMWMDIVDVDVVIVGAGPSGLTAARYLAKEGVKVVVVERHLSFGGGTWGGGMGHPYITVQKPADEILREVGVKLEEI
DGGLYVADSVEVPAKLGVGAIDAGVKILTGVIVEDLILKENKVSGVVINSYAIDKAGLHIDPLTINAKYVIDATGHDASVTNTLARKNKDLGLEVPG
EKSLWAEKAENSILRHTREIFPGLFVCGMAANATHGGYRMGAIFGGMYLSGKKVAELILEKLKNND

>Methanothermus fervidus (strain ATCC 43054 / DSM 2088 / JCM 10308 / V24 S)
MVLNEVTISKAIISKYMEELIDNTNLDVAIAGGGPSGITAGYYLAKEGFKVALFEKRVSIGGGAWGGGMMFNKIVVQEEGKKILDEFDVNTERYENN
YYVADAIEMITTLASKACKSGLKIFNLINIEDIVIKNKKISGIVVNWTAAEMAKIHVDPLVIKSKFVIDATGHDCEVVKAVEKKLGPVLNTETGRIV
GEKPMWAEKGEKAVIKNTGEVYPNLYVAGMAANSVYGSYRMGPIFGGMLLSGKKVAELIRERLL

>Methanothrix soehngenii (strain ATCC 5969 / DSM 3671 / JCM 10134 / NBRC 103675 / OCM 69 / GP-6)
MSLDEVMVTKAIVEGYLESFLENTEVEAALVGAGPANLVAAKRLAEANIKTVLFEKRLSVGGGLWGGGMMFPRIVVQQAAIRILEEYGIRYHEHCKG
YYVANSIETVAKLTARAIDAGAQIVNLVTVEDVMIREQDRVVGLVINWTAAEMAQIHVDPLCIRARYVIDGTGHEASVCRVVARKIPGAIIGIDGVK
GEKPMWAEVGERTVVEMTQEVYPGLVVAGMAAAAVCGGPRMGPIFGGMLQSGEKAAGIVIENLNK

>Methanothrix thermoacetophila (strain DSM 6194 / JCM 14653 / NBRC 101360 / PT)
MALDEVKITRAIVESYLESFLKCTDVDVALVGAGPANLVAAKRLAEADVRVVLFEKRLSVGGGLWGGGMMFPRIVVQKEACRILDEYDIWYREFEEG
YYVADSIEVVAKLTAGAIDAGAELINLVSVEDVMIREGDRIVGLVINWTAADMAGIHVDPLAIRARVVIDGTGHDAAVCRVVQKKIPGAIVGESGVI
GEKPMWAALGEKIVVDATREVYPGLIVAGMAATTVAAGPRMGPIFGGMLLSGEKAASIALEKLAQSVD

>Methanotorris formicicus Mc-S-70
MDLRLKADEYTTTKAILKSAFNMWMDIIDVDVAIVGGGPSGLTAARYIAKKGYKVVVLERHLAFGGGTWGGGMGFPYIVVEEPADEILREVGIKLEK
VDGEEGLYTADSVEVPAKLAVGSIDAGAKILTGIVVEDLILRENRVAGVVINSYAIEKAGLHIDPITITAKYVVDATGHDASVATTLSRKNPELGLE
VPGEKSMWAEKGENALLRNTREVYPGLFVCGMAANATYGGNRMGAIFGGMYLSGKKCAEMVVEKLKNNE

>Nitrospira bacterium SM23_35
MELDEVVITKAIVDQFCKKLTNHLETDVAIVGGGPSGLVAGYFLAKAGRKTVLFERKLSVGGGMWGGGMLFNEIVVQKAAVRILKEFGITYCEFQKN
YYTADSVESISTLISRAVQAGVTIFNCITAEDVLMRTSRVTGLVLNWSAVEMARLHVDPLAVRSRFVVDATGHETAVVRLVQNKVPGTLKTLSGKVE
GEKSMWSDKAESLTLKNTREVFPGLYVAGMAANATFGGPRMGPIFGGMLLSGEKVAKLLLQALSQKSK

>Nitrospira japonica
MGKPKPAPLRERDITRQIAREYYKEFDQLIESDVIIVGAGPSGLICAHDLADMGFKTVIVEQNLALGGGFWHGGYLMNKATICAPAHKILDEIDVPC
KRIKDCEGMYIVDPPHATGALIAAAYRAGAKVLNLTRVVDLILRRDGSLDGVVVNNTTAEMAGHDLIHVDPIALESKIVVDATGHDAVVVSLLHKRS
LYTEVPGNGAMWVSRSEEDVMDHTGEVYPNCFVIGLAVSAVHGTPRMGPAFGSMLLSGRYGAELIKKKLKQE

>Nitrospiraceae bacterium
MNPINVAPLRERDITRHIAREYYKEFDSLIESDIIIVGGGPSGLLCARDLATSGFRTLLIEQSLALGGGFWSGGFLMNKATICEPADQILEELGIPF
KPIKDCPGMTMVDPPHVTSRLISAAYEAGVKIMNLTKVVDLILRQDHRIEGVVVNNSTVEMAGHDTIHVDPIALESQIVVDATGHDAVVVNLLHKRN
LYQKVPGNGAMWVARSEALVVENTREIYPNCFVAGLAVAAVDGSPRMGPAFGSMLLSGRYAAELVRQKLKGE
```

>Nitrospirae bacterium
MPKPPTPAPLRERDITRHIAREYYKEFDQLIESDVIIVGAGPSGLICAHDLAAMGFKTVVVEQSLSLGGGFWSGGYLMNKATICEPANEILEEIGVP
CKKITECAGMYMVDPPHATGALIAAAYRGGAKIMNLTKVVDLIIRRDGILEGVVVNSTTAEMAGHDAIHVDPIALESKIVVDATGHDAIVVELLHKR
NLHKAVPGNGAMWVAQSEQEIMDRTGEVYPNCFVIGLAVAAVYGTPRMGPAFGSMLLSGRYGADLIKKKLKGA

>Omnitrophica bacterium RBG_13_46_9
MDEALISRAITESFTKDFIDAFNVDVAIAGAGPSGLICAYYLAKQNVKVAVFERHLRVGGGMPGGGMMFNRIVVQEEAMPILKEFGVSAKRYKKDLY
IVDALEAISTFCSKTIKRGAKIFNLINVEDVVIRKDRIAGVVLNWSAVSWAKLHVDPMAVRSKAVVDATGHDSEIARIVERKTGPVLRTETGGVIGE
KSMWAEIGEKMILENTKEIYPGLIVCGMAANAVFGSPRMGAIFGGMLLSGKKAAEVARKVIKSK

>Omnitrophica WOR_2 bacterium RIFCSPHIGHO2_02_FULL_68_15
MFARAQEAQITRAIVRAFAKEFDGLVRSDVLIVGAGPSGLVAAMDLARRGRRVLVVEQTNYLGGGLWLGGYLFNKLTVRAPAHRLLKELKVPCRQVQ
PGLYVADAPHVCARLIAAACDAGVKFAQMTEMVDVVVREGGRVEGLVINWSPVSALPKGLAHVDPVALEAKVVVDATGHDAAVVRLLAKRGLAAPVP
GDGAMWVERGEQAVMDKTGEVHPGLFAAGLAVSAVHGTPRMGPAFGAMLLSGRRCAQMIERAYFA

>Peptococcaceae bacterium SCADC1_2_3
MPLEDITISKAIITRYNQELLAALESDVAIAGGGPSGLVAAFYLAQQGAQVVLFERNLSLGGGMWGGGMMFNQIVVQEEAIPILDTFGVRYRTFEPG
YYTAHATETVAAIILGAVRKGVKILNLISAEDVMVRNERVCGLVLNWTAVGLARLHVDPIAASCSCVIDCTGHDAQIANIVVRKMGAVLKTSSGKIE
GEKPMWAERGEAAIIKNTGEIYPGLYVAGMAANAVYGNHRMGPVFGGMLLSGKRAAELIKGER

>Phorcysia thermohydrogeniphila
MQNLNEVIISQAIIESFMEKLKNSLEVDVAIVGGGPSGLVAGYYLAREGFKVSIYERHLAIGGGMWAGGMLFNEIVVQEMGREVLDEFGVRYREFQP
GYYVADSVEAVTTIASKAVKAGAVIFNGVTAEDVVLKKVNDEYRVCGLVINWTSVERSRLPVDPLVITAKYVIDATGHDASVVSTLQKKAGIKLATE
TGCVIGEKPLWASVGEEDTVKNTREVFPGIFVSGMAANATCGSHRMGPVFGGMLVSGKKAAQEIAEKLKGNKEE

>Planctomycetes bacterium DG_20
MDDFDETDVSQAILRAYYAKVADALQGDVLVVGAGPSGLVAAWRLAQAGHRVVVLEKRLSPGGGIWGGSLGMNEVAVQKHALAILDEAGVRHQPSGR
LFTADAMELASALCLKALHAGAVILNLMTAQDVCVRSGRVTGVVANRSLLGESLPIDPIVFSARAAIDATGHEAVLANCIQRRGLLKNSLGRLPGEG
PLDAPAGERFVVDHVAELYPGLWTTGMSVCASLGGPRMGPIFGGMLLSGEKVAALVGQALKKTRPQVRHE

>Porphyromonas sp. CAG:1061
MEKLVSQGIITRYFEKLNDCLDLDVAIVGGGPSGIVAAYYLAKAGLKVAQFDRKLSPGGGMWGGAMMFNEIVIQEEALEIIKEMGINYEPYQDKLYT
MDSVESTAALLYNAVHAGARIFNCYSVEDVVYKENRVSGVVVNWTPVLREGMHVDPLNIMAKYVIDGTGHDSEICRVVAKKNGATLNTSTGGVVGEQ
SLDVITGEKMVVEGTKEIYPGLYVCGMASSAVYGTPRMGPIFGGMLMSGKKVANLIIDQLK

>Prevotella amnii
MIEKEISKGIITTYFEKMEKSLDLDVAIVGGGPSGIVAAYYIAKAGLKVALFDRKLSPGGGMWGGAMMFNQIVIQKEALDIIKEFEINYEQYSDNLF
TTDSIECTAAILYKAVHAGATIFNCYSVEDVVFKNNIVSGVVVNWTPVLREGLHVDPLNIMAKFVIDGTGHDSEICKVVARKNNITLNTSTGKVVGE
RSLDVIEGEQQVVEGSKEIYPGLYVCGMASSAVGGTPRMGPIFGGMLMSGKKVADMLIKRIQS

>Prevotella nigrescens
MIEKKISKGIISTYFAKMEKCLELDVAIVGGGPSGIAAAYYMAKAGLKVALFDRKLSPGGGMWGGAMMFNQLVVQEEALEIIKDFDINYEPYEDGLY
TADSVESTSALLYKATHAGATIFNCYSVEDVVFKNNIVSGVVVNWTPVLREGLHVDPLNIMAKFVVDGTGHDSEMCQVVARKNGIKLNTATGDVIGE
RSLDVAEGERQVVEGTKEIYPGLYVCGMASSAVGGTPRMGPIFGGMLLSGKKVAEAIIERLK

>Prevotella stercorea DSM 18206
MIETQVSKGIITTYFDKLQNNLDLDVAIVGGGPSGIVAAYYMAKAGLKVAQFDRKLSPGGGMWGGAMMFNQIVIQEEAMHIVKDFDINYQAFEDGLY
TIDSVESTSSLLYHAVHAGATIFNCYSVEDVVFKNNVVSGVVVNWTPVLREGLHVDPLNIMAKCVIDGTGHDSEMCKVVARKNGIQLDTATGGVIGE
RSLDVVEGERMVVEGTREVYPGLYVCGMASSAVAGTPRMGPIFGGMMLSGKKVADMIIEKLKK

>Prosthecochloris sp. ZM
MEEKISKFIIQSFFAKLEDSLTVDVAIVGAGPSGLIAAKELAKAGKKVAIFESKLAPGGGVWGGGMLFNEIVLQENIIPILDEYAIRYKTTGEGYVT
ADAVEVSSALIYGAVHAGVRIFNAVRVEDLAMRDERVCGVVINWNPVSRLEMHVDPLVITSRAVLDGTGHPSELINLASNKAGITLDTPTGKVMGEK
PMWMENGESSTVINTKRLYPGLYASGMAANNAMGGFRMGPIFGGMFLSGKKVAGLILEDIQG

>Pseudothermotoga lettingae (strain ATCC BAA-301 / DSM 14385 / NBRC 107922 / TMO)
MKDTMISTLIVNRYFKKLRSFLELDVAIVGAGPSGLTAAYELAKKGFKVAIFEEKNTPGGGIWGGGMMFNEIVLEKELEDFLNELGITYVIQENHVL
VDSVHFASALLYRTTMVGATVFNNISVEDVAMQDGKVCGVVINWGPTMRLGLHVDPITVKASFVIDGTGHPANVASLLAKRGLIEMKMELPMNADEA
EQFVVENTGEIFPGLMASGMAACAVHGGPRMGPIFGGMILSGKKIAQIIEEKLR

>Pyrobaculum aerophilum
MELKIGRAIIRHALKDLDEYSDVDVAIVGAGPAGLTAAKYLAEKGLKVVVYERRFSFGGGIGPGGNMLPKIVVQEEAVPILRDFKVRYKPAEDGLYT
VDPAELIAKLAAGAVDAGAKIILGVHVDDVIFRGDPPRVTGLLWIWTPIQMSGMHVDPLYTQAKAVIDATGHDAEVVSVAARKVPELGIQVVGEKSA
WSEVSEKLVVEHTGRVAPGLYVAGIAVCAVYGLPRMGPIFGGMLMSGKKVAEVVYKDLMAEAHAVRA

>Pyrococcus abyssi (strain GE5 / Orsay)
MLREVTISRAIIESYYRDLLNNLELDVAIVGAGPSGMVAAYYLAKGGAKVAIFEKKLSIGGGIWGGGMGFNKVVVQEEAREILDEFDIRYEEFEKGY
YVADAIEVATTIASKTVKAGVKIFNMIEVEDLVVKDNRVSGIVINWTPVLMTGLHVDPLTVEAKYVIDSTGHGAQVAQFLLKRGLIERIPGEGAMWA
EQGERLTVENTREVFPGLYVTGMAANAIAGAPRMGPIFGGMFLSGKKAAQEILEKLNL

>Rikenella microfusus
MEKLVSLGIVENYFEKLKNNLSVDAAIVGGGPSGLVAAYYLAKAGKRVVLYERKLAPGGGMWGGAMMFNDIIVQQEALPILDELGVCYKPYREGACV
VDSVHATSALVYAATKAGATIFNCYSVEDVIFRDEAVAGLVVNWAPVMREGMHVDPLMVTAKTVLEGTGHDCMIARLVARKNNVRLNTPTGEVAGER
SLNVEQGERLTVENTKEIYPGLFVSGMAANGVSGSFRMGPIFGGMLMSGKKAAELMIAKING

>Saccharolobus solfataricus (strain 98/2)
MEVKIKQVDEVKISRYIIKETMEDWYQFVESDVVIVGAGPSGLSAAYYLAKAGLKTLVFERRLSFGGGIGGGAMLFHKLIIEKPADEILREVNIRLK
EVEEGVYVVDSAEFMAKLATAAIDAGAKIIHGVTVDDVIFRENPLRVAGVAVEWTATQMASLHVDPIFISAKAVVDATGHDAEVISVAARKIPELGI
VIPGEKSAYSERAEELTVINTGKVAEGLYATGMAVTEVKGLPRMGPIFGAMVLSGKAVAGEITKDLLKSEIRA

>Smithella sp. SDB
MLNETTISRAILDAYFKKLDSCLELDVAIVGGGPSGLVAGYYLAKAGRKVALFERRLSIGGGIWGGGMMFNAIVVQEAGRQLLEEFDLKGSEYAPGY
YVLDAVDVTATLIHKAVRAGLQVFNLIAMEDVVIKNERVAGLVINWGAVDTLKWHVDPLTIHARYVIDGTGHPANVTEVLVRKMGVRLNTSTGGIVG
EKSMEAEQGELQTVENTREVYPGLYVSGMAANAVFGGYRMGPVFGGMLLSGRKAAEEILAHL

>Spirochaetes bacterium ADurb.Bin215
MLDETVISRAIIETYMKKLTDNLSVDVAIVGAGPSGFVAGYFLAKAGRKVVIFERALAVGGGMWGGGMGFNEIVVQEEGKAVLDEFDLPAVRYVQGY
YTLDSVRAVSALALRAVEAGVTVFNLVGVEDVVLHDERVSGLVLNWGTVMKAGIPVDPLTVHSRCVLDATGHPAHVAEVLCRKMGVSLNTPTGKMMG
EMSMDAEKGEKQTVENTREAYPGLFVSGMAANAVFGGYRMGPVFGGMLLSGKRAAEVMLARLGER

>Staphylothermus hellenicus (strain DSM 12710 / JCM 10830 / BK20S6-10-b1 / P8)
MKFFPQNLYELSEGDLSKTLIDALYKKLSEIVKVDVAIVGAGPSGLTAAWKLGEKGYKVLVLERMLGVGGGMRGGSMLLPVGLIEDGEAAEIAREAG
ARINKIRNGLFVVDPSELAVRLASKAIENGAIIWPGVLVEDLITRGRGEDLVVKGVLINWTPIYEAGWHVDPFYIEANAVVDATGHDGSLLRVLAKR
HPELKINIPGMSSQNVWIGEEMVVEKTSMVVKGLFVTGMSVAELYNTNRMGAIFGGMLVSGRKVADLIDDYFGKTRTLREQ

>Sulfolobales archaeon SCGC AB-777_J03
MASVRRVPESKISRFIVEETMKDWMDIVESDVVIVGAGPSGMTAAYYLAKAGLKTVVFERRLGFGGGIGGGAMQFHRLVIEEPADEVLREFGVRLKK
VDEGVYVVDAAEFMAKLASKAIDAGAKIILGVTVDDVIFREDPPRVAGVAVEWTATQMSGLHVDPLFISAKAVVDATGHDAEVISVAARKLPELNIS
VPGEKSAYSEVAEQLVVDNTGPVAPGLYAAGMAVCEVKSLPRMGPIFGAMVLSGKRVAELIIQDLRKS

>Sulfolobus acidocaldarius
MSDSIKIKAIDEVKISRYIIKQTMEDWMNFVENDVVIVGAGPAGMSAAYYLAKHGLKTLVFERRLSFGGGIGGGAMLFHKLVIESPADEVLKEMNIR
LEKVEDGVYIVDSAEFMAKLAASAIDAGAKIIHGVTVDDVIFRENPLRVAGVAVEWTATQMAGLHVDPVFISAKAVVDATGHDAEVVAVASRKIPEL
GIVIPGERSAYSEMAEKLTVEQTGVVAPGLYVAGMSVTEVRGLPRMGPIFGSMVLSGKKVAEDIIKDLRNS

>Synergistaceae bacterium
MRLDEVTITKAIMERYFDKFMNNLELDVAIVGGGPSGLVAGYFLAKAGHRVALYERKLSVGGGMWGGGMLFNEIVVQEDAKRLLDELDVPTLPYKEA
GYYTADSVETVSTITSKAVKAGLVVFNCISVEDVVVKDDRISGLVINWTAVPMANLHVDPLSIRSRYVIDATGHDTEVVAMVAKKAPGRLLTPSKNI
EGEKFMNPEEAERLTLENTKEVFPGLYVAGMACNATFGGPRMGPIFGGMLLSGEKVARLILKELSK

>Syntrophaceae bacterium PtaB.Bin038
MALNEVTISRAIVETYTKKLLAHLDVDVAVVGGGPAGLVAAYFLAGAGRKVALYERKLSIGGGMWGGGMMFNEIVVQAEAKGILDHFGVRTQEYAPG
YYTADAIEAVTTICSRAIQAGAKVFNCITVEDVVIRDNRVMGLVITWSPVEMTGLHVDPLTIHAKAVIDATGHDTEVLHVIER
KADVTLNTPTGKLMGERSMWSEKAERLTIDNTREICPGVYVAGMSANAAFGGPRMGPIFGGMLLSGRKVAEQILAGG

>Syntrophobacter sp. DG_60
MALDELIITQAIVERFSEKLKGCLMLDVAIVGAGPSGLVAGYYLAKNGHRVAIFEKKLSIGGGMWGGGMMFNQIVVQTEGKRILDEFEIKTAPFSEG
YYTADAIEAITTICSKACQAGVNVFNTISVEDVLVREGRVIGLVINWSTVEMAKLDVDPLTIRAQYVIEATGHATEVVKVIEKKMGESLLTPTGKII
GEKSLWAEIGEADTIKNTKEAYPGLFVCGMAANATFGSYRMGPVFGGMLLSGERVAKLIHERLRTKT

>Syntrophobacteraceae bacterium
MELNEITITQAIVDRFLEKFRNSLETDVAIVGGGPAGLVAGYFLAKAGCKVSLYERKLSVGGGMWGGGMLFNEIVVQEEAKRLLDLFGVGSHHYRDN
YYTADSVEAISTLTSQAVKAGVMIFNCISVEDVVMRPERVIGLVLNWTAVEMAGLHVDPLAIRAKFVVDATGHDVEVVRVVKRKVPGKLLTPSGEIE
GEKSMWSEVAEKLTLDNTREVFPGLYVAGMAANATFGGPRMGPIFGGMLLSGEKVAQTLIDQLKK

>Syntrophobacterales bacterium CG_4_8_14_3_um_filter_49_14
MELNEITITKAIIERFSEKLIACTEVDTAIVGGGPAGLVAAYFLAKVGKKVAIFEKKLSIGGGMWGGGMMFNEIIVQPEARELLDLFDVRTRKYEAG
YYSADAIEAVSTICSYATKAGARVFNCITVEDVMIREGRVIGLVINWTPVSMTGLHVDPLTIAAKSTIDATGHATEVLRVIERKTDIRLNTPSGALM
GERSMWADRAERLTLENTREICPGVYVAGMSANAAFGGPRMGPIFGGMLLSGKKVAELIIANG

>Syntrophus sp. (in: Bacteria)
MELNEITITRAIIERFTEKFLACTEVDVAIVGGGPAGLVASYYLAKAGRKVALFERKLSIGGGMWGGGMMFNEIVVQEEAKEILDLFGITSRPYETD
YHTADAIEAVSTICSYAVKAGAKIFNCMSVEDVMIREGRVTGLVITWSPVEMAGLHVDPMTIGAKWIIDSTGHATEVLRVIERKADVRLFTETGKLM
GERSMWAEKAERMTLENTKEICPGVYVAGMSANAAFGGPRMGPIFGGMLLSGRKVAEQILSR

>Thermanaerovibrio acidaminovorans (strain ATCC 49978 / DSM 6589 / Su883)
MELDERRISAVIVRRFMDRLLDSMDLDVAIVGGGPAGLVAGHNLAREGFKVAMFERKLSLGGGMWGGGMMFNQIVVQEEGAQVLREFGVRVLDEGEG
YYSADSVEAVSTLISSATRAGLRVFNCVTAEDVTMREDRVVGLVITWTPVEMAGLHVDPLAIRSRFVIDATGHDINVVRVVERKVPGKLMTPTGRAE
GEKSLWSHRAEELTLENTREVFPGLYVAGMSANATFGGPRMGPIFGGMLLSGRKAAQLVSRALRGQGGRG

>Thermincola ferriacetica
MHLDETVISRGIVQKYMEELMDYMNTEVAIVGGGPSGMVAAYYLVKRGCKVALFDRKLAVGGGMWGGAMMFNKIVVQSAGKRILDEFAISCEEYERG
YYVADAVESVTTIASMTVKAGCKIFNLIGAEDVMVEDGRVTGLVLNWTPVQVNNYHVDPLVVRAKYVIDGTGHPAEVTQTLTRKMGVRLNTPTGGVA
GEKPMNALKGELDVVENTREVFPGLYVTGMAANAAFGSHRMGPVFGGMLLSGEKAAMEIAARLGK

>Thermococcales archaeon 44_46
MLRDVTISRAIIETYFKELLEHLNLDVAIVGAGPSGMVAAYYLAKGGAKVAIFEKKLSIGGGIWGGGMGFNKIVVEEEAKEILEEFGVRHEEFEEGY
YVADAIEVATTIASKNIKAGAKIFNMVEVEDLVVKENRVAGIVINWTPVKMTDLHVDPLTVEAKFVIDSTGHGAQVTQLLEKRGLIERVPGESAMWA
EMGEKLTVEHTKEIYPGLYVTGMAANAVAGAPRMGPIFGGMFLSGRKAAFEILEKLKK

>Thermococcus celer Vu 13 = JCM 8558
MLKDVEVSRAIIEAYTKDILDSLKLDVAVVGAGPSGMVAAYYLARGGAKVAIFEKKLSVGGGIWGGAMGFNRIVVEESAREILDEFGVDYEEFKPGL
YVADAIEVATTMASKTVKAGVKVFNMVEVEDLVVKDGRVAGVVVNWTPVKMTGLHVDPLTVEAEFVIDSTGHGAQITGHLLKRGLIEELPGEGPMWA
EMGERLTVEHTKEVFPGLYVTGMAANAVAGAPRMGPIFGGMFLSGRKAALDILERLGV

>Thermodesulfatator autotrophicus
MALDEIKISRAIIERYFKKLTDYLEMDVAVVGAGPSGLMAAYKLASEGFKVAVFERRLSIGGGIWGGGMMFNEIVVQEEGARLLKEIGVRTEPWNGG
EYYTADAVEVACILAAKSVQAGAKIFNLIMVEDVMVRDNRVVGLVLNWSATEIAGLHVDPLAVKAKYVVEATGHETAVLQVMQKKLGAKLNTETGKV
MGEKSMWAEVAENLTVDYTREVYPGVFVAGMAANATFGAYRMGPIFGGMLLSGERVAQLIAERLRQ

>Thermodesulfobacterium geofontis (strain OPF15)
MELKIQRAIVKFGMEDLYEYSDVDVLIVGAGPSGLTSAKYLADKGFKVLVYEKRLSFGGGIGGGGNMIPKIVVQEEALPILKDFKIKYKEAEKNLYT
IDPAELIAKLAVGALDAGAKIILGVHVEDVIVRDNPPRVTGVLWRWTAIEISGLHVDPLYTQSKALIDATGHGAEIVQIAAEKNPELNIIIKGEKSN
WSEVSEKLVVDYTGKVAEGLYVTGIAVCEVFGLPRMGPIFGGMLMSGKKIAEIIEKDLRG

>Thermodesulfobium acidiphilum
MTKHFLNPVTDVNVSKLILKHYFESITDALTSDVIIVGGGPSGLTAARELGNSGYKVVIMERKLSPGGGTWGGSMSFNKVVIQKDLKDYLNELEIPF
VEDLDALVVDSCLFASQLIAKALKTQNVKLFNLMTVVDLEYTNNAITGVVVNNTGIEIAGLHVDPMVFQTKAVLDATGHDAIAANIYSKRVQLPLRK
EHFMNAVQGEEDTVNNTKMLANGLFVSGMAANNVDGGSRMGPIFGGMIKSGLKAAKLIMEYIKTV

>Thermodesulforhabdus norvegica
MAELNEIIITRAIIDRYHAKITGNLDVDVAIVGAGPSGLVAGYHLAQKGYRVTIFERKLSVGGGIWGGGMLFNEIVVQDEARRILEEFGVRVNRYEE
NYYTADAIETVSLLAARAIQKGVTILNGITVEDVVMRPNRVIGLVILWSAVEIAGLHVDPLAIRAKYIVDATGHDTEVVKVVHKKVPGRLMTPTGNI
EGEKSMWSEEAEKLTLENTREVFPGLFVAGMAANATFGGPRMGPIFGGMLLSGEKVAHLIDERLKQGS

>Thermoplasmata archaeon HGW-Thermoplasmata-1
MTVIDEIVVTRKIFDRFSREFLDHLDVDVALVGGGPANMVAAHYLAKAGKKVVLFERKLAPGGGMWGGGMNFPVIVIQEAALPIMNEFGIKVEGSND
GYYTADSVECVAKLLAKSIDSGARVFNSMTVQDVMIRDDDNKVSGVVINWTGVDITKMHVDPLTIRAKFVVDGTGHPCEVCNVVAKKAGRLRTPSGK
VEGERSMWAEVGEETTVNNTVEIYPGLYVAGMAANGVMGAPRMGPIFGGMLLSGKKVAEMILQKLGA

>Thermoprotei archaeon ex4572_64
MSIVRKVSEDEISKAIINEALKELESLVDVDAVVVGSGPSGLTCSYYLAKYGLKTVLIERRLSFGGGIGGGGMLLPSIAIESPAAELIHDEFLVNIK
KVRDGLYVMNPAEFIAKLASKAINAGVKVLLGVSVEDVIFRSNPLRIAGVVINWSAVHISQLWVDPLFIKAKAVIDATGHDAEVVNIVSKKIPDFKL
AIKGEKSACSIEAEDLIISYSGKVVEGLYVTGMATAKVYGLPRMGPIFGGMVLSGKKTAEEVYRDLSRELNR

>Thermoproteus uzoniensis (strain 768-20)
MRKINLIWKLRGAMELKIGRAIIRHGAEDLYEYSDVDVAIVGAGPSGLTAARYLAEKGLKVIVLERRFSFGGGIGPGGNMYPKIIVQEEALPILRDF
KVRYKPAGDGLYAVDPAELIAKLAAGAIDAGAKILLGVHVDDVIFRGDPPRITGLLWIWTPIQMSGMHVDPLYIQTKAVVDATGHDAEVVSVAARKV
PELGIQLQGEKSAWSEVSEKLVVEHTGKVAPGLYVAGMAVAAVFGLPRMGPIFGGMLMSGKKVAEIVAKDLAAEVHAV

>Thermosipho africanus
MWDYEVSKIIVERFFEKLNDNLNVDVAIVGGGPSALSASYYLSKKGLKVAIFEAKNEPGGGTWGGGMMFNELVVENDIKSFLDELGMNYLIKDNFIS
VDSVHFASSLLYNATKAGAVLFNNVIVEDIAFYENKVNGIVINWAPVIRQKLHVDPITIMAKFVVDGTGHPANVVNMLVDRGIDIDLPIGKIREYPM
NAKEGEKFVVENTKEVFPGLYVMGMAAVSVGGGPRMGPIFGGMIKSGLKVAKEILEKLSI

>Thermosipho melanesiensis (strain DSM 12029 / CIP 104789 / BI429)
MWDLEISKIIVNGFFEKFNDALDVDVAIVGGGPSALTASYFLTKNGFKVVIFEEKNDPGGGTWGGGMLFNELVVEEELEWMLKEFGMNYKRLNGFIS
IDSVHFASSLLYNTTKVGTKIFNNVIVEDILMEENRLCGVVINWAPVIKQRLHVDPITVKAKYVVDGTGHPASVVQMIIDRNLEVELPLDKIREFPM
NAKEGENFVLKNTKEVFPGLFVMGMAAVSVGGGPRMGPIFGGMLKSGEKVANAIVEKLSVEVSK

>Thermosulfidibacter takaii (strain DSM 17441 / JCM 13301 / NBRC 103674 / ABI70S6)
MLDEKIITKAIIESYTQNLLDYIDMDVAIVGAGPAGLTCAYYLAKEGVKVGIFERKLSIGGGIWGGGAMFNEIVLQEEALPIVQEMEVSYKPYKEKG
YYVINAVEFACALGLKAIRAGAKIFNLWSAIDVKVKGEDERVNGLVLLWTPVDTAGLHVDPITVEAKYVVDGTGHDAEIANVVVKKLKKKLATPTGD
VAGERPMWAEFGEKATEEFTGEVYPGLFVIGMAAVACYGKHRMGPIFGGMLLSGKKAAKMILECLK

>Thermosulfurimonas dismutans
MALDEVKITQAIVERFTEKLKEALELDVAVVGAGPSGLMAAYKLAKEGFKVAIFERKLSIGGGMWGGGMMFNEIVVQEEGARLLKEIGVEARPWQED
YYTADSVETVCALGYAARKAGAMIFNLISVEDVMVRKDRVVGLVINWTAVEMGGLHVDPLAIRSKYVVESTGHELSVLHIMQKKLGVQLMTPSGKIE
GEKSLWADVAETTTLENTREVFPGVFVAGMAANATFGSYRMGPIFGGMLLSGEKVAQEIAARLKK

>Thermosyntropha lipolytica DSM 11003
MVINDIKITRSIIEEYYAFTRDFLDCDVVIVGGGPAGMTAAYYTAQQGLRTVVLESRLSPGGGMWGGGMFFNQIVFQPEAGEILQELGISYTANREG
YLVVPSYRAVASLILAADRAGARILNGITAEDIMVRENRVCGVVINWTAAVKLGMHVDPLCIGGKVVIDATGHDAGIVRTYLDKSGGSLPEDEEERI
RTSSMWAAKGEEMVVEYTRFITEGLIACGMSVSSLFNTPRMGPIFGGMLFSGRKAAELALDYIRKVKA

>Thermovirga lienii (strain ATCC BAA-1197 / DSM 17291 / Cas60314)
MKLDEKIISKAIITRYYQKILSHLQVDVAIVGGGPSGLVAGYYLAKEGHRVALFERKLSVGGGMWGGGMLFNEIVVQEDAKEILEDFGVRVQPWEDA
GYYTADAIESVCSITSKAIQAGLTVFNCISVEDVSVEGDRITGLVINWTPVEMSGLHVDPLSIGASFVIDATGHDTEVVHMVAKKAPGKLMTPSGDI
EGEKFMCPDEAEKKTVENTKEVFPGLYVAGMACNATFGGPRMGPIFGGMLLSGRKVAALISQRLK

>Treponema sp. CETP13
MLEYNVSKGILDSYHTKLKSALDSDAIIVGSGPSGLVAGYFLAKAGKKVVMFERELAPGGGIWGGGMFFNDVMVQEEAATILSEIGVELPEVKDNFY
TIDSVYLASTLISKAVEAGVTLLNMISIEDIIFAKDESIGGVVLNWAPVHKEHMHVDPLMAISRCVLDATGHPSEIVNVLTRKNEITLNTKTGKVMG
ERSLKCKKAELATAENTCEIYPRLFVSGMAANGVAGAYRMGPVFGGMIRSGKKVAEQMLQCIDTEAPIYD

>Vulcanisaeta distributa (strain DSM 14429 / JCM 11212 / NBRC 100878 / IC-017)
MAGIYISESSITRAIMRSALKMLDEYSSVDVAIVGAGPSGMTAAYYLAKAGLKTIVLERRFSFGGGIGGAASHLPSIVVEYPASDILSKDFGVRLQD
MGDGLFAVDPAEMIAKLAVRAIDAGAKFLLGVHVDDVIIRDNPPRVAGLAVYWSTVQMAGVHTDPFFIEAKAVVDATGHDAEVAAVTTRKNPDLGLA
IHGEKSAHASVAEDLVVKYTGRVMEGLYVTGMAVAAVYGLPRMGPIFGSMIMSGKRVAELIINDLRR

>Zestosphaera tikiterensis
MEPLEAKISKIIWKETLNDWLKLSNVDVVVVGAGPSGMVTAKYLADSGIKTLVLERRLSFGGGIGGGGMLMHKVVVDSKALNILDDFKIKYSRSDYE
GLYVVDASELMAKLAAGAIDSGAKIVNGITVEDLIVRDNPFRVEGVVIQWSAVNLSGLHVDPLFIYSKAVVDATGHDAEVLKVLSRKNPEVNLKIPG
EKSAYAELSEELVVKHSGKVLPGLYVSGMAVAALYGIYRMGPIFTGMLLSGKKVAEEIAKDLRGGSQ

Supplementary Table 4 Occurrence of ROS defense genes in genomes of prokaryotes whose non-Cys THI4s have crystal structures

The predicted proteomes in NCBI were searched by BlastP with the indicated query sequences. Significant hits (e-value < 1e-05) are indicated with a plus sign.

**Enzyme**	**Query[a]**	**Organism**			
		Thermovibrio ammonificans	*Methanococcus igneus*	*Methanococcus jannaschii*	*Methano-thermococcus thermolithotrophicus*
Catalase-peroxidase	WP_013536945	+	-	-	-
Cytochrome *c* peroxidase	WP_013537906	+	-	-	-
Cytochrome *bd* complex	WP_013537252<br>WP_013537253	+	-	-	-
Heme-catalase	WP_000077872	-	-	-	-
Mn-catalase	WP_000488336	-	-	-	-

[a] GenBank identifier.
